# Polyadenylated versions of small non-coding RNAs in *Saccharomyces cerevisiae* are degraded by Rrp6p/Rrp47p independent of the core nuclear exosome

**DOI:** 10.15698/mic2024.05.823

**Published:** 2024-05-22

**Authors:** Anusha Chaudhuri, Soumita Paul, Mayukh Banerjea, Biswadip Das

**Affiliations:** 1 Present Position: Zentrum fǜr Molekulare, Medizin, Institut fǜr Kardiovaskuläre Regeneration, Haus 25B, Goethe-Universität, Theodor-Stern-Kai 7, Universitätsklinikum, 60590 Frankfurt am Main, Germany.; 2 Department of Life Science and Biotechnology, Jadavpur University, 188 Raja S.C. Mullick Road, Kolkata – 700 032, West Bengal, India.

**Keywords:** rRNA, snoRNA, snRNA, Nuclear Exosome, Rrp6p, Rrp47p, Mpp6p, Nuclear RNA turnover

## Abstract

In *Saccharomyces cerevisiae*, polyadenylated forms of mature (and not precursor) small non-coding RNAs (sncRNAs) those fail to undergo proper 3′-end maturation are subject to an active degradation by Rrp6p and Rrp47p, which does not require the involvement of core exosome and TRAMP components. In agreement with this finding, Rrp6p/Rrp47p is demonstrated to exist as an exosome-independent complex, which preferentially associates with mature polyadenylated forms of these sncRNAs. Consistent with this observation, a C-terminally truncated version of Rrp6p (Rrp6p-ΔC2) lacking physical association with the core nuclear exosome supports their decay just like its full-length version. Polyadenylation is catalyzed by both the canonical and non-canonical poly(A) polymerases, Pap1p and Trf4p. Analysis of the polyadenylation profiles in WT and *rrp6*-Δ strains revealed that the majority of the polyadenylation sites correspond to either one to three nucleotides upstream or downstream of their mature ends and their poly(A) tails ranges from 10-15 adenylate residues. Most interestingly, the accumulated polyadenylated snRNAs are functional in the *rrp6*-Δ strain and are assembled into spliceosomes. Thus, Rrp6p-Rrp47p defines a core nuclear exosome-independent novel RNA turnover system in baker's yeast targeting imperfectly processed polyadenylated sncRNAs that accumulate in the absence of Rrp6p.

## INTRODUCTION

In baker's yeast *Saccharomyces cerevisiae*, ribosomal RNAs (rRNAs), small nuclear RNAs (snRNAs), and small nucleolar RNAs (snoRNAs) comprise the major class of untranslated RNAs that serve diverse functions at distinct phases of the gene expression pipeline. The rDNA locus encoding rRNAs consists of a 9.1 kb long DNA segment that expresses 100 to 200 copies of the 35S rRNA precursor unit. Each 35S rRNA precursor unit consists of 18S, 5.8S, and 25S rRNA genes and two non-transcribed spacer units (NTSs) separated by the 5S rRNA (reviewed in Refs. [1] and [2]). The 5S rRNA gene is transcribed in the opposite direction by RNA polymerase III to generate a precursor that is extended by ten nucleotides at its 3′-end. Their maturation involves exonucleolytic removal of the 3′-end extension by the product of the *RNA82* gene [3] and several modification events.

Two other prominent classes of small non-coding RNAs (sncRNAs), as exemplified by snRNAs and snoRNAs, encode small nuclear RNAs (comprise the RNA components of spliceosome machinery) and small nucleolar RNAs (serve as the guide RNAs for site-specific modification events in the ribosomal RNAs during their maturation). All snRNA genes (U1, U2, U4, and U5), except U6 snRNA, are transcribed by RNA polymerase II, whereas the latter is transcribed by RNA polymerase III. The formation of the 3′-ends of the snRNA primary transcripts is a crucial step in their maturation event that is mediated co-transcriptionally by the trimeric Nrd1p-Nab3p-Sen1p (NNS) complex [4, 5]. The NNS complex coordinates recruitment of the nuclear exosome and Rrp6p, which trim the long 3'-extensions of these precursor snRNAs to their mature length [4, 5]. snoRNAs consisting of a group of untranslated RNAs are classified into two classes, box C/D and box H/ACA [6–8]. They are the transcripts of RNA polymerase II and are either transcribed as individual genes or generated from intron (reviewed in Ref. [6]). Mature 3′-ends of individually transcribed snoRNAs are coupled to their transcription termination [9, 10] and is mediated by the NNS complex in association with a few cleavage polyadenylation components [9, 10]. Following their transcription, RNA-DNA helicase Sen1p catalyze the dissociation of the RNA from the DNA template [11], leading to further recruitment of Pcf11p, the nuclear exosome, the TRAMP, and Rrp6p to their extended 3′-terminus [12–15] resulting in its trimming until the complex reaches the snoRNA secondary structure [16]. 3′-end maturation of the intronic snoRNAs, in contrast, is initiated by debranching of the intron lariat by Dbr1p and Rnt1p [17–19] that promotes snoRNA release, followed by their exonucleolytic processing at the 3′-end. The NNS complex subsequently binds to the 3′-end of these debranched RNAs to stimulate their 3′-end processing [20], although the involvement of the exosome/Rrp6p in this process is uncertain [6].

The nuclear exosome, implicated in the 3′-end processing events of these non-coding RNA precursors and processing intermediates, consists of nine core subunits arranged in a two-layer stacked donut-like structure with a common central channel. Three cap subunits, Rrp40p, Rrp4p, and Csl4p, are placed as a trimeric cap on top of the remaining six subunits, Rrp41p, Rrp42p, Mtr3p, Rrp43p, Rrp45p and Rrp46p, which collectively form the bottom hexameric ring structure [21]. Strikingly, the core exosome (Dubbed Exo-9) lacks the catalytic activity for RNA hydrolysis despite the presence of the putative active site for 3′→5′ exoribonuclease in six of the nine core subunits [21, 22]. The catalytic activity of the entire exosome complex resides in the tenth and eleventh subunits, Dis3p/Rrp44p and Rrp6p, respectively, each of which makes contact with the Exo-9 structure from opposite sides [22, 23]. While Dis3p/Rrp44p is associated with both nuclear and cytoplasmic forms of the exosome, Rrp6p is specifically associated with the nuclear form [24, 25]. Additionally, the exosome is associated with several ancillary factors, Lrp1p/Rrp47p and Mpp6p (both specific to the nuclear exosome), and the *SKI* complex (specific to the cytoplasmic exosome) [26]. Two ancillary protein complexes, the TRAMP and the CTEXT in *S. cerevisiae* bring about the catalytic specificity of the exosome, and together with these complexes, the exosome targets distinct sets of RNA substrates. The TRAMP (**TR**f4p/5p-**A**ir1p/2p-**M**tr4p-**P**olyadenylation) complex consisting of non-canonical poly(A) polymerase, Trf4p/Trf5p, a zinc knuckle protein Air1p/Air2p, and the RNA helicase Mtr4p [14, 27–29] assists the exosome to target faulty mRNA transcripts generated at the earlier phase of mRNP biogenesis [30] and in the processing/modification events of other non-coding RNAs. The CTEXT (**C**bc1p-**T**if4631p-dependent **EX**osomal **T**argeting; previously termed as DRN) [30, 31], in contrast, consists of nuclear cap-binding protein Cbc1p/2p [32, 33], shuttling proteins Tif4631p/Upf3p [34], a DEAD-box RNA helicase, Dbp2p [31], which collectively participate in the degradation of aberrant mRNAs produced at the late phase of mRNP biogenesis [30, 31].

The “Core exosome model,” subsisting for a long time, postulated the obligatory and collective contribution of each of the eleven exosome subunits for this complex's structural integrity and catalytic activity in all RNA metabolic functions [23, 24, 35–42]. However, a large body of experimental data substantiate evidence that was not predicted and anticipated by the ‘core exosome model’ [25, 43, 52, 44–51]. Instead, these pieces of evidence strongly support the idea that individual subunits of the core exosome may physically exist either as a monomer or as a separate complex in association with exosomal or non-exosomal components [53–57]. Furthermore, many exosome components function independently of the core exosome by targeting distinct sets of RNA substrates [28, 46, 47, 50, 57, 58], which are not targeted by the entire exosome complex. In addition, the intracellular localization profiles and copy numbers of the individual exosomal subunits vary, thereby indicating that individual subunits may have Exo-11 complex-independent functions [59–62]. Collectively, these findings led to an alternative ‘exozyme’ hypothesis that demands that in addition to its role as a part of the exosome complex, some components assemble into and function as independent complexes [45]. In good agreement with the ‘exozyme’ concept, the nuclear exosome component Rrp6p alone was shown to process the 3′-end of the 5.8S+30 RNA intermediate [52] and degrade the polyadenylated version of rRNAs [63]. However, whether the Rrp6p-dependent decay of polyadenylated rRNA represents an independent function from the core exosome is currently unknown [63]. A later study indeed revealed that some of the processive activities of Rrp6p occur independently of the core exosome [47], which was supported by the finding that cells depleted of Rrp6p accumulate poly(A)^+^ rRNA degradation intermediates different from those found in cells lacking either Dis3p or Rrp43p [47]. Strikingly, disrupting the physical interaction between Rrp6p and the core exosome did not affect the processing of the 3′-end of 5.8S rRNA and snoRNAs and some of these Rrp6p-specific decay intermediates. Collectively, these findings affirmed that Rrp6p can perform several processing and degradation activities independently of the core exosome [47].

A consistent observation involving a dramatic enhancement in the steady-state levels of the total (nonadenylated and polyadenylated pools) population of 5S/5.8S rRNAs exclusively in *rrp6*-Δ yeast strain during the experiments involving traditional mRNA decay inspired this investigation. Notably, 5S/5.8S rRNAs (transcripts of RNA Pol III and Pol I, respectively) are typically used as internal controls in these experiments. Although initially the finding was considered an artifact, this observation proved very reproducible in diverse genetic strain backgrounds and was reminiscent of the Rrp6p-dependent trimming/processing of the polyadenylated form of rRNAs [14, 16, 24, 63–67]. These earlier studies collectively established that Rrp6p is involved in the 3′-end trimming/processing of 5.8S rRNAs, sn- and sno-RNAs in the wild type (WT) strain and presumably, polyadenylated species of the precursor forms of these non-coding RNAs accumulated in the *rrp6*-Δ mutant strain. It should be noted here that none of these earlier studies addressed clearly (i) if Rrp6p trims/degrades the mature forms of these non-coding RNAs, (ii) if Rrp6p alone is involved in this trimming/degradation activity or it requires any ancillary factor(s), (iii) if this trimming/degradation activity of Rrp6p requires the involvement of the core nuclear exosome or it is exclusively independent of core nuclear exosome and (iv) polyadenylation profiles of these non-coding RNAs in WT and *rrp6*-Δ yeast strains. In this investigation, we address if Rrp6p carries out the degradation of the polyadenylated mature and/or precursor forms of non-coding RNA species (rRNAs, snRNAs, and snoRNAs) independently of the core-exosome. Using a systematic analysis, we determine the relative functional contributions of the Rrp6p, Rrp6p-associated subunits Rrp47p, Mpp6p, and a few other components of the core nuclear exosome, the TRAMP, and the CTEXT in degradation of the total and polyadenylated pools of mature/precursor rRNAs, sn- and snoRNAs. Our investigation revealed that a dramatic accumulation of polyadenylated versions of the mature forms (besides their precursor forms) of the sncRNAs (5S and 5.8S rRNAs, snRNAs, and snoRNAs) takes place in the *rrp6*-Δ and *rrp47*-Δ strains but not in the strains carrying the mutant alleles of the core exosome, the TRAMP, the CTEXT, and Rrp6p-associated Mpp6p. This finding suggests that Rrp6/47p coordinates the degradation of the polyadenylated forms of mature sncRNAs independent of the core exosome. Polyadenylation of the sncRNA species requires the involvement of both the canonical and non-canonical poly(A)-polymerase, Pap1p, and Trf4p. Most importantly, our data showed that polyadenylated versions of scarce amounts 5S and 5.8S rRNAs with either few nucleotides extended or recessed ends are detectable in the WT strain indicating that the fraction of these rRNAs that fail to achieve perfectly mature 3′-end are targeted by Rrp6p/47p. Remarkably, adenylated snRNAs that accumulate in *rrp6*-Δ strain are functional and assembled into functional spliceosomes. Thus, in this investigation, we demonstrate that accumulation of the mature form of polyadenylated sncRNAs in the *rrp6*-Δ mutant strain results from their diminished degradation in absence of functional Rrp6p apart from their processing defects in the same strain as reported in many earlier studies.

## RESULTS

### Steady-state levels of 5S, 5.8S rRNAs, snRNAs, and selected snoRNAs display a dramatic enhancement only in an *rrp6*-Δ yeast strain

**Figure 1.**
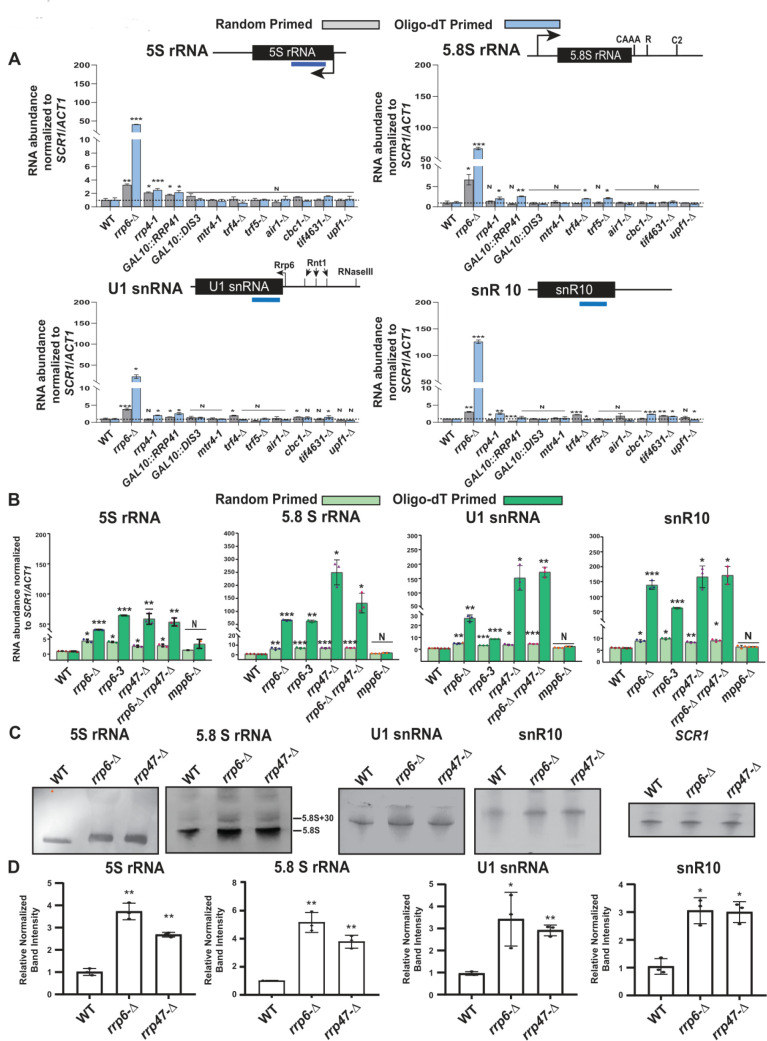
FIGURE 1 (A) Polyadenylated versions of small non-coding RNAs accumulate in an* rrp6*-Δ yeast strain. Histograms revealing the steady-state levels of 5S, 5.8S rRNAs, U1 and snR10 RNAs estimated from 2 ng cDNA samples prepared using either random hexanucleotide primers (grey bar) or oligo-dT_30_ anchor primer (blue bar) by RT-qPCR assay with the amplicons corresponding to their mature sequence from the indicated isogenic strains. The *upf1*-Δ strain was used as a negative control. **(B-D) Characteristic steady-state levels of sncRNAs require the functional exonuclease domain of Rrp6p and ancillary nuclear factor Rrp47p/Lrp1p, but do not require Mpp6p. (B)** Histogram revealing the steady-state levels of 5S, 5.8S, U1, and snR10 RNAs estimated by RT-qPCR from the 2 ng cDNA samples prepared using either random hexanucleotide primers (pale green bars) or oligo-dT_30_ anchor primer (deep green bars) from the indicated isogenic yeast strains. For histograms presented in panels A and B, *SCR1* (in the case of Random Primer) and *ACT1* mRNA (in the case of Oligo dT_30_ Primer) were used as the internal loading control. Normalized values of each of the ncRNAs in the WT yeast strain were set to one. Three to Four independent cDNA preparations (biological replicates, n = 3, in some cases 4) were used to determine the levels of various ncRNAs. The statistical significance of difference reflected in the ranges of P values estimated from Student's two-tailed t-tests for a given pair of test strains for every message are presented with the following symbols, *<0.05, **<0.005, and ***<0.001; N, not significant. **(C)** Northern blots revealing the steady-state levels of 5S, 5.8S, U1, and snR10 RNAs in WT, *rrp6*-Δ and *rrp47*-Δ strains. Total RNA samples isolated from isogenic WT) and strains carrying *rrp6*-Δ, and *rrp47*-Δ yeast strains, separated on a 15% denaturing acrylamide gel and analyzed by northern blotting using DIG-labelled oligonucleotide probes corresponding to the mature regions of these rRNAs as described in materials and methods (See Supplementary Table S5). *SCR1* RNA was used as a loading control. **(D)** Quantification of northern hybridization data for 5S, 5.8S, U1 and snR10 RNAs from panel B. Individual ncRNA levels were normalized to corresponding to *SCR1* RNA signal. Normalized values of each rRNA in the WT yeast strain were set to one.

The steady-state levels of both total and polyadenylated fractions of rRNAs in WT and the strains carrying mutations in components of the core exosome (*rrp6*-Δ, *rrp4-1*, *GAL10::DIS3,* and *GAL10::RRP41*), the TRAMP (*mtr4-1*,* trf4*-Δ, *trf5*-Δ, and *air1*-Δ), and the CTEXT (*cbc1*-Δ and *tif4631*-Δ) were determined by RT-qPCR and northern blot analysis. The RT-qPCR-assay employed either random hexanucleotide primed (amplifies total cellular pool) or oligo-dT primed (amplifies polyadenylated pool) cDNA for quantitative amplification of the mature sequences of the rRNAs (Supplementary Fig. S1A, see **Table 1** for the amplicon locations and primer sequences). Preliminary data from these experiments revealed that the steady-state levels of total cellular populations of 5S and 5.8S rRNAs (determined using random hexanucleotide primed cDNA samples) displayed significant enhancements in *rrp6*-Δ strains belonging to four different genetic backgrounds relative to their isogenic WT (Figs. S1B). Extension of this finding further unveiled that the levels of both total and polyadenylated versions of 5S/5.8S rRNAs, all snRNAs and select snoRNAs were dramatically enhanced in the *rrp6*-Δ strain relative to the WT strain (**Figs. 1A**, S2A and **Table 1** and 2). Most remarkably, their enhancements in the steady-state levels were displayed only in the yeast strain carrying the *rrp6*-Δ allele and not in the strains carrying mutations/deletions in the components of core exosome/TRAMP/CTEXT complexes (**Figs. 1A**, S2A and **Table 1** and **2**). Only a marginal increase (~1.6 to 3.0 fold) in the levels of total pool (determined using the random primed cDNA) of 5S rRNA (**Fig. 1A** 5S panel, and **Table 1** and **2**), and some sn-/snoRNAs were noted in *rrp4-1*, *GAL10::RRP41, GAL10::DIS3* and *trf4*-Δ/*trf5*-Δ yeast strains (Fig. S2A and **Table 1** and **2**), which is insignificant relative to their enhancement in the *rrp6*-Δ strain. This finding indicates that impairment of several specific components of the core exosome may have marginal impact on the cellular repertoire of 5S rRNA. Furthermore, the steady-state levels of neither total (signals from random-primed cDNA) nor the polyadenylated (signals from oligo-dT-primed cDNA) fractions of 18S and 25S rRNAs showed any significant alterations in any of these strains (Figs. S1C and **Table 1** and **2**). Notably, the polyadenylated fractions of all of the 5S, 5.8S rRNAs, sn- and snoRNAs (estimated using oligo-dT primers) were found to undergo substantial enrichment in the *rrp6*-Δ strain relative to the levels of augmentations of their total fractions (**Figs. 1A**, S2A). Collectively, these findings support the argument that deletion of Rrp6p leads to the accumulation of predominantly the polyadenylated forms of these sncRNAs as reported in a number of earlier studies [14, 16, 24, 63–67].

**Table 1.** Amplicon specificity, location, and extent of steady-state enhancements of various ncRNAs in WT (*RRP6*^+^) and *rrp6*-Δ yeast strains using the oligo-dT-primed cDNA.

**RNA**	**Amplicon**	**Location (with respect to CDS)**	**Amplicon size**	**Identity of the amplicons amplified (Mature/Precursor)**	**Abundance**	**Net Fold Enhancement of the Mature Polyadenylated Species in the *rrp6*-Δ strain relative to the WT**
5S	M5SA	+1 to +59	59 bp	(Mature+Precursor) 5S	90-150 fold	50 - 150 fold
	M5SB	+60 to +121	62 bp	(Mature+Precursor) 5S	50 fold	
	Pre-5S C	+168 to +235	68 bp	Precursor 5S	0.6 fold	
5.8S	M5.8S	-8 to +158	166 bp	(Mature+Precursor) 5.8S	125 fold	90 fold
	Pre-5.8S-C	-8 to +188	196 bp	Precursor [(5.8S+30) +7S+27S]	36 fold	
	Pre-5.8S-D	-8 to +295	303 bp	Precursor (7S+27S)	3 fold	
	Pre-5.8S-E	-8 to +378	386 bp	Precursor27S	2 fold	
snRNA U1	M snRNA U1 A	+128 to +184	57 bp	(Mature+Precursor) snRNA U1	30 fold	7 - 23 fold
	M snRNA Ul B	+387 to +456	70 bp	(Mature+Precursor) snRNA U1	14 fold	
	Pre- U1-C	+536 to +640	105 bp	Precursor snRNA U1	3 fold	
	Pre- U1-D	+536 to +678	143 bp	Precursor snRNA U1	2 fold	
	Pre-Ul-E	+536 to +710	175 bp	Precursor snRNA U1	1.3 fold	
	Pre- U1-F	+696 to +818	123 bp	Precursor snRNA U1	1 fold	
snRNA U2	M snRNA U2 A	+118 to +190	73 bp	(Mature+Precursor) snRNA U2	45 fold	10 - 40 fold
	M snRNA U2 B	+650 to +716	67 bp	(Mature+Precursor) snRNA U2	24 fold	
	M snRNA U2 C	+1038 to +1104	67 bp	(Mature+Precursor) snRNA U2	16 fold	
	Pre- U2-A	+1192 to +1265	74 bp	Precursor snRNA U2	4 fold	
	Pre- U2-B	+1306 to +1363	58 bp	Precursor snRNA U2	0.9 fold	
snRNA U4	M snRNA U4 A	+33 to +98	66 bp	(Mature+Precursor) snRNA U4	45 fold	40 fold
	Pre- U4-B	+105 to +335	231 bp	Precursor snRNA U4	2.3 fold	
	Pre- U4-C	+105 to +365	261 bp	Precursor snRNA U4	1.5 fold	
	Pre- U4-D	+231 to +365	135 bp	Precursor snRNA U4	1.2 fold	
	Pre- U4-E	+294 to +365	72 bp	Precursor snRNA U4	1 fold	
	Pre- U4-F	+376 to +453	78 bp	Precursor snRNA U4	1 fold	
snRNA U5	M snRNA U5 A	+27 to +111	85 bp	(Mature+Precursor) snRNA U5	64 fold	15 - 60 fold
	M snRNA U5B	+27 to +189	163 bp	(Mature+Precursor) snRNA U5	50 fold	
	M snRNA U5C	+110 to +189	80 bp	(Mature+Precursor) snRNA U5	18 fold	
	Pre- U5-D	+206 to +262	57 bp	Precursor snRNA U5	4 fold	
	Pre- U5-E	+254 to +341	88 bp	Precursor snRNA U5	1 fold	
snRNA U6	M snRNA U6 A	+18 to +94	77 bp	(Mature+Precursor) snRNA U6	116 fold	113 fold
	Pre- U6-B	+61 to +141	81 bp	Precursor snRNA U6	2.3 fold	
	Pre- U6-C	+158 to +240	83 bp	Precursor snRNA U6	1 fold	
snR10	M snR10 A	+16 to +89	74 bp	(Mature+Precursor) snRN 10 A	122 fold	70 - 120 fold
	M snR10 B	+141 to +216	76 bp	(Mature+Precursor) snRN 10 B	70 fold	
	Pre snR10-C	+366 to +456	91 bp	Precursor snRN 10 C	1.4 fold	
snR13	M snRN13 A	+32 to +99	68 bp	(Mature+Precursor) snRN 13 A	99 fold	90 fold
	Pre snRN13-B	+48 to +146	99 bp	Precursor snRN 13 B	4 fold	90 fold
	Pre snRN13-C	+48 to +180	133 bp	Precursor snRN13 C	1.5 fold	
	Pre snRN13-D	+132 to +238	107 bp	Precursor snRN 13 D	1 fold	
	Pre snRN13-E	+176 to +238	63 bp	Precursor snRN 13 E	1.5 fold	

Since the RT-qPCR assay, using the primer-sets corresponding to the mature sequences of these RNAs amplified both the mature and precursor species of these ncRNAs, it remained obscure from above data if the mature or the precursor forms of these RNAs accumulate in the *rrp6*-Δ yeast strain. To resolve this issue, we performed a northern blot analysis using the total RNA samples from WT, *rrp6*-Δ, *rrp4-1*, *GAL10::RRP41* yeast strains with the oligonucleotide probe corresponding to the mature sequence of the 5S, 5.8S rRNAs, U1 and snR10 RNAs. A single band corresponding to the mature length of 5S rRNA, U1 snRNA and snR10 were detected in these strains, which is either marginally higher or equal in intensities in *GAL10::RRP41* and *rrp4-1* strains and were significantly higher in the *rrp6*-Δ strain (**Figs. 1C**, S1D and S2B). In the case of 5.8S rRNA, however, similar intensities of the 5.8S_S_ and 5.8S_L_ mature length RNA species were detectable in WT, *GAL10::DIS3*, *GAL10::RRP41*, and *rrp4-1* strains*.* In the *rrp6*-Δ strain, in contrast, significantly higher amounts of 5.8S_S_ and 5.8S_L_ species corresponding to the mature length 5.8S rRNA were found to accumulate along with the 5.8S+30 precursor species (Fig. S1D) as reported before [52]. Notably, the relative ratio of intensities of precursor 5.8S+30 and mature 5.8S bands appear lower in all of our northern blots in comparison to previously published data [52]. To verify if this deviation from the previous finding is consistent across the different genetic backgrounds, we carried out a northern blot analysis with the total RNA from previously used isogenic WT and *rrp6*-Δ strains from five different genetic backgrounds. The data from this experiment also revealed a similar level of precursor 5.8S+30 species in all strains with different genetic backgrounds thereby suggesting that the lower ratio of 5.8S+30 precursor and the mature species in our original experiment is consistent and reproducible (Fig. S2D). It should also be noted here that in some of the previous studies, the intensity of the mature 5.8S rRNA band is stronger than the 5.8S+30 precursor species (see Fig. 1E in reference [24], and Fig. 6C in Ref. [51]) that is consistent with our data (see discussion). Further, quantification of the relevant signals/bands associated with mature length 5S rRNA, U1 snRNA and snR10 RNAs revealed that their levels undergo approximately 3.8 to 4.2 fold enhancement whereas the intensity of the 5.8S rRNAs was increased by 5.2 fold respectively in the *rrp6*-Δ strain (Figs. S1E and S2C). Notably, the levels of 5S rRNA displayed a marginal increase of 1.4 and 1.5 fold enhancements in the *GAL10::RRP41* and *GAL10::DIS3* strains (Fig. S1D-E), which is very consistent with their fold enhancement in these strains as determined from RT-qPCR analysis (**Fig. 1A** 5S panel). Collective data from RT-qPCR and northern blot analysis thus revealed that the polyadenylated forms of mature sncRNAs accumulate specifically in the *rrp6*-Δ strain, whereas their abundances in the strains deficient in the core exosome components are not significantly different relative to that in the WT strain.

Notably, Rrp6p was reported to allosterically regulate the Dis3p and stimulate its RNase activity [68]. Thus, conceivably the augmentation of mature length sncRNA levels in the *rrp6*-Δ strain may be viewed as an indirect effect that might have happened via the down-regulated Dis3p activity. Furthermore, in the previous experiments we noted a marginal increase in the levels of several small ncRNAs in some of the yeast strains carrying mutations in the exosomal components, Rrp4p, Dis3p, and Rrp41p (**Figs. 1A**, S2A), which warrants a rigorous investigation on the possible roles of these exosomal components. Towards this, first we confirmed that the cellular levels of the mRNAs/proteins encoding some of the exosomal subunits, *RRP4*, *RRP41*, *RRP46,* and *DIS3* remained unaltered in the *rrp6*-Δ strain relative to WT (Fig. S3A-B). Next, we demonstrate that after inactivation of Rrp4p for a much-prolonged period (6 hours instead of 2 hours) at 37°C following a temperature shift of the ts *rrp4-1* yeast strain to 37°C led to only a moderate and sporadic enhancement (≈2-3 fold) of the polyadenylated versions of some of the sncRNAs in the *rrp4-1* strain (Fig. S3C). Further, inactivation of Dis3p and Rrp4p for as long as 24 hours in glucose-dependent repressing condition following the galactose induction again yielded a marginal increase in some of the sncRNAs in an irregular fashion (Fig. S3D and S3E). Finally, we showed that growing the *GAL10::DIS3* and *GAL10::RRP41* strains initially in presence of 2% galactose did not lead to any significant enhancement of the polyadenylated form of any of these sncRNAs (Figs. S3D and S3E). Although, intermittent increase in the steady-state levels of some of the ncRNAs were previously noted occasionally in *rrp4-1*, *GAL10::DIS3* and *GAL10::RRP41* yeast strains, none of which are comparable to the extent of their enhancements displayed by an *rrp6*-Δ strain (Fig. S3C-E). Data from these experiments therefore argue against any significant functional role of *RRP4*, *DIS3*, and *RRP41* genes in the nuclear degradation of the polyadenylated sncRNAs. All these findings, taken together, led us to deduce that Rrp6p possibly constitutes an exosome-independent nuclear degradation system that possibly degrades the polyadenylated version of these sncRNAs.

**Table 2.** Steady-state levels of various Mature (+Precursor) and Precursor Species of ncRNAs in WT and strains deficient in the components of the Nuclear Exosome, the TRAMP and the CTEXT as determined by RT-qPCR.

**Steady-State levels of non-coding Mature (+Precursor) RNAs as determined from oligo-dT primed cDNA**												
Mature RNA Amplicons	WT	*rrp6*-Δ	*rrp4-1*	*GAL10::R RP41*	*GAL1O:: DIS3*	*mtr4-1*	*trf4*-Δ	*trf5*-Δ	*air1*-Δ	*cbc-1*Δ	*tif4631*-Δ	*Upf1*-Δ
5S	1.02±0.31	40.93±0.36	2.50±0.22	2.15±0.26	1.09±0.17	0.83±0.26	0.57±0.14	1.07±0.06	1.16±0.41	0.87±0.13	1.56±0.08	1.16±0.42
5.8S	1.07±0.18	66.77±2.02	2.07±0.28	2.59±0.08	0.72±0.07	0.92±0.08	2.01±0.04	2.12±0.14	0.80±0.23	0.94±0.12	1.24±0.17	0.78±0.14
18S	1.03±0.28	0.87±0.24	0.58±0.01	1.28±0.31	1.35±0.17	1.00±0.12	0.42±0.01	1.01±0.03	0.64±0.15	0.62±0.16	0.63±0.04	0.76±0.05
25S	1.00±0.04	0.43±0.02	0.82±0.11	1.45±0.01	1.02±0.01	0.90±0.11	0.55±0.02	0.74±0.24	1.21±0.37	0.72884±0.01	0.71±0.07	0.62±0.01
snRNA U1	1.01±0.16	22.69±4.34	2.06±0.09	2.59±0.31	1.37±0.16	0.99±0.07	0.76±0.11	1.08±0.15	0.75±0.09	1.41±0.19	1.58±0.37	0.63±0.04
snRNA U2	1.00±0.12	34.98±3.73	2.20±0.01	1.49±0.23	1.02±0.31	1.56±0.12	0.50±0.09	0.96±0.05	0.77±0.00	1.83±0.06	1.10±0.05	0.67±0.03
snRNA U4	1.00±0.02	35.78±3.84	2.72±0.41	1.89±0.27	1.23±0.07	1.15±0.04	0.90±0.09	1.31±0.04	1.39±0.67	1.25±0.20	2.23±0.05	0.89±0.12
snRNA U5	1.00±0.08	60.03±19.03	0.97±0.14	1.69±0.33	0.93±0.02	0.62±0.02	0.45±0.15	1.14±0.13	1.42±0.01	1.23±0.34	1.09±0.10	0.69±0.02
snRNA U6	1.00±0.16	113.57±13.02	2.01±0.45	2.73±0.25	0.92±0.15	1.06±0.03	0.76±0.10	0.92±0.08	0.67±0.20	2.84±0.31	2.93±0.32	0.70±0.09
SNR 10	1.00±0.02	125.78±3.22	2.56±0.23	1.39±0.37	0.87±0.10	1.24±0.37	0.76±0.05	0.80±0.12	0.69±0.31	2.39±0.04	1.73±0.09	0.73±0.04
SNR 13	1.00±0.08	89.55±4.13	2.30±0.10	1.60±1.10	0.83±0.08	1.03±0.07	0.46±0.07	0.67±0.04	0.63±0.08	2.18±0.10	0.75±0.08	0.99±0.15

**Steady-State levels of non-coding Mature (+Precursor) RNAs as determined from random Primed cDNA**												
Mature RNA Amplicons	WT	*rrp6*-Δ	*rrp4-1*	*GAL10::R RP41*	*GAL1O:: DIS3*	*mtr4-1*	*trf4*-Δ	*trf5*-Δ	*air1*-Δ	*cbc-1*Δ	*tif4631*-Δ	*Upf1*-Δ
5S	1.01±0.22	3.26±0.14	2.10±0.11	1.78±0.10	1.60±0.33	1.03±0.05	1.16±0.31	1.03±0.21	0.66±0.18	1.48±0.03	1.04±0.10	0.97±0.16
5.8S	1.05±0.37	6.73±1.29	1.38±0.07	0.71±0.14	0.85±0.20	1.19±0.28	0.83±0.03	1.01±0.17	1.01±0.15	1.01±0.21	1.09±0.09	0.99±0.02
18S	1.00±0.04	0.75±0.03	0.59±0.05	1.09±0.09	1.05±0.29	1.07±0.24	0.96±0.02	1.38±0.01	1.28±0.19	0.69±0.06	0.54±0.05	0.98±0.15
25S	1.01±0.22	0.40±0.01	0.63±0.09	1.073±0.07	1.08±0.09	1.16±0.01	1.08±0.11	1.11±0.20	1.03±0.26	0.74±0.06	0.75±0.02	1.00±0.08
snRNA U1	1.01±0.18	3.80±0.30	0.83±0.18	1.54±0.08	1.46±0.31	1.13±0.20	2.15±0.32	0.60±0.18	1.21±0.44	1.59±0.03	0.95±0.19	0.75±0.08
snRNA U2	1.00±0.08	11.76±1.31	1.84±0.07	1.26±0.26	0.99±0.24	0.85±0.05	3.26±0.65	0.72±0.17	1.09±0.19	1.30±0.13	1.27±0.09	1.13±0.09
snRNA U4	1.00±0.08	3.34±0.31	1.18±0.15	0.83±0.15	1.44±0.21	1.20±0.28	1.99±0.11	1.44±0.23	1.30±0.23	0.92±0.40	0.79±0.14	0.63±0.03
snRNA U5	1.00±0.09	16.51±0.65	1.38±0.05	1.45±0.14	0.86±0.04	1.00±0.05	2.21±0.08	0.53±0.20	1.17±0.04	1.19±0.07	1.33±0.39	1.20±0.06
snRNA U6	1.02±0.26	6.65±1.93	1.11±0.28	0.62±0.16	1.01±0.08	1.13±0.19	2.13±0.3	1.07±0.25	2.09±0.6	0.921±0.12	1.65±0.06	1.81±0.12
SNR 10	1.00±0.03	3.06±0.14	0.73±0.04	0.46±0.01	1.00±0.14	1.23±0.10	2.26±0.04	0.95±0.19	1.89±0.69	1.08±0.12	1.93±0.11	1.47±0.26
SNR 13	1.00±0.03	10.63±1.53	0.85±0.31	1.02±0.29	1.54±0.27	1.09±0.13	3.11±0.15	0.95±0.16	0.84±0.26	0.61±0.09	1.28±0.04	1.67±0.12

**Steady-State levels of small non-coding precursor RNAs as determined from random primed cDNA**												
**Precursor RNA Amplicons**	**WT**	***rrp6*-Δ**	***rrp4-1***	***GAL10::R RP41***	***trf4*-Δ**	***trf5*-Δ**	***air1*-Δ**	***cbc-1*Δ**	***tif4631*-Δ**	***Upf1*-Δ**		
pre 5S	1.02±0.27	0.77±0.42	0.84±0.13	0.65±0.23	0.68±0.12	2.04±0.92	1.55±0.76	1.38±0.75	2.69±0.27	0.52±0.11		
pre 5.8S	1.04±0.34	79.80±6.98	2.22±0.41	3.08±0.15	0.53±0.03	1.02±0.13	0.72±0.24	1.60±0.89	1.64±0.49	0.54±0.12		
pre 5.8S II	1.07±0.45	7.32±0.77	2.23±0.3	1.60±0.03	0.82±0.25	0.96±0.37	2.54±0.50	1.31±0.42	2.68±0.3	0.65±0.12		
pre U1 C	1.00±0.06	2.27±0.21	0.86±0.06	1.54±0.06	0.39±0.02	1.58±0.22	1.71±0.23	0.74±0.13	1.12±0.50	1.48±0.44		
pre U1 D	1.00±0.06	2.06±0.10	1.18±0.05	0.90±0.24	0.42±0.08	0.82±0.14	1.47±0.14	0.66±0.02	0.82±0.06	1.55±0.21		
pre U1 E	1.000±0.03	1.71±0.21	0.86±0.28	1.26±0.37	0.52±0.06	1.38±0.40	0.82±0.31	0.90±0.31	1.37±0.30	1.40±0.16		
pre U2 A	1.00±0.03	4.41±0.21	1.04±0.15	1.00±0.11	2.252±0.11	1.64±0.11	1.06±0.16	1.27±0.20	0.96±0.31	1.04±0.02		
pre U2 B	1.00±0.04	1.36±0.11	1.17±0.10	1.12±0.13	2.00±0.09	2.01±0.06	1.49±0.16	0.87±0.35	1.19±0.05	0.69±0.07		
pre U4 A	1.00±0.14	3.01±0.61	1.60±0.33	0.93±0.12	0.35±0.03	1.52±0.67	1.12±0.47	0.90±0.14	1..28±0.57	1.43±0.29		
pre U4 B	1.00±0.08	2.40±0.55	0.60±0.04	1.50±0.41	0.54±0.10	1.20±0.38	1.00±0.02	0.95±0.28	1.31±0.18	0.80±0.24		
pre U4 C	1.00±0.12	1.16±0.23	1.07±0.05	1.21±0.40	0.42±0.12	0.95±0.07	1.44±0.67	0.74±0.03	0.94±0.03	0.91±0.03		
pre U5 A	1.00±0.05	4.79±0.38	0.70±0.06	0.75±0.11	1.90±0.30	0.90±0.01	1.64±0.68	1.42±0.05	0.77±0.21	0.81±0.20		
pre U5 B	1.00±0.04	0.90±0.22	1.23±0.35	1.60±0.05	2.36±0.16	1.34±0.26	0.94±0.21	1.30±0.01	0.82±0.05	0.92±0.20		
pre U6	1.00±0.11	0.98±0.15	1.19±0.51	0.84±0.15	0.41±0.06	1.06±0.27	1.12±0.29	0.80±0.14	1.12±0.57	1.07±0.20		
pre snR10	1.00±0.13	1.11±0.44	1.24±0.36	1.31±0.23	0.51±0.06	0.73±0.11	0.91±0.03	0.73±0.09	1.89±0.13	1.51±0.12		
pre snR13 B	1.00±0.11	1.54±0.49	1.02±0.38	1.05±0.05	0.42±0.05	1.01±0.19	0.97±0.29	1.02±0.42	1.08±0.33	1.34±0.57		
presnR13 C	1.00±0.08	1.46±0.21	0.84±0.26	1.05±0.05	0.34±0.06	1.37±0.12	1.16±0.28	1.04±0.06	1.48±0.37	1.30±0.50		

**Steady-State levels of small non-coding precursor RNAs as determined from Oligo-dT Primed cDNA**												
**Precursor RNA Amplicons**	**WT**	***rrp6*-Δ**	***rrp4-1***	***GAL10::R RP41***	***trf4*-Δ**	***trf5*-Δ**	***air1*-Δ**	***cbc-1*Δ**				
pre 5S	1.02±0.25	0.79±0.48	0.93±0.19	0.62±0.08	1.68±0.39	5.12±0.43	3.04±0.50	0.80±0.39				
pre 5.8S C	1.00±0.12	3.96±0.50	2.09±0.53	0.53±0.09	0.76±0.19	1.56±0.07	1.22±0.19	1.46±0.01				
pre 5.8S D	1.00±0.10	1.18±0.16	2.65±0.22	0.50±0.10	0.98±0.25	1.76±0.18	1.09±0.08	1.24±0.34				
pre 5.8S E	0.96±0.18	2.02±0.14	ND	ND	ND	ND	ND	ND				
pre U1 C	1.00±0.07	2.44±0.49	1.21±0.10	0.70±0.10	2.8±0.34	1.21±0.51	0.58±0.13	0.88±0.21				
pre U1 D	1.00±0.07	0.98±0.17	1.08±0.43	1.32±0.66	2.29±0.29	1.18±0.54	1.11±0.37	1.00±0.30				
pre U1 E	1.00±0.02	0.95±0.03	0.97±0.28	1.17±0.51	2.62±0.19	1.07±0.26	0.89±0.14	0.97±0.18				
pre U2 A	1.00±0.09	3.53±0.66	1.26±0.08	0.81±0.05	2.07±0.20	1.15±0.14	0.91±0.03	1.30±0.28				
pre U2 B	1.00±0.06	1.37±0.01	1.75±0.07	0.64±0.15	0.85±0.00	1.41±0.09	0.98±0.08	1.12±0.01				
pre U4 A	1.00±0.10	2.32±0.23	0.96±0.64	0.83±0.14	2.55±0.20	1.37±0.61	1.13±0.16	1.28±0.16				
pre U4 B	1.00±0.09	1.26±0.53	1.16±0.50	1.14±0.30	2.37±0.30	1.07±0.40	0.82±0.27	1.02±0.19				
pre U4 C	1.00±0.09	1.12±0.06	1.08±0.22	0.93±0.16	2.03±0.18	0.77±0.29	0.78±0.16	0.94±0.27				
pre U5 A	1.00±0.03	3.45±0.31	1.12±0.03	0.79±0.06	1.85±0.03	0.75±0.10	0.82±0.04	0.93±0.00				
pre U5 B	1.00±0.07	0.95±0.01	1.03±0.04	1.67±0.09	1.77±0.12	0.96±0.03	0.98±0.09	1.22±0.10				
pre U6	1.00±0.12	0.98±0.43	1.28±0.26	1.31±0.31	2.14±0.57	1.07±0.37	0.95±0.24	1.17±0.39				
pre snR10	1.00±0.17	1.23±0.48	1.25±0.24	1.51±0.46	2.29±0.22	1.25±0.19	1.04±0.25	1.13±0.25				
pre snR13 B	1.00±0.02	2.21±0.59	1.29±0.23	1.33±0.13	2.19±0.31	1.15±0.16	0.85±0.12	0.92±0.20				
pre snR13 C	1.00±0.00	0.66±0.10	1.06±0.46	1.26±0.42	2.06±0.30	1.39±0.39	1.22±0.41	0.92±0.50				

ND - not determined.

### Enhancement of the steady-state levels of the polyadenylated forms of sncRNAs is associated with their diminished decay rates in the *rrp6*-Δ strain

Next, we determined the decay rates of 5S and 5.8S rRNAs in the WT and *rrp6*-Δ strain by shutting off the RNA polymerase I, II, and III transcriptions in separate experiments either by RT-qPCR assay (Fig. S4A-D) or by northern blot analysis at 30°C (Fig. S4E and F). In the RT-qPCR assay, we used random hexamer-primed cDNA with primer-sets/amplicons either corresponding to their mature regions or spanning the mature-to-3′-extended precursor regions. In the northern blot analysis, we used oligonucleotide probes corresponding to their mature regions. RT-qPCR analyses using their mature amplicons revealed that both 5S and 5.8S rRNAs displayed significantly diminished decay rates in the *rrp6*-Δ strain relative to the WT strain with a concomitant increase in their half-life values (107 and 190 minutes for 5S and 99 and 395 minutes for mature 5.8S in WT and *rrp6*-Δ strains, respectively; **Table 3**, Fig. S4A and B). Consistent with this finding, the northern blot analyses showed that the mature species of both the 5S and 5.8S rRNAs indeed undergo rapid decay in the WT strain, which was significantly diminished in the *rrp6*-Δ strain (Figs. S4E and F). Notably, the decay rates of the precursor species of 5S rRNA determined by RT-qPCR using an amplicon corresponding to its extended region did not reveal any alteration of decay rate and half-life value in *rrp6*-Δ and WT strains (**Table 3**, Fig. S5B), which is consistent with their steady-state levels. In the case of 5.8S rRNA, analysis of the decay rates and half-life values of pre-5.8S rRNA-C (corresponding to 5.8S+30 species) and pre-5.8S rRNA-D (corresponding to 7S) indicated a ~2.5 fold (108 and 265 minutes in WT and *rrp6*-Δ strains, respectively) and 1.6-fold (180 and 298 minutes in WT and *rrp6*-Δ strains) enhancements respectively in the *rrp6*-Δ strain (**Table 3**, Fig. S5D-E). These data suggest that the increase in the steady-state levels of the of 5S and 5.8S rRNAs in the *rrp6*-Δ strain correlates well with their decay rates.

**Table 3.** Half-life values of polyadenylated mature and precursor forms of various rRNAs in WT [*RRP6*^+^) and *rrp6*-Δ yeast strains (in minutes).

ncRNAs	5S	pre 5S	5.8S	pre 5.8S I	pre 5.8S II	SNR10	pre snR10 A	SNR13	pre snR13 A	pre snR13 B
**WT**	107.4	374.14	98.9	108	179.93	29.38	376.57	33.19	342.66	362.12
***rrp6-Δ***	190.6	358.28	395.05	265.23	298.51	355.62	454.93	479.50	531.1	564.04

Similar analyses of the decay rates by RT-qPCR assays using amplicons corresponding to the mature/CDS and 3′-extended precursor sequences of all the snRNAs (Figs. S4C, S5F-S) and two snoRNA species, snR10 and snR13 (Figs. S4D, S6A-D) indicated that mature pools of both of these sncRNAs specifically undergo an Rrp6p-dependent decay (**Table 4**). The decay rates of the total population of these sncRNAs become substantially diminished in the *rrp6*-Δ strain with a concomitant increase in their half-life values (5-28 fold increase for various snRNAs and 12 to 14 fold enhancement for snoRNAs, snR10 and snR13 RNAs; Figs. S4, S5, S6 and **Table 3** and **4**). Collective data from all the experiments presented here are thus consistent with the conclusion that polyadenylated versions of mature 5S, 5.8S rRNAs, snRNAs and select snoRNAs are subject to an active nuclear degradation by Rrp6p.

**Table 4.** Half-life values of polyadenylated mature and precursor forms of various sn- and snoRNAs in WT [*RRP6*^+^) and *rrp6*-Δ yeast strains (in minutes).

ncRNAs	U1	Pre U1A	Pre U1B	U2	Pre U2A	Pre U2B	U4	Pre U4A	Pre U4B	U5	Pre U5A	Pre U5B	U6	Pre U6A
**WT**	14.71	225.1	360.5	18.05	239.48	307.17	85.28	214.71	323.27	25.17	273.91	267.82	101	340.61
**rrp6-Δ**	414.64	356.9	372.9	236.1	315.05	429.06	352.66	334.48	428.02	232.75	241.99	253.39	479.58	447.7

### Nuclear decay of mature polyadenylated forms of sncRNAs requires Rrp47p along with Rrp6p

Since Rrp6p interacts with Rrp47p independent of the core nuclear exosome *in vitro* [69] and *in vivo* [70], we explored if Rrp6p-dependent degradation of the sncRNAs requires Rrp47p and Mpp6p.[70]. Consequently, we determined the levels of these RNA species in yeast strains carrying a deletion in *RRP47* and *MPP6* by RT-qPCR assay by using both random-primed and oligo-dT-primed cDNA samples from these strains using the amplicons spanning the mature/CDS regions. Remarkably, the polyadenylated forms of all of these ncRNAs, 5S, 5.8S, all snRNAs, snR10 and snR13 displayed an enhancement in their steady-state levels in *rrp6*-Δ *rrp47*-Δ, and *rrp6*-Δ *rrp47*-Δ double mutant yeast strains but not in the *mpp6*-Δ strain (**Fig. 1B**, Fig. S7A-E). The data therefore suggest that while Rrp47p participates in this nuclear decay, Mpp6p is not involved. The necessity of the catalytic/exonuclease activity of Rrp6p for this nuclear decay is demonstrated by a dramatic enhancement (~20-150 fold) of their polyadenylated version in a yeast strain carrying the *rrp6-3* allele harboring a catalytically inactive point mutation (D238A) in the exonuclease I domain of Rrp6p [71] (**Fig. 1B**, Fig. S7A-E). Remarkably, the extent of enhancement of the steady-state levels of polyadenylated forms of all these ncRNAs in the double mutant *rrp6*-Δ *rrp47*-Δ strain is no better than the levels found in each of the *rrp6*-Δand *rrp47*-Δ single mutant strains (**Fig. 1B**, Fig. S7A-E). This data, thus, exhibit a genetic epistasis between *RRP6* and *RRP47* genes concerning the nuclear decay of these ncRNAs. This finding was further confirmed by the steady-state levels of 5S, 5.8S, U1 and snR10 RNAs in WT, *rrp6*-Δ and *rrp47*-Δ strains determined by northern blot analysis, which reveals that indeed the levels of their mature forms increased significantly in *rrp6*-Δ and *rrp47*-Δ strains (**Fig. 1C**). Estimation of the relevant signals/bands associated with mature 5S and 5.8S, U1 and snR10 RNAs in the WT, revealed that their levels undergo approximately 3 to 5 fold in *rrp6*-Δ and *rrp47*-Δ strains, relative to the WT strain (**Fig. 1D**). Notably, levels of their enhancements estimated from northern blot analyses are in good agreement with their fold enhancement in these strains as determined from RT-qPCR analysis (**Fig. 1B**). Collectively, all these data support the idea that both Rrp6p and Rrp47p are acting together in this nuclear decay pathway to target and degrade the mature and polyadenylated sncRNAs.

### Genome-wide analysis of ncRNAs reveals a relatively higher accumulation of transcripts corresponding to the mature regions of the selected snoRNA genes in the *rrp6*-Δ strain

**Figure 2.**
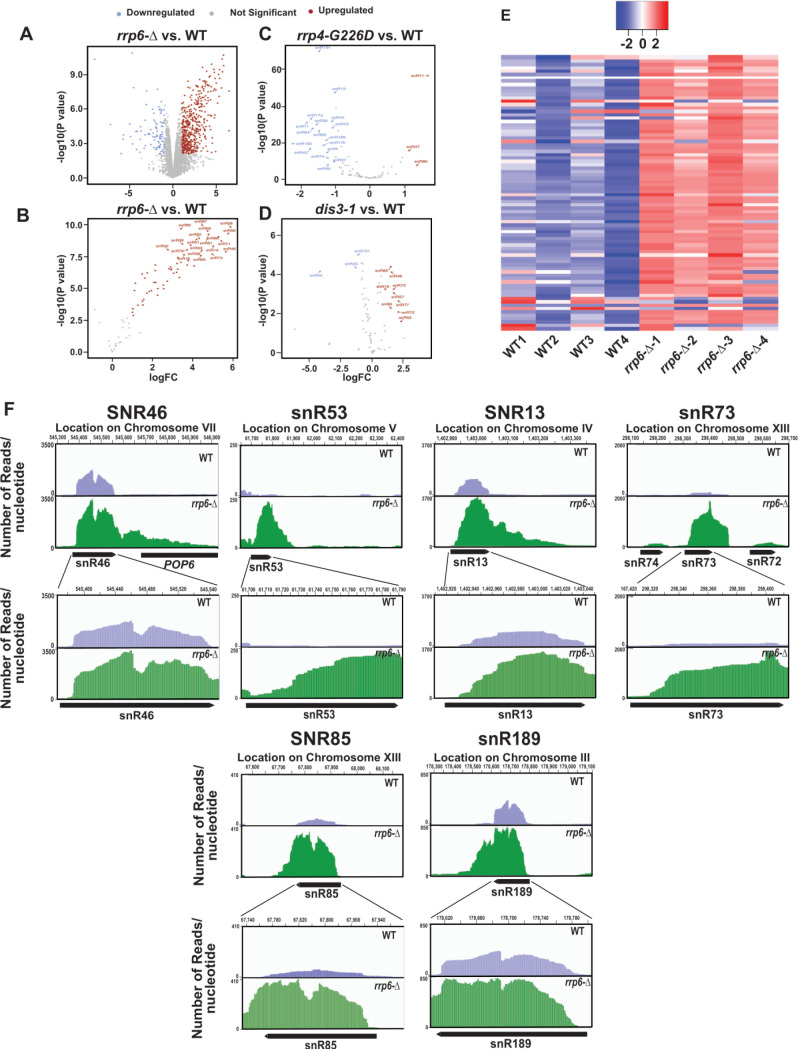
FIGURE 2: Analysis of previously published RNA-seq datasets (Accession Numbers, GSE135056, GSE163106, and GSE134295) revealed a dramatic accumulation of reads corresponding to the mature region of several small nucleolar RNAs. Volcano plots depicting the differential expression of all annotated sequences in *rrp6*-Δ **(A)**, and all 82 sn- and snoRNA transcripts in *rrp6*-Δ **(B)**, mutant *rrp4-G226D* **(C)**, and mutant *dis3-1* **(D)** yeast strain relative to WT strain. **(E)** Heat Map of normalized counts, showing the expression pattern for all 82 sn- and snoRNAs across four independent biological replicates of WT and *rrp6*-Δ yeast strains. **(F)** Graphical representation showing the relative amount of reads mapped to the genomic locus corresponding to small nucleolar RNAs snR46, snR53, snR13, snR73, snR71, snR685, and snR7189. The top panels depict the distant view (accommodating the 3′ extended regions), and the bottom panels show the close-up views for each snoRNA loci. The location of transcripts and the direction of transcription are shown below the graph (drawn in scale) by the solid black arrow-headed rectangles.

In the experiments described above we evaluated the steady-state levels of only two snoRNAs in WT and *rrp6*-Δ strains, as exemplified by snR10 (representatives of H/ACA box snoRNAs) and snR13 (representative of C/D box snoRNAs) out of the total explore if other snoRNAs display any significant enhancement in the *rrp6*-Δ strain, we reanalyzed three previously published RNA-Seq databases (accession number GSE135056 for *rrp6*-Δ vs. WT, accession number GSE163106 for *rrp4-G226D* vs. WT, and accession number GSE134295 for *dis3-1* vs. WT) [73–75]. We performed a differential expression analysis using EdgeR [76–78] in the WT and *rrp6*-Δ, *rrp4-G226D* and *dis3-1* mutant strains. The annotations used for our study included 82 snoRNAs. Interestingly, in the *rrp6*-Δ strain, the majority of the 82 snoRNA genes were found to undergo a significant upregulation ranging from 3 to 64 fold (LogFC change cutoffs = +/- 1, p-value cutoff = 0.05, **Fig. 2A-B**). In contrast, only three snoRNAs in *rrp4-G226D* and nine snoRNAs in *dis3-1* strains displayed only a marginal upregulation (approximately 2-5 fold) in strains carrying point mutations in the core exosome components Rrp4 and Dis3p (**Fig. 2C-D**). Moreover, a large number of snoRNAs in *rrp4-G226D* and a few of them in *dis3-1* strains were down-regulated quite significantly (**Fig. 2C-D**). To identify the region of snoRNA genes displaying accumulation of the maximum sequence reads in the *rrp6*-Δ strain, the BAM coverage plots portraying the number of reads aligned per base pair for six different snoRNAs across their genomic loci and their 3′-extended regions were evaluated (**Figs. 2F** and S7F). They include snR46, snR53, snR13, snR73, snR85, and snR189 and snR14/U4 in WT and *rrp6*-Δ yeast strains (**Figs. 2F** and S7F). These data suggest that although a considerable accumulation of reads were found across the 3′-extended regions of all these representative snoRNAs in the *rrp6*-Δ strain, the abundance of their 3′-extended reads relative to the mature reads is relatively less (**Fig. 2F**). The relative abundance profile of RNA-seq. reads of tested snoRNA genes in the CDS and 3′-extended regions indicate a similar trend as obtained from our RT-qPCR analysis (data not shown). These observations suggest that in the *rrp6*-Δ strain, the accumulated RNA-Seq reads in their mature region appear to be present in relatively higher amounts than that found in their 3′-extended regions. Thus, reanalysis of previous RNA-Seq datasets supports the idea that mature forms (in addition to their precursor forms) of several snoRNAs accumulate in the *rrp6*-Δ strain.

### Rrp6p and Rrp47p form a separate protein complex independent of the nuclear exosome that cross-links specifically to the polyadenylated version of mature length sncRNAs

**Figure 3.**
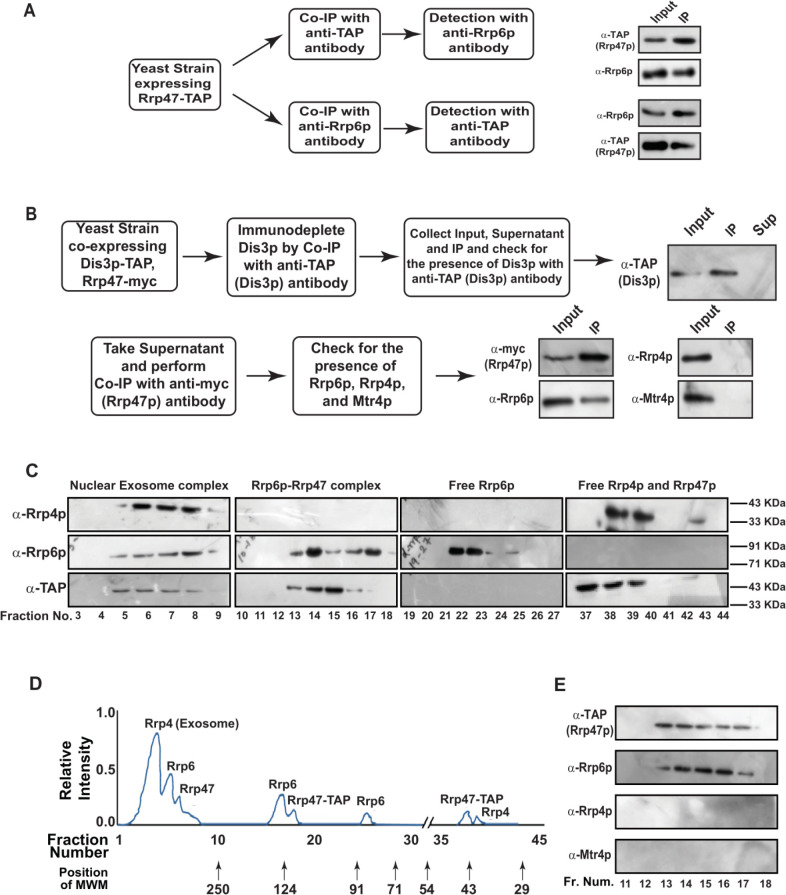
FIGURE 3: Rrp6p and Rrp47p exist as separate complex independent of the core exosome. **(A)** Workflow depicting the experimental approach involving the reciprocal Co-IP that demonstrates a physical association between Rrp6p and Rrp47p. Cell extracts prepared from the yeast cells (yBD-507) expressing Rrp47-TAP were subjected to immunoprecipitation either with anti-TAP antibody or with anti-Rrp6p antibody followed by detection of the Rrp6p in anti-TAP IP and detection of Rrp47p in anti-Rrp6p-IP. **(B)** Experimental outline to demonstrate the existence of Rrp6p-Rrp47p complex in the cell extract immune-depleted of Dis3p. Cell extracts prepared from the yeast strain expressing Dis3p-TAP and Rrp47-myc (yBD-540) was subjected to immunoprecipitate with anti-TAP antibody followed by detection of Dis3p in the input, IP, and supernatant fraction using an anti-TAP antibody. After ensuring the absence of Dis3p in the supernatant fraction (right side panel in the top row), it was further subjected to immunoprecipitation with an anti-myc antibody to precipitate Rrp47 followed by the detection for the presence of Rrp6p, Rrp4p (a component of the core exosome) and Mtr4p (a component of the TRAMP complex). The methodology involving cell extract preparation, immunoprecipitation, and western blotting is described in the materials and method section. **(C-D)** Fractionation/separation profiles of the components of the nuclear exosome, Rrp6/47p and free Rrp6p, Rrp4p, and Rrp47p in Biogel P-200 gel-filtration chromatographic column showing Rrp6p and Rrp47p exists as a separate complex independent of the core nuclear exosome. Cell extracts prepared from the yeast strain expressing Rrp47-TAP (yBD-507) was subjected to fractionation by Biogel P-200 Gel filtration column as described in materials and methods and the eluted fractions were subjected to western blotting analysis using anti-Rrp4p, anti-Rrp6p, and anti-TAP (for detection of Rrp47p). Panel D depicts a qualitative representation of the elution profile of different proteins in the Biogel P-200 Gel filtration column. Relative elution volumes of various MW markers eluted from this column and the fractions in which they appeared are indicated in this profile. **(E)** Fraction numbers 11 to 18 of the above elution from the Biogel P-200 column were further subjected to separate co-immunoprecipitation by anti-TAP Ab, and further checked with either anti-TAP (for detection of Rrp47p), or anti-Rrp4p and anti-Mtr4p antibody to demonstrate the absence of the core nuclear exosome and the TRAMP complex in those eluates.

To explore if Rrp6/47p-dependent decay of mature length polyadenylated forms of ncRNA is independent of the nuclear exosome activity, we addressed if (i) Rrp6p and Rrp47p constitute a separate and core nuclear exosome-independent protein complex and (ii) the mature length polyadenylated forms of the sncRNAs become enriched within the Rrp6/47p complex. Towards this, we first verified if Rrp6p and Rrp47p interact with each other *in vivo* by performing reciprocal Co-IP experiments, in which the first Co-IP was carried out using an extract prepared from a WT yeast strain expressing Rrp47-TAP with anti-TAP (for Rrp47-TAP) antibody followed by the detection of Rrp6p using Rrp6p-specific antibody and *vice versa* (**Fig. 3A**). Strong signals of Rrp6p in the anti-TAP IP and Rrp47 in the anti-Rrp6p IP were detected, indicating a physical association between these two proteins (**Fig. 3A**). Next, we addressed if Rrp6/47p could be detected in a cell extract depleted of the core nuclear exosome. An extract prepared from a WT yeast strain expressing Dis3-TAP and Rrp47-myc was subjected to an extensive immune-depletion with the anti-TAP antibody to drain the Dis3-associated core nuclear exosome completely. Subsequent tests for the presence of Dis3p revealed its existence in the input and IP but not in the supernatant (**Fig. 3B**, top row). Successive Co-IP of the Dis3p-depleted supernatant with anti-*myc* (to pull down Rrp47p) and anti-Rrp6p antibodies demonstrated the presence of both Rrp47p and Rrp6p in the IP, which lacks Rrp4p and Mtr4p (**Fig. 3B**, bottom row). Data from these experiments strongly indicated that Rrp6 and Rrp47 interact with each other in a core-exosome-independent manner and may form an independent complex *in vivo*.

To further bolster the exosome-free existence of the Rrp6p/47p complex, we subjected the cell extract prepared from yeast strain expressing Rrp47p-TAP to a Biogel P-200 Gel filtration column chromatography. This procedure was then followed by the detection of Rrp4p (representative of the core exosome, Exo-11), Rrp6p (representative of the core exosome and a putative Rrp6/47 complex), and Rrp47p (representative of the core exosome, and a putative Rrp6/47 complex) in the eluted fractions by western blotting analysis. As shown in **Fig. 3C-D**, analysis of the fractionation profiles of these three representative proteins suggests that Rrp4p, Rrp6p, and Rrp47p are detectable in four different sets of fractions: 3-9 (Rrp4p, Rrp6p, and Rrp47p detected together), 13-18 (Rrp6p and Rrp47p detected together), 22-25 (only Rrp6p) and 37-40 (Rrp47 and Rrp4p). Notably, the elution volumes at which these proteins/complexes were eluted are consistent with that of the known molecular weight markers (**Fig. 3D**). These data support the view that the nuclear exosome Exo-11 (MW ~480 kDa), being a large protein complex, is eluted in the early fractions (in void volume) 3-9, followed by the elution of Rrp6p/Rrp47p-TAP complex (MW ~125 kDa) in fractions 13-18. The free forms of each of these proteins were detected in the later fractions, which is consistent with their molecular sizes/weights (Rrp6p=84 KDa., Rrp47p-TAP=42 kDa., and Rrp4=39.5 kDa.). To affirm the absence of the core-nuclear exosome and the TRAMP components in fractions 13-18 containing Rrp6 and Rrp47p, we further confirmed the absence of Rrp4p and Mtr4p in these fractions by subjecting them to western blot analysis using their specific antibodies (**Fig. 3E**). Thus, our data strongly support the model that Rrp6p co-purifies with Rrp47p as a separate complex from the core-nuclear exosome (Exo-11) in the Biogel P-200 gel exclusion column (**Fig. 3C-E**), thereby substantiating that Rrp6p/47p exists as an exosome-independent complex.

**Figure 4.**
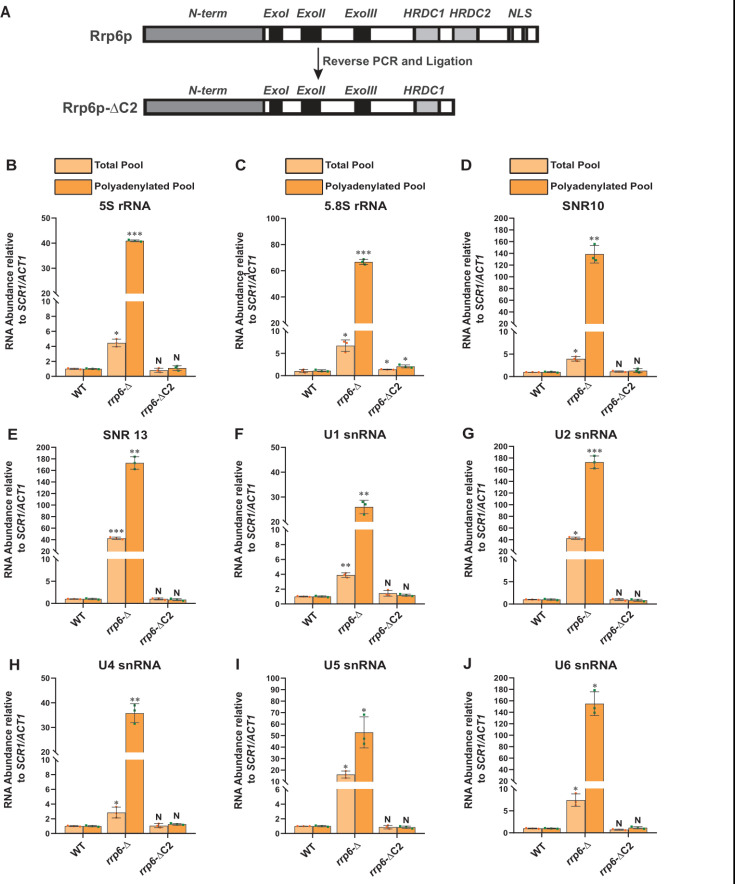
FIGURE 4: A truncated version of Rrp6p consisting of a deletion of the C-terminal domain that abolishes its interaction with the core exosome is equally competent in maintaining the steady-state levels of the polyadenylated version of mature populations of all sncRNAs like its native full-length counterpart. **(A)** Cartoon showing the various functional domains of native full-length Rrp6p and Rrp6p-ΔC2 lacking its entire C-terminal domain. **(B-J)** Scattered/Bar plots revealing the steady-state levels of various low molecular weight ncRNAs estimated from the 2 ng cDNA samples prepared using random hexanucleotide primers (pale yellow histograms) or oligo-dT_30_ anchor primer (dark yellow histograms) by RT-qPCR from the WT strain (yBD-117), yeast strains carrying either a complete deletion of *RRP6* (*rrp6*-Δ; yBD-129) or a deletion of its C-terminal domain (*rrp6*-ΔC2: yBD-527)*. SCR1* (in the case of RT-qPCR assays using random primer) and *ACT1* mRNA (in the case of RT-qPCR assays using oligo dT_30_ primer) were used as the internal loading control. Normalized values of each of the ncRNAs in the WT yeast strain were set to 1. Three independent cDNA preparations (biological replicates, n = 3) were used to determine the levels of various ncRNAs. The statistical significance of difference reflected in the ranges of P values estimated from Student's two-tailed t-tests for a given pair of test strains for every message are presented with the following symbols, *<0.05, **<0.005, and ***<0.001; N, not significant.

To validate that the exosome-independent Rrp6p/47p complex coordinates the nuclear degradation of the sncRNAs, we created a C-terminally truncated version of Rrp6p (dubbed Rrp6-ΔC2) using a reverse PCR technology as described in materials and methods. This truncated version lacks the entire C-terminal region (*HRDC2* and *NLS* domains) responsible for making contact with the core nuclear exosome [47] (**Fig 4A**). Subsequently, we addressed if the physical association of Rrp6p with the exosome is necessary for carrying out the nuclear decay of these polyadenylated sncRNAs. As shown in **Fig. 4B-J**, unlike the *rrp6*-Δ strain, the steady-state-levels of both total as well as polyadenylated pools of all the representative sncRNAs remained very similar in the WT strain and in the strain expressing C-terminally truncated Rrp6p-ΔC2 version. Notably, the full-length *RRP6*^+^ construct and its C-terminally truncated version (ΔC2) were constructed by cloning the corresponding genomic DNA copy and its truncated version into a yeast *CEN* plasmid. Since the *RRP6*^+^ gene is located very close to the *CEN15* centromere, it is conceivable that these recombinant plasmids carrying the full-length *RRP6*^+^ and its ΔC2 derivative may have two centromeres, which potentially may lead to an alteration of expression of Rrp6p from these constructs. To rule this possibility out that comparable efficiency of RNA decay activity of Rrp6-ΔC2 protein (with that of native Rrp6p) results from the phenotypic variability in its expression level, we determined the levels of Rrp6p in a WT strain with a genomic copy of *RRP6*^+^, an *rrp6*-Δ strain, and the strains harboring *CEN* plasmids expressing either *RRP6*^+^ or Rrp6-ΔC2 allele. As shown in Fig S7G, the level of expression of Rrp6p/Rrp6-ΔC2 from the native genomic copy, and two *CEN* plasmids are very similar and comparable. This finding, thus, supports the idea that the ability of the Rrp6-ΔC2 protein lacking its C-terminal domain to support the decay of the sncRNAs is not associated with its altered expression level. These data strongly suggest that Rrp6p-ΔC2, lacking its association with the core exosome, is fully competent to support the nuclear decay of all the representative sncRNAs just like its full-length native counterpart, thereby demonstrating that the physical association between the exosome and Rrp6p is dispensable for this function.

**Figure 5.**
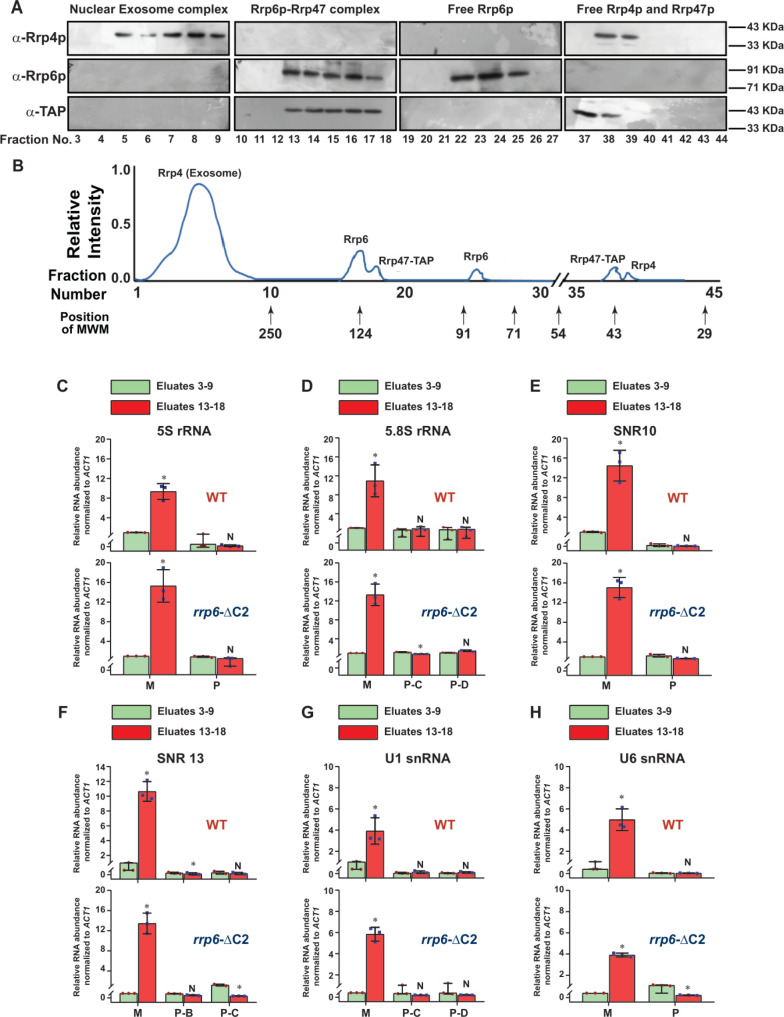
FIGURE 5: Rrp6p ΔC2 and Rrp47p co-purify as a separate independent protein complex that cross-links to the entire population of mature/processed species of sncRNAs. **(A)** Fractionation/separation profiles of the components of the nuclear exosome, Rrp6-ΔC2/47p and free Rrp6p, Rrp4p, and Rrp47p in Biogel P-200 gel-filtration chromatographic column showing Rrp6p-ΔC2-Rrp47p exists as a separate complex independent of the core nuclear exosome. Growing cultures of yeast strains expressing Rrp6-ΔC2 and Rrp47-TAP (yBD-527) were subjected to UV irradiation for inducing RNA-protein cross-linking before harvesting as described in the materials and methods. Cell extract was subsequently prepared from the harvested cells that were further subjected to the fractionation by Biogel P-200 Gel filtration column as described in materials and methods and the eluted fractions were subjected to western blotting analysis using anti-Rrp4p, anti-Rrp6p, and anti-TAP (for detection of Rrp47p). Panel **(B)** depicts a qualitative representation of the elution profile of different proteins in the Biogel P-200 Gel filtration column. Note that in this experiment no Rrp6p/47p signal was detected in the fraction numbers 3-9 when they were either probed with anti-Rrp6p or anti-TAP antibodies showing a complete lack of interaction/association with the nuclear exosome. Relative elution volumes of various MW markers eluted from this column and the fractions in which they appeared are indicated in this profile. **(C-H)** Polyadenylated forms of all mature sncRNAs are enriched in the fractions that contain the Rrp6-ΔC2p-Rrp47p complex. Scattered/Bar plots revealing the distribution of the total (mature + precursor forms together) population and different precursor species of various low molecular weight ncRNAs in the pooled fractions 5-9 (corresponding to the position of the core nuclear exosome, green histograms) and 13-18 (corresponding to the position of the Rrp6/47p complex, orange histograms). Extracts were prepared from growing cultures of WT (yBD-507) and *rrp6*-ΔC2 (yBD-527) strains that were subjected to UVcrosslinking before harvesting. These cross-linked cell extracts were subjected to fractionation in the Biogel P-200 gel filtration column as before. Fraction numbers, 5-9 and 13-18 were subsequently pooled and subjected to RNA isolation and cDNA synthesis using oligo-dT_30_ anchor primer followed by RT-qPCR assay with 2 ng of cDNA using amplicons corresponding to CDS and 3′-extended precursor regions of various sncRNAs. *ACT1* mRNA was used as the internal loading control. Normalized values of the total population of each ncRNA in both WT and *rrp6*-ΔC2 strains obtained from the pooled fractions 5-9 were set to 1. Three independent cDNA preparations (biological replicates, n = 3) were used to determine the levels of various ncRNAs. The statistical significance of difference reflected in the ranges of P values estimated from Student's two-tailed t-tests for a given pair of test strains for every message are presented with the following symbols, *<0.05, **<0.005, and ***<0.001; N, not significant.

Next, we verified whether the truncated construct we created indeed displays a lack of association with the core exosome and if the polyadenylated forms of the sncRNAs can interact with the exosome-free Rrp6/47 complex. For this experiment, growing cultures of yeast strains expressing Rrp6p-ΔC2 and Rrp47p-TAP were subjected to UV-irradiation (to promote RNA-protein cross-linking) before harvesting. The cell extract was subsequently prepared and was subjected to fractionation in a Biogel P-200 column chromatography followed by elution of different complexes and the detection of Rrp4p, Rrp6p, and Rrp6/47 (using anti-TAP antibody) in the eluted fractions by western blotting analysis. As shown in **Fig. 5A-B**, the fractionation profiles of these proteins suggest that while Rrp4p is detectable in fractions 3-9 (characteristic profile of the core nuclear exosome), none of the Rrp6p and Rrp47p could be detectable in these fractions (**Fig. 5A**). In contrast, both of these proteins were detected in fractions 13-18, which is the characteristic volume of elution of the exosome-free Rrp6p/47p complex (consult **Fig. 3C** for a comparison). As usual, the free forms of these proteins were detected in the later fractions, Rrp6p-ΔC2 in fractions 22-25 and Rrp4p and Rrp47p in fractions 37-40. This data, therefore, strongly suggest that owing to the lack of the C-terminal domain, the Rrp6p-ΔC2 protein lost its association with the core nuclear exosome, yet it retains the ability to support the nuclear decay of sncRNAs.

To further reinforce the notion that the nuclear degradation of the mature length ncRNAs relies explicitly on the Rrp6p/47p complex and does not require the functional involvement of the core nuclear exosome, we address if the polyadenylatedmature length sncRNAs display a physical association with the exosome-free Rrp6p/47p complex. Initially, we carried out a RIP experiment from WT, *rrp6*-Δ and *rrp6*-ΔC2 strains using anti-Rrp6p antibody coupled to the extraction of RNA from Rrp6p-IP and preparation of oligo-dT primed cDNA to verify if the sncRNAs at all physically interact with Rrp6p. As shown in supplementary Fig. S7H, the abundance/levels of mature/precursor 5S, 5.8S, U1 and snR10 RNAs recovered from Rrp6p-IP from WT and *rrp6*-ΔC2 strains are significantly higher relative to their abundance found in the *rrp6*-Δ strain thereby affirming that their mature forms display a reasonably strong interaction with both the full-length Rrp6p and its C-terminally truncated version. Furthermore, the abundance of all of these RNAs in Rrp6-IP from the WT (expressing full-length Rrp6p) strain was found to be 3-5 fold higher relative to the levels of their precursor forms in the same IP (supplementary Fig. S7H). This data demonstrate that mature polyadenylated forms of these ncRNAs preferentially bind to the cellular pool of Rrp6p/Rrp6-ΔC2p that does not associate with the core exosome demonstrating a strong physical association between these sncRNAs and the exosome-free full-length Rrp6p and Rrp6-ΔC2p.

Since anti-Rrp6p precipitates both the exosome-associated and free Rrp6p together from WT cell extract in the RIP assay, we further explored the physical association between the polyadenylated forms of these RNAs with the exosome-free Rrp6p/47p complex. Subsequently, UV-cross-linked cell extracts were prepared from yeast strains expressing full-length (FL) Rrp6p and Rrp6p-ΔC2 and were subjected to the fractionation in Biogel P200 gel-filtration column. Following elution, fractions 3-9 and 13-18 were pooled separately from each sample, followed by the estimation of mature (M) and precursor (P) forms of each of these ncRNAs using RT-qPCR assay with primer-sets corresponding to the CDS and the 3′-extended downstream regions. A comparison of the abundance of their mature (M) and precursor (P) forms that were enriched in various fractions revealed that the mature forms of all the ncRNAs were selectively cross-linked 8-12 times more strongly to the pooled elutes corresponding to fractions 13-18 relative to the pooled elutes corresponding to fractions 3-9 (**Fig. 5C-H**). The relative enrichment of the precursor forms of these RNAs in eluates 13-18 fractions was highly insignificant compared to their mature-length forms (**Fig. 5C-H**). The selective enrichment of mature-length ncRNA species to Rrp6/47p complex thus firmly established that the mature polyadenylated forms (and not their plausible precursor forms) of the sncRNAs are targeted and degraded by the Rrp6p-Rrp47p complex, independent of the core nuclear exosome (see **Fig. 8**).

### Both the canonical and non-canonical poly(A) polymerases Pap1p and Trf4p catalyze the polyadenylation of sncRNAs

**Figure 6.**
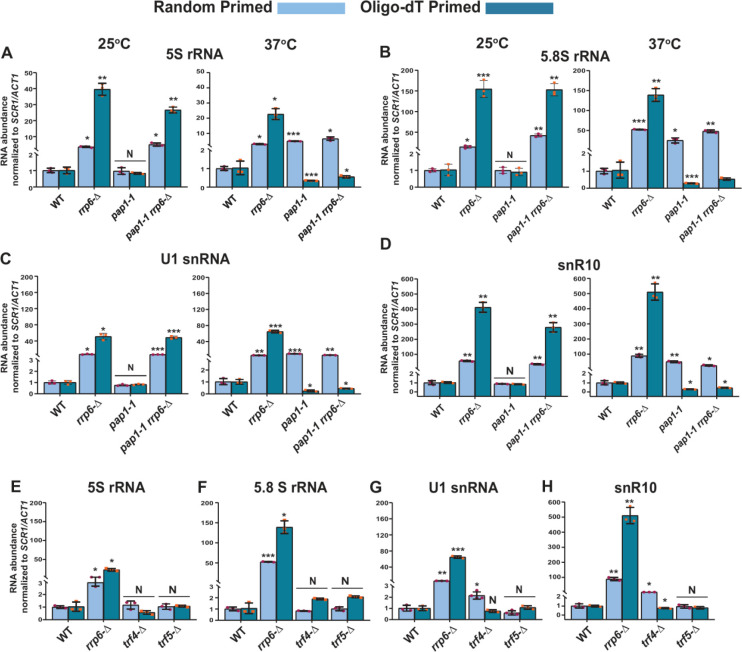
FIGURE 6: Both the canonical Poly(A) polymerase Pap1p and non-canonical Poly(A) polymerase Trf4p play a vital role in the polyadenylation of the sncRNAs. Scattered/Bar plot revealing the steady-state levels of 5S, 5.8S, U1, and snR10 RNAs estimated from the 2 ng cDNA samples prepared using random hexanucleotide primers (sky blue bars) or oligo-dT_30_ anchor primer (indigo blue bars) by RT-qPCR from WT strain (yBD-161) and strains carrying mutations in the *RRP6* (*rrp6*-Δ, yBD-162), *PAP1* (*pap1-1*, yBD-163), *RRP6* and *PAP1* (*pap1-1 rrp6*-Δ, yBD-179) (panels A-D) or WT (yBD-263), *RRP6* (*rrp6*-Δ) (yBD-265), *TRF4* (*trf4*-Δ) (yBD-306), *TRF5* (*trf5*-Δ) (yBD-334) genes (panels E-H). The *pap1-1* and *pap1-1 rrp6*-Δ strains were pre-grown at 25°C, followed by splitting the culture into two halves. Half of the culture continued to grow at 25°C for 2 hours and a 2-hour shift to 37°C were performed on the other half of the culture before harvesting them. Total RNA and cDNA isolation from them followed by RT-qPCR reaction carried out as described in materials and methods. *SCR1* (in the case of Random Primer) and *ACT1* mRNA (in the case of oligo dT_30_ primer) were used as the internal loading control. Normalized values of each of the ncRNAs in the WT yeast strain were set to 1. Three independent cDNA preparations (biological replicates, n = 3) were used to determine the levels of various ncRNAs. The statistical significance of difference reflected in the ranges of P values estimated from Student's two-tailed t-tests for a given pair of test strains for every message are presented with the following symbols, *<0.05, **<0.005, and ***<0.001; N, not significant.

Two different poly (A) polymerases are present in the genome of the baker's yeast *S. cerevisiae* – a canonical Pap1p, and a non-canonical Trf4/5p. The major canonical poly(A) polymerase Pap1p, an integral part of the cleavage/polyadenylation complex [79] catalyzes the template-independent addition of poly(A) tails in most mRNAs. We first addressed if Pap1p plays any role in the polyadenylation of these species of sncRNAs by determining the total and polyadenylated pools of the sncRNAs in a strain carrying a temperature-sensitive (ts) *pap1-1* allele [80]. A comparison of their steady-state levels in WT, *rrp6*-Δ, *pap1-1*, and *pap1-1* *rrp6*-Δ strains by RT-qPCR at the permissive and non-permissive temperatures of 25°C and 37°C unveiled that none of these sncRNAs displayed any enhancement in *pap1-1* strains at the permissive condition of 25°C (**Fig. 6A-D** and S8A-E, left histogram). Notably, the steady-state levels of these ncRNAs determined using only random-primed cDNA samples displayed an enhancement in the *pap1-1* and *pap1-1 rrp6*-Δ double mutant strains at the non-permissive condition of 37°C, which is similar and comparable to that observed in single *rrp6*-Δ strains (**Fig. 6A-D** and S8A-E, right histogram). Strikingly, the steady-state levels of all of the ncRNAs determined from oligo-dT_30_-primed cDNA samples were dramatically declined in strains carrying *pap1-1* and *pap1-1 rrp6*-Δ alleles at 37°C (**Fig. 6A-D** and S8A-E, right histogram). This data are consistent with the idea that at 37°C in the *pap1-1* strain, the global RNAs become unadenylated owing to the complete manifestation of the *pap1-1* phenotype [80]. Thus, oligo-dT primers failed to prime gross cDNA synthesis under this condition leading to a low RT-qPCR signal. Our finding, therefore, strongly favors the argument that Pap1p catalyzes the polyadenylation of these sncRNAs, making the mark for their degradation.

Notably, aberrant/faulty mRNAs and a few ncRNA substrates in the nucleus and nucleolus undergo short polyadenylation by the non-canonical Trf4/5 complex before being targeted for decay by the nuclear exosome [14, 39, 65]. As shown in **Figs. 1A** and S2A, our initial results suggest that the steady-state levels of the small non-coding 5S and 5.8S rRNAs, snRNAs, and selected snoRNAs display marginal augmentation in yeast strains carrying the *trf4*-Δ allele. A rigorous analysis of both the random-primed and oligo-dT-primed cDNA samples prepared from isogenic *trf4*-Δ and *trf5*-Δ strains displayed only a moderate accumulation of only snRNAs and selected snoRNAs in the *trf4*-Δ strain, which is not comparable to the level of their accumulation in the *rrp6*-Δ strain (**Figs. 6E-H** and S8F-J). Interestingly, no significant accumulation of 5S and 5.8S rRNA was noted either in *trf4*-Δ or *trf5*-Δ strains (**Figs. 6E-H** and S8F-J). Thus, these data suggest that while Pap1p carries out polyadenylation of the small rRNA species, both Pap1p and Trf4p catalyze other sncRNAs' polyadenylation (see discussion).

### Polyadenylation of sncRNAs occurs at the sites located at upstream and extended regions with respect to mature ends

**Figure 7.**
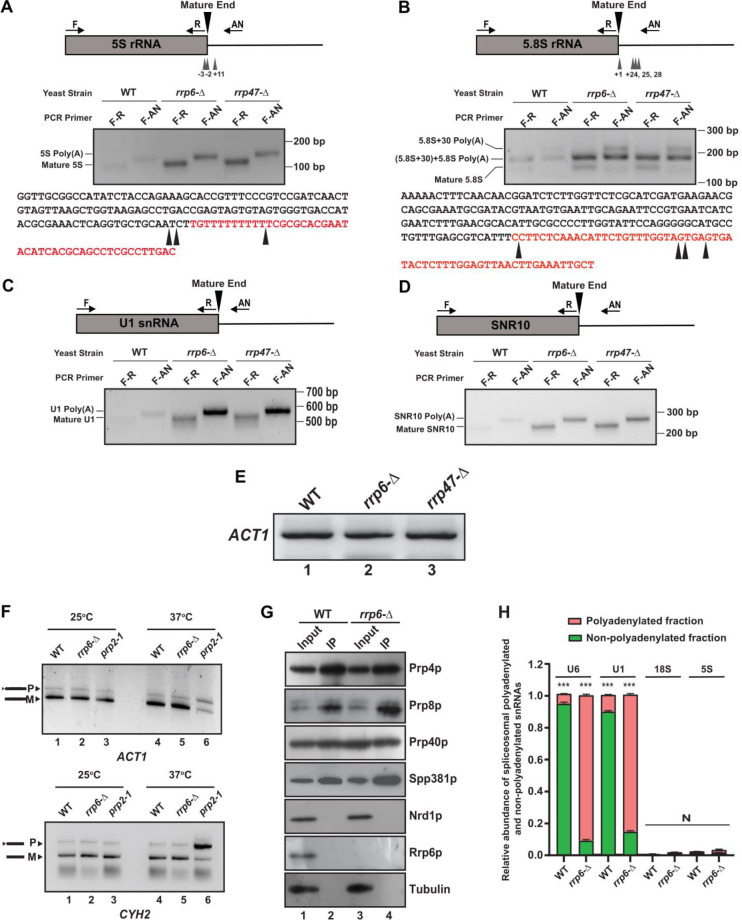
FIGURE 7: Polyadenylated versions of sncRNAs are accumulated in an *rrp6*-Δ mutant yeast strain. **(A-B)** Polyadenylation profiles of 5S **(A)**, and 5.8S **(B)**, RNAs. Each panel depicts a cartoon of the mature RNA along with the primer positions (F, R, and AN), the major polyadenylation sites. Gel images below the cartoon show the LM-PAT products of specific RNAs obtained with various primer sets at the bottom. The bottom parts of each panel show the genomic sequences of various ncRNAs with arrowheads indicating major polyadenylation sites identified. **(C-D)** Polyadenylation profiles of U1 **(C)**, and snR10 **(D)** RNAs showing the cartoon of the mature RNA with the primer positions (F, R, and AN) and gel images depicting their LM-PAT products obtained with various primer sets. LM-PAT cDNA samples of these RNAs were prepared from isogenic WT (yBD-263) and strains carrying *rrp6*-Δ (yBD-265)*,* and *rrp47*-Δ (yBD-454) yeast strains as described in materials and methods and then PCR amplified with either F-R (mature) and F-AN (polyadenylated) primer-sets specific for various RNAs followed by electrophoresis on a 2.5 – 3% agarose gel before photographed. **(E)** Relative levels of *ACT1* mRNA amplified using *ACT1* specific primer sets (see Table S3 for their sequence) from the LM-PAT cDNA prepared from WT (yBD-263), *rrp6*-Δ (yBD-265)*,* and *rrp47*-Δ (yBD-454) yeast strains as described in materials and methods. **(F)** Deletion in *RRP6*, *rrp6*-Δ does not impair the splicing of the *ACT1* and *CYH2* pre-mRNAs at 37°C. Relative steady-state levels of precursor and mature forms of *ACT1* and *CYH2* mRNAs at 25°C and 37°C temperature in the isogenic WT (yBD-122), *rrp6*-Δ (yBD-70), and and *prp2-1* (yBD-77) strains. Representative gel showing the relative levels of the precursor as well as mature *ACT1* and *CYH2* mRNA in these strains as determined by the semi-quantitative end-point PCR reactions carried out using the primer sets located within the exon1 and exon2 of each gene. Indicated strains were grown at 25°C till the OD of the culture reaches 0.6 followed by division of each culture into two halves. One half was continued to grow at 25°C and the other half was shifted to 37°C. Both cultures were continued to grow for 2 hour after the shift before harvesting. **(G)** Result from a representative co-immunoprecipitation experiment using hexa-HA-tagged Prp4p as bait to show purity of the spliceosomal prepration. Protein extracts from WT (yBD608) and *rrp6*-Δ (yBD614) cells expressing genomically hexa-HA-tagged version of Prp4p, were subjected to the immunopurification procedure. The immunoprecipitate (IP) was recovered, fractionated by electrophoresis on denaturing SDS gel followed by the detection of the specific spliceosomal and non-spliceosomal components using western blotting with specific antibodies as mentioned in Materials and Methods. The input (Lanes 1 and 3) in case of each sample is 100 µg of whole-cell protein extract from the respective strains, while the Co-IP (Lanes 2 and 4) for each lane represents 20 µl of the eluate in SDS-gel loading buffer from respective strains. **(H)** Relative abundance of polyadenylated and non-polyadenylated snRNAs in the immunopurified spliceosomal preparations. The immunoprecipitate (IP) from WT and *rrp6*-Δ yeast cells both expressing genomically tagged hexa-HA-tagged version of Prp4p was subjected to isolation of total RNA, followed by preparation of random-primed and oligo-dT primed cDNA and subsequent quantification of polyadenylated and non-polyadenylated fractions of U6/U1 snRNAs and 18S/5S rRNAs using 2 ng cDNA in each case. *ACT1* mRNA were used as the internal loading control. Normalized values of combined fractions of adenylated and non-adenylated snRNAs in each strain were set to 1. Three independent cDNA preparations (biological replicates, n = 3) were used. P values of the adenylated fractions were estimated in each case with respect to the non-adenylated fractions, which were considered as reference with the following symbols, *<0.05, **<0.005, and ***<0.001; N, not significant.

Accumulation of mature length polyadenylated species of sncRNAs in *rrp6*-Δ and *rrp47*-Δ strains deduced from RT-qPCR analysis using oligo-dT primed cDNA prompted us to explore the polyadenylation status of the select representative ncRNAs. We employed LM-PAT (**L**igation-**M**ediated **P**oly**A T**ail) assay (Fig. S9A) to precisely determine both the site(s) of the polyadenylation and the length of the poly(A) tail [81]. Using this technique, the LM-PAT cDNA samples were prepared from the WT, *rrp6*-Δ and *rrp47*-Δ strains followed by specific amplification of either mature region or mature region along with polyA tail and anchor together for a given ncRNA (Fig. S9A). Distinct LM-PAT PCR products of 5S, 5.8S, U1, and snR10 of lengths consistent with either their mature size alone (when amplified with mature region-specific primer F-R pair) or polyadenylated species (when amplified with mature sense and anchor primer F-AN pair) were observed specifically in *rrp6*-Δ and *rrp47*-Δ strains that were not detected in ‘no-RT’ control (data not shown). Notably, the abundance of these polyadenylated species in the WT strain is very low (barely detectable) relative to *rrp6*-Δ and *rrp47*-Δ strains. Interestingly, the sizes of the polyadenylated products of each ncRNA correspond to the combined lengths of their mature RNA, polyadenylated tail, and anchor were observed in each case that supports the idea that polyadenylated species of each ncRNA with either a mature or near-mature length accumulate in *rrp6*-Δand *rrp47*-Δstrains (**Fig. 7A-D**).

The polyadenylated LM-PAT products of 5S and 5.8S rRNAs obtained from the *rrp6*-Δ strain was subsequently cloned, and 20 randomly selected clones of each one were subjected to sequencing and the sequence data were mapped to the genomic sequences of these ncRNAs that revealed their sites of the polyadenylation and the lengths of the polyA tracts. Sequences analysis of the 20 randomly selected LM-PAT clones of 5S from *rrp6*-Δ strain revealed three major polyadenylation sites for 5S, at -3, -2, and +11 with respect to mature 3′-end (**Fig. 7A**, **Table 5**, Supplementary Table S8) and an average tract length of twelve to 87 adenylate residues (**Table 5**, Supplementary Table S8). Of these, three classes of observed 3′-endpoints of 5S rRNA transcripts, nearly 30% constitute +11 sites and rest 70% comprise -2 and -3 endpoints (Supplementary Table S8). Interestingly, the abundance of the 5S RNA species with longer than fifty adenylate residues constitute only 10% of the number of clones analysed (they are present in the transcripts with -3/-2 endpoints). For 5.8S rRNA, we sequenced 20 LM-PAT clones and the data unveiled two major classes of polyadenylation sites, one at +1 (represents mature 5.8S rRNA) and the other one is around +24, +25, and +28 (presumably of 5.8S+30 precursor) with respect to mature 3′-end at the nearly equal frequency (**Fig. 7B**). Of these 20 clones eight clones constitute +1 and twelve clones constitute +25/28 endpoints (Supplementary Table S8). The average lengths of poly(A) tails at these sites were found to be ten and 19 residues long respectively. Interestingly, the 5.8S transcripts with +1 endpoint seem to carry the poly(A) tract of ten to twelve residues whereas the transcripts with +25/28 endpoints harbour the longer poly(A) tract (16-19 adenylate residues; **Table 5**). Interestingly, the similar analysis in WT strain revealed that polyadenylated versions of 5S with -3 and -2 endpoints (with a tract of ten A residues) and 5.8S with +1 and +25 endpoints (with a tract of twelve A residues) rRNA species are detectable albeit at a much lower abundance (data not shown). These data thus suggest that perhaps aberrantly and incompletely processed 5S and 5.8S rRNA species are polyadenylated in the WT strain, which accumulate in the *rrp6*-Δ strain (**Fig. 7B**). For U1 and snR10 rRNAs, although, we did not analyze sequences of their LM-PAT clones, approximate estimations of their poly(A) tail-lengths from analysis of their gel images (**Fig. 7C-D**) are consistent with the lengths of ten to 15 residues respectively.

**Table 5.** Polyadenylation profiles of various sncRNAs revealed from this study.

SI No.	RNA	Major sites of Polyadenylation with respect to mature 3'-end	Average Length of Polyadenylated Tract (No. of AA residues)
1	5S rRNA	-2, -3, +11	12-14
2	5.8S rRNA	+1, +27	10-15
3	U1snRNA	ND	ND
4	SNR10 snoRNA	ND	ND

ND - not determined.

The above finding therefore strongly suggests that small rRNA species, which undergo inaccurate 3′-end maturation are subject to the rapid nuclear degradation by Rrp6p/47p independent of the core-nuclear exosome. It should be noted here that the abundance of the polyadenylated species of each ncRNA in *rrp6*-Δ and *rrp47*-Δ strains (including the 5.8S mature length species; **Fig. 7A-D**) are significantly higher relative to that obtained in the WT strain thereby mirroring the findings obtained by RT-qPCR and northern blot analysis. Importantly, none of these polyadenylated LM-PAT PCR products were detected from the *pap1-1* yeast strain at the non-permissive temperature of 37°C (data not shown). This finding also corroborates our previous observation that their levels determined by RT-qPCR assay from oligo-dT_30_-primed cDNA samples from the *pap1-1* strain dramatically dropped at the non-permissive temperature (**Fig. 6A-D**, 37°C, right histograms) thereby suggesting, that the canonical polymerase Pap1p plays a major role in their polyadenylation. All these data, collectively, sup-port the view that sncRNAs (5S, 5.8S, sn-, and snoRNAs) with near mature length are subject to undergo polyadenylation by Pap1p/Trf4/5p before their degradation by Rrp6/47p.

### Polyadenylated snRNAs are incorporated in the spliceosome and are functional

Since *rrp6*-Δ and *rrp47*-Δ strains accumulate a huge amount of polyadenylated forms of varieties of sncRNAs, we query if these polyadenylated RNAs in the *rrp6*-Δ strain are still functional. We argued that since polyadenylated snRNA species accumulate in the *rrp6*-Δ strain, these adenylated snRNAs might interfere with the splicing activity of the resulting spliceosomes. We first examined if the splicing of two intron-containing mRNAs, pre-*ACT1* and pre-*CYH2* mRNAs become impaired in an *rrp6*-Δ strain (**Fig. 7F**). As shown in **Fig. 7F**, the efficiency of the splicing of these pre-mRNAs in both *rrp6*-Δ and *rrp47*-Δ strains remained very similar at both 25°C and 37°C relative to that found in the WT strain (compare lane 2 with lane 1, and lane 5 with lane 4). Their splicing, in contrast, was significantly compromised in a conditional splicing-defective *prp2-1* strain [82] at the non-permissive temperature of 37°C thereby reinforcing the argument that loss of functional Rrp6p did not affect the splicing of these pre-RNAs. This observation suggests that perhaps the majority of the spliceosomes in the *rrp6*-Δ and *rrp47*-Δ strains is functional and support the splicing reaction *in vivo*, which prompted us to explore if adenylated snRNAs that accumulated in the *rrp6*-Δ and *rrp47*-Δ strains in large abundance are also incorporated into the functional spliceosomes. Consequently, we immunopurified spliceosome from the WT and *rrp6*-Δ strain using Prp4p as the bait followed by a careful examination of the purity of the isolated spliceosome (**Fig. 7G**). As shown in this figure, all the tested spliceosomal components, Prp8p, Prp80p and Spp381p were found to be present in equal abundance in the IP fraction from both the WT and *rrp6*-Δ strains, whereas, none of the non-spliceosomal proteins such as Nrd1p or Rrp6p could be detected in these fractions (**Fig. 7G**). This observation indicates the authenticity of our purification procedure and purity of the spliceosomal preparation. Next, we evaluated the abundance of the polyadenylated and non-polyadenylated U6 and U1 snRNAs in these spliceosomal preparations from both WT and *rrp6*-Δ strains as described in the Materials and Methods section (**Fig. 7H**). As shown in **Fig. 7H**, substantial amount of polyadenylated versions of both U6 and U1 snRNAs were detected in the immunopurified spliceosome preparations in a *rrp6*-Δ strain.

Interestingly, the ratio of the polyadenylated to non-polyadenylated U6 and U1 snRNAs estimated from the spliceosome preparations (0.07 and 10.5 for U6 in WT and *rrp6*-Δ strains and 0.089 and 5.4 for U1 snRNA in WT and *rrp6*-Δ strains) mimics their estimated ratio of polyadenylated to non-polyadenylated fractions in total RNA preparations (0.09 and 13.5 for U6 in WT and *rrp6*-Δ strains and 0.087 and 7.1 for U1 snRNA in WT and *rrp6*-Δ strains) in the respective strains (Fig. S9B). These findings therefore support the argument that the part of the polyadenylated U1 and U6 snRNAs that largely accumulate in the *rrp6*-Δ strain are functional and they assemble in the functional spliceosome particles.

## DISCUSSION

In this investigation, we unveiled a novel functional role of the major nuclear 3′→5′ exoribonuclease, Rrp6p, and its cofactor Rrp47p in the degradation of polyadenylated versions of several mature sncRNAs, including 5S, 5.8S rRNAs, all sn- and some select snoRNAs in the baker's yeast *S. cerevisiae*. Data from RT-qPCR and northern blot analysis revealed a significant accumulation of mature polyadenylated forms of all of these ncRNAs selectively in *rrp6*-Δ and *rrp47*-Δ strains. Notably, multiple studies have previously established that Rrp6p is involved in the processing/trimming reactions of several ncRNAs [14, 16, 24, 63–67] and these studies demonstrated a dramatic accumulation of the precursor forms of some of these sncRNAs. However, these earlier studies neither explored if Rrp6p targets/degrades their mature forms nor demonstrated it [23, 44, 67, 96–99] and all of them showed that only precursor forms of these RNAs accumulated in the *rrp6*-Δ strain. Moreover, none of the studies clearly addressed if Rrp6p alone is involved in this trimming/degradation activity or if it requires the involvement of any ancillary factor(s) such as core nuclear exosome for such decay. Finally, none of them investigated the profiles of polyadenylation of these sncRNAs in WT and *rrp6*-Δ yeast strains. In this work, we systematically tested all of these possibilities and collectively, our observations support the idea that the polyadenylated forms of mature sncRNAs undergo rapid degradation in an exosome-independent and Rrp6p-Rrp47p-dependent manner following their polyadenylation catalyzed either by Pap1p and/or by Pap1p and Trf4p.

Rrp6p is involved in the mature 3′-end formation of 5.8S rRNA and many snoRNAs by trimming the 3′-extended nucleotides of their precursors to their mature length [6, 16, 52]. Arguably, therefore, enhancement of steady-state levels of sncRNAs in the *rrp6*-Δ strain in our experiments may be interpreted as an event happening due to accumulation of 3′-extended unprocessed intermediates [23, 52, 67, 83–86]. Our data from northern blot analysis (**Figs. 1C**, S1D-E, and S2B-D), however, strongly suggested that besides the accumulation of 5.8S+30 precursor species, a substantial amount of mature 5.8S rRNA species was also accumulated in both *rrp6*-Δ and *rrp47*-Δ strains. It should be noted here that although the intensity of the 5.8S+30 precursor rRNA band was higher than that of the mature 5.8S rRNA species as has been reported in a previous study (for example, see Fig. 4A in Ref. [44]), this pattern is not always consistent. In fact, there were instances, where the intensities of mature 5.8S rRNA bands appeared much stronger than the precursor 5.8S+30 species. For instance, Fig. 6C of the same study [44] and Fig. 1C of the study by Allmang *et al*. (1999) [24] displayed clear instances where the mature 5.8S rRNA accumulated in much stronger abundance than its precursor 5.8S+30 species [24, 44]. Thus, the notion that only the precursor 5.8S rRNA species would preferentially accumulate in the *rrp6*-Δ strain is not general and universal, which is consistent with our northern data.

Analysis of polyadenylation sites of the 5S and 5.8S rRNAs (**Fig. 7**) demonstrated that in addition to precursor 5.8S+30 species, transcripts corresponding to the mature RNAs accumulate specifically in *rrp6*-Δ and *rrp47*-Δ strains, which have either small extensions or recessions of a few nucleotides (**Fig. 7A-B**) thereby bolstering the view that both the mature and precursor 5S and 5.8S rRNA species accumulate in the *rrp6*-Δ strain. In good agreement of this view, the increase in the steady-state levels of 5S and 5.8S rRNAs correlated well with their diminished decay rates in the *rrp6*-Δ strain (Figs. S4–S6). To bolster this notion, we carried out precise estimations of the abundance of mature ncRNAs and their precursors together using primer-sets corresponding to the CDS region (**Figs. 1A** and S2A, **Table 1** and **2**) and various precursor fragments using primer-sets spanning mature to 3′-extended regions of these ncRNAs (**Table 1** and **2**) in separate RT-qPCR assays. The RT-qPCR signals of their precursor species were subsequently subtracted from the combined signals from CDS region and precursor together to estimate the net accumulation of the mature species in the *rrp6*-Δ strain. These estimations independently revealed that the mature polyadenylated forms of all of these sncRNAs displayed accumulations from ~25 to ~120 fold on an average in the *rrp6*-Δ strain relative to the WT (see **Table 1** and **2**). Thus, analyses from RT-qPCR, northern blot and polyadenylation sites collectively led us to conclude that Rrp6p along with Rrp47p targets and degrades the polyadenylated version of these mature sncRNAs in the core exosome-independent manner besides trimming their 3′-extended precursors.

In a good agreement with RT-qPCR data, analyses of the polyadenylation profiles of these sncRNAs employing LM-PAT analyses suggest that the abundance of their adenylated forms are significantly higher in *rrp6*-Δ and *rrp47*-Δ strains relative to the WT (**Fig. 7A-D**). Interestingly, the polyadenylated 5S and 5.8S rRNA species with adenylation sites at sites -3, -2 and +11 in 5S and at sites +1 and +24/25/28 in 5.8S with respect to their mature ends were detected in both WT and *rrp6*-Δ strains indicating that the polyadenylation events indeed happen in both of them. However, their abundance in the WT strain is extremely low because of their rapid degradation by Rrp6p/47p. Therefore, it is likely that polyadenylated 5S and 5.8S rRNAs may represent the products of incomplete/aberrant processing events.

Estimation of the length of the poly(A) tail of the accumulated polyadenylated LM-PAT products in *rrp6*-Δ and *rrp47*-Δ strain suggests that the average length of their polyadenylated tails are approximately twelve to 87 residues long. This data were further confirmed by the sequence analyses of the multiple random LM-PAT clones of 5S and 5.8S rRNAs (**Table 5**, Supplementary Table S8). The observed length of their polyadenylated tail (much longer than four to six adenylate residues - a signature of Trf4p/Trf5p polyadenylation) is consistent with the involvement of Pap1p in their polyadenylation, which is further supported by the finding of absence of any LM-PAT products of these ncRNAs in the *pap1-1* strain at the non-permissive temperature of 37°C (see next). Notably, two known poly(A) polymerases, Trf4p and Pap1p, were demonstrated to be essential in polyadenylating the sncRNAs [14, 28, 65, 87]. It should be mentioned here that their fundamental feature of catalysis, however, is quite different. Pap1p is principally responsible for adding long poly(A) tails to the nuclear pre-mRNA precursors in a processive manner, and the speed of the reaction is breakneck and efficient. In contrast, the rate of catalysis by Trf4p is very slow and inefficient, and it adds only a few residues of adenine in a distributive manner [14]. Although a few previous studies demonstrated that non-canonical poly(A) polymerase Trf4/5p plays a major role in the polyadenylation of tRNA, 5S rRNA, many sn- and snoRNAs [14, 28, 65, 66] the combined involvement of both the major and non-canonical poly(A) polymerase, Pap1p and Trf4p was also documented in the polyadenylation of snoRNAs [16]. Our data, which is consistent with the latter finding, support the idea that Pap1p plays a major role in the polyadenylation of the sncRNAs. In agreement with this view, the involvement of Pap1p in the polyadenylation of these sncRNAs predicts that the average length of these sncRNAs' tails would be longer than four to six adenylate residues. This speculation is supported by the length of poly(A) tracts (approximately ten to 19 adenylate residues present in 5S, U1 and snR10, and twelve to87 adenylate residues in 5.8S rRNAs) in the *rrp6*-Δ strain as determined from our LM-PAT analysis followed by sequencing these LM-PAT products (**Fig. 7**, **Table 5** and S8). Consistent with this view, these polyadenylated RNAs were also found to be (i) efficiently primed by oligo-dT_12-18_ primer for cDNA synthesis, and (ii) in extremely low abundance in a *pap1-1 rrp6*-Δ double mutant strain at 37°C compared to the *rrp6*-Δ single mutant (**Fig. 6A-D**). Although the sequence of their participation in polyadenylating these RNAs is not verified experimentally in our study, it is reasonable to suggest that Trf4p participates at the initial stage to form a short oligo tail of four to six nucleotides, which is used as a primer for the subsequent polyadenylation by Pap1p. A similar sequence of participation of Trf4p and Pap1p was also demonstrated in the polyadenylation event during the synthesis/maturation of snoRNAs [16].

Accumulation of a huge burst of polyadenylated rRNAs, sn- and snoRNAs in the *rrp6*-Δ strain let us explore if these polyadenylated RNAs are incorporated into spliceosomes by probing the ratio of polyadenylated vs. non-polyadenylated U6 and U1 snRNAs in spliceosome and total cellular RNA pool from WT and *rrp6*-Δ strains. This analysis revealed that (i) indeed the polyadenylated U6 and U1 snRNAs are assembled in the spliceosomes and (ii) the ratio of polyadenylated to non-polyadenylated fractions of the spliceosome-assembled snRNAs mimics their corresponding ratio found in the total RNA pool (**Fig. 7G-H**, supplementary Fig. S11D). These findings indicate that apparently no filtering mechanism exists that prevents these polyadenylated snRNAs from incorporating into the spliceosomes in the *rrp6*-Δ strain. More interestingly, incorporation of these adenylated snRNAs did not render the spliceosomes non-functional since a comparison of splicing efficiencies of two intron-containing pre-mRNAs, *ACT1* and *CYH2* in WT, *rrp6*-Δ and *rrp47*-Δ strains revealed no significant impairment of their splicing in the latter two strains (**Fig. 7F**). Notably, a previous observation suggests that the precursor 5.8S+30 transcripts which are polyadenylated in a strain that carries a defective allele of *RRP6* (*rrp6-1*) was also incorporated into polysomes, however, the efficiency of the incorporation of these 5.8S+30 precursor is not as efficient as that of the mature 5.8S [52]. Collectively, therefore, these findings are supportive of the idea that polyadenylated versions 5.8S rRNAs, U6 and U1 snRNAs incorporate into the ribosomal and spliceosomal particles respectively and these altered ribonucleoprotein complexes are apparently functional. Further research is required to have an insight into their incorporation mechanism and to resolve if any exclusion mechanism exists to keep these polyadenylated RNAs out form being assembled.

A previous investigation demonstrated that polyadenylated transcripts accumulate in a distinct site in the nucleolus called the poly(A) domain, which is different from the sites of many of these ncRNAs' maturation. Formation of the poly(A) domain also requires Rrp6p, Pap1p, Trf4p, and components of the nuclear exosome, Rrp41p, and the TRAMP component Mtr4p [64]. Notably, our data argued against the contribution of any core exosomal component (except Rrp6p) and the TRAMP component (except Trf4p) in the accumulation of polyadenylated transcripts. Although the poly(A) domain was found to be enriched with polyadenylated and 3′-extended precursor form of U14 snoRNA and possibly their mature form, this study neither did evaluate if any other kinds of RNAs are present in such poly(A) domain, nor did it address if the loss of Rrp47p leads to the poly(A) domain formation [64]. It will be interesting to examine if the polyadenylated ncRNAs that accumulate in *rrp6*-Δ/*rrp47*-Δ strains in our strain background indeed localize in a distinct foci in the nucleus. Nevertheless, it remains to determine the exact functional relationship between the poly(A) domain [64] and the accumulation of polyadenylated forms of mature-length sncRNAs in the nucleus of *rrp6*-Δ *rrp47*-Δ strains.

Reanalysis of previous genome-wide RNA-sequencing datasets [73–75] and the resulting differential expression profiles of the snoRNAs in WT, *rrp4-G226D*, *dis3-1,* and *rrp6*-Δ strains revealed that many snoRNAs displayed a significant up-regulation in the *rrp6*-Δ strain (**Fig. 2A-C**). In contrast, only a handful of these snoRNAs exhibited a very modest upregulation in *rrp4-G226D* and *dis3-1* strains (**Fig. 2A-E**). Despite the considerable accumulation of sequence reads in their 3′-extended regions in the *rrp6*-Δ strain, a comparison of the relative abundance of these reads in the CDS and 3′-extended regions indicates that the abundance of the reads in the mature sequences are significantly higher relative to 3′-extended reads (**Fig. 2F**) suggesting a net accumulation of their mature forms. Collective data thus support the conclusion that the mature/near mature forms of these snoRNAs accumulate in the *rrp6*-Δ strain.

**Figure 8.**
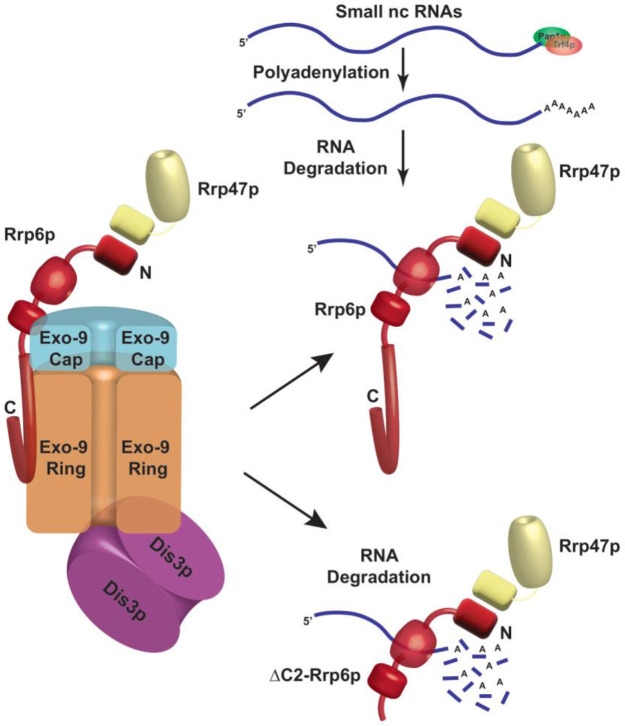
FIGURE 8: Cartoon showing a proposed model of the Rrp6p and Rrp47p-dependent degradation of polyadenylated mature/processed forms of sncRNAs in *S. cerevisiae*. Low molecular weight sncRNAs undergo polyadenylation by both the canonical and non-canonical poly(A) polymerases, Pap1p and Trf4p. This polyadenylated fraction of sncRNAs are then targeted by the Rrp6p-Rrp47p complex in the core-exosome independent manner. Notably, a C-terminally truncated version of Rrp6p that lacks the physical association with the core nuclear exosome is equally competent in targeting and degrading these sncRNAs.

Genetic evidence supporting the absolute requirements of only Rrp6p and Rrp47p for the nuclear degradation of the polyadenylated forms of these sncRNAs and dispensability of the core exosome, the TRAMP, and the CTEXT components for this function is correlative to the demonstration that Rrp6p/Rrp47p co-purify together as an exosome-independent separate protein complex. This observation was further bolstered by our finding that mutant Rrp6p-ΔC2 lacking its association with the core nuclear exosome, still supports the nuclear decay of these sncRNAs (**Fig. 4B-J**) and displays strong interactions with the polyadenylated forms of these RNAs (**Figs. 5C-H**, S7G). Furthermore, the association of the polyadenylated sncRNAs with the exosome-free Rrp6p/47p complex specifically established the notion that the exosome-independent free cellular Rrp6p/47p pool directs the degradation of the polyadenylated forms of sncRNAs (**Figs. 3** and **5**, Supplementary Fig. S7G). Collective evidence thus strengthens the view that the nuclear degradation of sncRNAs exemplifies another novel instance of an exosome-independent function of Rrp6p in *S. cerevisiae* (**Fig. 8**). However, it remains to evaluate if the Pap1p and Trf4p constitute a part of the same complex or exert their effects independently of this complex. Although the complete protein and RNA composition of the Rrp6-Rrp47p complex are yet to be revealed, it is reasonable to state that this complex may be responsible for carrying out the degradation of polyadenylated mature-length ncRNAs in the nucleus. Future work would throw more light on this unresolved issue.

Notably, accumulation of polyadenylated forms of small ncRNAs in the *rrp6*-Δ yeast strain is reminiscent of many previous findings [14, 16, 24, 63–67], where diverse species of non-coding RNAs such as polyadenylated initiator tRNA^met^, 5S rRNA and other ribosomal RNAs [28, 63, 65], snRNAs and snoRNAs [14, 16, 28, 66] were reported to accumulate in an *rrp6*-Δ strain. It should be noted here that our findings on the polyadenylated species accumulating in the *rrp6*-Δ strain presented in this investigation differ significantly from some of these earlier findings and some of our observations were not demonstrated in any of the previous studies. First, although polyadenylated 18S and 25S rRNAs were reported to accumulate in the *rrp6*-Δ mutant strain [28, 63], we did not observe any enhancement in the levels of either total or polyadenylated forms of these rRNAs. Second, we specifically demonstrated that a major contribution towards the total accumulation of the poly(A) RNA species in *rrp6*-Δ/*rrp47*-Δ strains is contributed by the accumulation of their mature forms in addition to the precursor species. Third, none of these studies demonstrated the involvement of Rrp47p. Fourth, a few studies inferred involvement of core exosome (Exo-10) in the degradation of these polyadenylated species primarily from the involvement of core nuclear exosome specific subunit Rrp6p [28, 66]. In contrast to these findings, we provide strong biochemical and genetic evidence that Rrp6 and Rrp47p perform the degradation of these polyadenylated RNAs independently of the core exosome (**Figs. 3-5**). Fifth, while many earlier reports noted that non-canonical poly(A) polymerases, Trf4/5 play the vital role in the polyadenylation of these ncRNAs [66], our data strongly argues for the involvement of the canonical poly(A) polymerase Pap1p in their polyadenylation process in agreement of a few earlier reports [16, 63]. Our findings therefore represent several novel aspects of the Rrp6p/47p dependent polyadenylation of the sncRNAs in baker's yeast that was never demonstrated in any of the earlier studies.

Although the physiological significance of this observation is not quite clear at this point, it is worth mentioning here that two conflicting views persist on the ultimate goal of the polyadenylation of the ncRNAs in eukaryotes. One general notion that emerged from many preceding studies supports the idea that polyadenylation of non-coding transcripts aims to target them for exosome dependent degradation and hence are considered crucial for their surveillance [14, 28, 65, 66]. Remarkably, an antagonistic view was also proposed, in which it was suggested that polyadenylation of some these ncRNAs such as snoRNAs is contributory and vital for their final maturation and 3′-end formation [16, 83, 88]. Notably, the evidence from our investigation suggests that polyadenylated U1 and U6 snRNAs are functional and are assembled into spliceosome both in the WT and *rrp6*-Δ strain (**Fig. 7F-H**), which is reminiscent of a previous study where polyadenylated 5.8S+30 precursor species in the *rrp6*-Δ strain was demonstrated to incorporate into the polyribosomes [52]. These observations do not support the general notion that polyadenylated ncRNAs are generally non-functional and is obligatorily targeted for degradation. In fact, the functionality associated with the polyadenylated 5.8S rRNA precursor as demonstrated previously [52] and that of polyadenylated U6/U1 snRNAs is indicative of a potential physiological role of this process. Furthermore, the Rrp6p-dependent decay of polyadenylated rRNA species may provide a major mechanism that regulates the speed and efficiency of ribosome biogenesis during the stress response. As ribosome biogenesis is an incredibly energy-requiring process, a cell would prefer to speed up the ribosome biogenesis process during a favorable environment and growth. During any stress, the biogenesis of ribosomes would likely be restricted and regulated. Although the exact molecular mechanism is not known, it might not be unreasonable to invoke that, during stress, perhaps this exosome-independent Rrp6p/47p dependent degradation of polyadenylated sncRNAs could serve as a possible mechanism to regulate and limit ribosome biosynthesis, which in turn could induce the proteolysis of the ribosomal proteins[89]. Recent demonstration of rRNA processing inhibition during Integrated Stress Response (ISR) in mammalian cells [90] supports this hypothesis. Future research addressing the potential functional and regulatory role of this process is therefore vital to resolve this issue.

## MATERIAL AND METHODS

### Nomenclature, yeast strains, media, yeast genetics, plasmids, and oligonucleotides

Standard genetic nomenclatures, growth media, procedures of yeast cultivation, genetic analysis, cloning, and basic molecular biology were used as described before[91]. Yeast strains deficient in the components of the nuclear exosome, the TRAMP, and the CTEXT complexes were constructed using the one-step gene replacement method. The yeast strain carrying an *rrp4-1* mutant allele was constructed by standard two-step replacement procedure [64]. Oligonucleotides were obtained commercially from Integrated DNA Technology (Coralville, IN, USA). Yeast strains, plasmids, and oligonucleotides used in this study are listed in Supplementary Tables S1 (yeast strains), S2 (plasmids), S3, S4, S5, and S6 (Oligonucleotides) in the Supplementary Data.

### Inactivation of *rrp4-1* strain Galactose induction of *GAL10::RRP41* and *GAL10::DIS3* gene

The yeast strain carrying an *rrp4-1* mutation was grown in YPD medium at 25°C until the OD_600_ of the cultures reached 0.6. Half of the culture was then continued to grow until the OD_600_ of the culture reached 0.9-1.0. The other half of the culture was subjected to a temperature shift to 37°C followed by the growth for two to eight additional hours unless stated otherwise. Both the culture from the permissive and restrictive growth were harvested and total RNA and cDNA samples were isolated for further downstream analysis as described below.

The native promoters of the *RRP41* and *DIS3* genes in the WT strain were replaced by p*GAL1/GAL10* promoter by homologous recombination and confirmed using genomic PCR to obtain exosome deficient strains *HIS3-GAL10:: protA-RRP41* and *HIS3-GAL10:: protA-DIS3*. These strains were grown in a non-inducible, non-repressible 2% raffinose sucrose medium (YPRS) until the OD_600_ of the culture reached 0.6. Galactose was then added to the cultures at 2% final concentration, followed by the growth of the cultures until the OD_600_ of the cultures reached 0.9-1.0 unless stated otherwise in some experiments. Glucose was then added to the cultures followed by growth for an additional 2-24 hours followed by harvesting of the cells. Total RNA samples subsequently were isolated from these cells for further downstream analysis as described below. For some additional sets of experiments, *GAL10::RRP41* and *GAL10::DIS3* strains were grown in the presence of non-inducible raffinose/sucrose medium until the OD_600_ of the cultures reached 0.9-1.0 followed by addition of 2% glucose and continued to grow for various times up to 24 hours. Total RNA samples were then isolated and processed for further downstream analysis.

### Construction of the yeast strain carrying an Rrp6-ΔC2 allele by Reverse PCR and ligation

Genomic copy of the full-length *RRP6* gene (2.2 kb) along with 1.0 kb upstream and 1.0 kb downstream regions was amplified with high fidelity PCR enzyme mix (Fermentas Inc.) followed by cloning of the PCR product in pJET1.2/blunt vector (Fermentas Inc.). This insert containing the *RRP6*-FL (Full-length *RRP6*) allele was subcloned in yeast vector pRS315, which was referred to as pRRP6-FL. Plasmid Rrp6p-ΔC2 (deletes amino acids 523–733) was constructed from the *RRP6*-FL plasmid using a Reverse PCR-based technology with primers oBD677 and oBD678, followed by blunt-end ligation of the 7 kb PCR product using T4 DNA ligase (Fermentas Inc). The plasmids pRRP6-FL and pRrp6p-ΔC2 were then transformed into the *rrp6::URA3* yeast strain (yBD 129) to generate the strain harboring the C-terminal truncated version of Rrp6p (yBD 527).

### RNA analyses and determination of steady-state of levels of various RNAs

Isolation of the total RNA sample was carried out as described previously [34]. Briefly, desired yeast strains were grown in YPD liquid medium followed by harvesting of the cells when the culture reaches log phase (OD_600_ = 0.8 to 1.0). The cell pellet was resuspended, lysed, and extracted in presence of an acid phenol-chloroform–IAA (25:24:1, pH-4.5) mixture in presence of glass beads. The suspension was then centrifuged and the aqueous phase was recovered and subjected to extraction with acid phenol-chloroform-IAA three more times. The RNA was finally recovered by precipitating the aqueous phase from the last extraction step with RNAase-free absolute ethanol followed by centrifugation of the suspension, washing the pellet with 75% ethanol, drying the pellet, and resuspension in RNase-free dH_2_O. The total RNA samples were estimated spectrophotometrically.

For cDNA preparation, 2 µg of total RNA samples were treated with RNase-free DNase I (Fermentas Inc.) at 37°C for 30 minutes and then incubated with 10 mM EDTA at 65°C for 10 minutes. This was followed by cDNA synthesis by reverse transcription of 1 µg of purified RNA by MMLV reverse transcriptase (BioBharati) for 50 minutes at 42°C using either random hexamers or oligo-dT_(12-18)_ primers. The cDNA samples were quantified using Qubit^®^ ds DNA-HS Assay Kit (Life Technologies, USA) following the recommendation of the manufacturer.

### Real-Time RT-qPCR

2 ng of quantified cDNA prepared from three to five biological replicates were used to quantify the levels of specific RNAs such as rRNAs, snRNAs, and snoRNAs by real-time qPCR assays using target-specific primers for mature and precursor regions with standard SYBR Green Technology using Power SYBR® Green PCR Master Mix (Applied Biosystems). The qPCR assays were run in triplicate and conducted using an Applied Biosystems StepOne^TM^ real-time PCR system to determine the absolute quantification of individual target mRNAs. For each target, ΔΔ^-Ct^ method was used, in which *SCR1* RNA (in the case of Random Primer) and *ACT1* mRNA (in the case of oligo dT primer) were used as the internal control, and the abundance was determined from fluorescence by the StepOne^TM^ software version 2.2.2 (Applied Biosystems).

### Northern Blot Analysis

Northern blot analysis was carried out by following the procedure described by Briggs *et al*. [52]. Briefly, 20-40 μg of total RNA sample was mixed with formaldehyde loading dye, heated at 65°C for 15 minutes, and then quickly transferred to ice and incubated for one minute. The denatured RNA samples were then separated on a 15% polyacrylamide-urea gel at a constant voltage of 150 V in 0.5X TBE buffer for 2-3 hours. Separated RNA was then transferred to a positively charged nylon membrane in by electro-transfer in 0.5X TBE buffer at 15 V overnight. The membrane was washed with 2X SSC buffer, cross-linked under 260 nm UV for 2 minutes, washed with sterile water, and air-dried. The membrane was then subjected to pre-hybridization in Roche DIG-Easy Hyb Hybridization buffer (REF: 11603558001) for 1 hour at 37°C in a hybridization oven with gentle shaking, followed by hybridization with 2 ng/ml of respective DIG-labeled oligonucleotide probes at 5′-end (Sequences of the respective probes are given in supplementary Table S5) for overnight at 37°C. Post hybridization, the membranes were primarily washed twice with buffer containing 2X SSC and 0.1% SDS at 27°C for 5 minutes each and then washed twice with buffer containing 0.1X SSC and 0.1% SDS at 27°C for 15 minutes each. After washing the membrane with the Roche DIG wash-buffer, the membranes were prepared for detection using Roche DIG nucleic acid detection kit (REF 11175041910) as recommended by the manufacturer. Subsequently, the membrane was left for blocking with 1X blocking solution at 27°C for 30 minutes and then incubated with Roche Anti-DIG antibody in 1:5000 dilutions in 1X blocking buffer for 1 hour at 4°C. Next, the membranes were washed twice with 1X Roche Washing buffer for 15 minutes at 27°C and incubated in the Roche 1X detection buffer for 5 minutes at the same temperature. Finally, BCIP/NBT solution was added to the detection buffer for color development on the membranes in dark for 30 minutes at 27°C. The reaction was stopped with sterile distilled water. The relevant bands were quantified by Image J (NIH, USA) software following standard method.

### Analysis of stability of specific ncRNAs using Transcription Shut-off experiment:

The stabilities of the various RNAs were determined by the inhibition of transcription with transcription inhibitors BMH-21 (for RNA Pol I Transcripts: 5.8S rRNA and ITS1), 1, 10-phenanthroline (for RNA-Pol II transcripts: mRNAs; snRNAs; and snoRNAs, SNR10 and SNR13), and ML-60218 (for RNA Pol III Transcripts: 5S rRNA and U6 snRNA) at 30°C as described before [32]. Briefly, specific yeast strains were grown at 30°C till the cultures attained mid-logarithmic phase (OD_600_ = 0.6 to 0.8), when any one of the transcription inhibitors BMH-21; 1, 10-phenanthroline; or ML-60218 were added to the growing culture at 25 µM, 100 µg/ml, and 16 µM final concentrations respectively. 10 ml aliquots of cultures were withdrawn at various time intervals after transcription shut-off followed by harvesting of the cells and snap-freezing the pellets in dry ice. Total RNA was then isolated as described above and the ncRNA levels were quantified by RT-qPCR assay from random-primed cDNA, and the signals associated with the specific ncRNA were normalized against *SCR1* signals. Specific mRNAs' decay rates and half-lives were estimated with the regression analysis program (Graphpad Prism version 7.04) using a single exponential decay formula (assuming mRNA decay follows first-order kinetics); y = 100e^−bx^ was used.

### Analysis of the polyadenylation sites and poly(A) tail length by Ligation Mediated Poly(A) Tail Assay (LM-PAT)

LM-PAT assay was carried out as described previously [81]. Briefly, 10 µg total RNA samples from WT, *rrp6*-Δ, and *rrp47*-Δ were incubated with 20 ng Oligo dT_12-18_ at 65°C for 10 minutes. To this mixture, 4 µl 5X RT buffer, 1 µl 10 mM dNTP, 1 µl 10 mM ATP, 2 µl 0.1 M DTT, 1 µl RNase Inhibitor (Invitrogen), and 1 µl T_4_ DNA ligase (Invitrogen) were added and incubated at 42°C for 30 minutes and further incubated at 12°C. Next, 1 µl of 200 ng/µl Oligo dT anchor primer (OBD 392: 5‘GCGAGCTCCGCGGCCGCGTTTTTTTTTTTT-3’) was added to the mixture and incubated at 12°C for 2 hours and stored at 42°C for 3 minutes. Finally, 2 µl of 10 U/ml MMLV reverse transcriptase (Invitrogen) was added and the mixture was incubated at 42°C for 1.5 hours followed by the inactivation of the enzyme at 85°C for 10 minutes. The cDNA prepared was then subjected to PCR amplification to amplify either the mature sequence of 5S, 5.8S, U1, and snR10 RNAs or their polyadenylated versions using sense primer specific to their 5′-termini and anchor-specific anti-sense primer (**Fig. 7A**). The sequences of the primers used to amplify LM-Products of various RNAs are presented in supplementary Table S6. For amplification of 5S mature sequence, primer-sets oBD343-856 and poly(A) 5S RNA, primer-sets oBD343-392 were used. For amplification of the 5.8S mature sequence, primer-sets oBD188-189 and poly(A) 5.8S RNA, primer-sets oBD188-392 were used. For amplification of the U1 RNA mature sequence, primer-sets oBD857-858 and poly(A) U1 snRNA, primer-sets oBD857-392 were used. For amplification of the snR10 mature sequence, primer-sets oBD859-860 and poly(A) snR10 RNA, primer-sets oBD859-392 were used. The LM-PAT PCR products of 5S and 5.8S rRNAs were subsequently cloned and each clone was subjected to sequencing. The sequence data thus obtained were mapped to genomic sequence and compared.

### Protein analyses by western blot

Specific yeast strain expressing either a native or epitope-tagged protein was grown overnight at 30°C in YPD broth. Cells were harvested by centrifugation at 4000g for 7 min followed by snap freezing of the cell pellets in liquid nitrogen and storage at –70°C. Frozen pellets were thawed on ice and resuspended in 1 ml of Buffer A (50 mM Tris–HCl, pH 7.5, 150 mM NaCl, 5 mM EDTA, 1 mM DTT, 1 mM PMSF) supplemented with Protease Inhibitor Cocktail (Abcam ab201111, UK) and the cells were lysed by vortexing 10–15 times with glass beads followed by clarification of the particulate fraction by centrifugation. Supernatants were collected by centrifugation at 16,400g for 20 min and saved as the total soluble protein fraction for further analysis. Protein concentration was determined by the Bradford reagent assay kit (Bio-Rad Inc., Valencia, CA, USA). For Western Analysis, 60-80 µg of total protein was used, resolved in 10% SDS polyacrylamide gel. The separated proteins were transferred to PVDF membrane at 50-100 mA O/N for immunoblotting. Blots were blocked with 5% skimmed milk in Tris-buffered saline with 0.1% Tween 20 (TBST: 10 mM Tris, 150 mM NaCl, 0.1% Tween 20) and incubated with primary antibodies for specific proteins for 1 hour at 27°C diluted in 5% BSA (specifications of antibodies are presented in Supplementary Table S7). Blots were washed in 1X TBST and incubated in HRP-conjugated secondary anti-rabbit or anti-mouse antibody, each diluted at 1:3000 in wash buffer for one hour at room temperature. Immuno-reactive bands were developed and detected by chemiluminescence (ECL imager kit, Abcam), and the images were captured either by X-ray film or myECL Chemidoc Imager (Thermo Scientific, USA).

### Co-Immunoprecipitation

Cells were grown in 50 ml YPD until OD_600_ of the cultures reached 2.7 to 3.0. The cell lysate was prepared in 1 ml of Buffer A (50 mM Tris–HCl pH 7.5, 150 mM NaCl, 5 mM EDTA, 1 mM DTT, 1 mM PMSF). Approximately, 50 µl bed volume of Protein A^PLUS^ Agarose Beads (BioBharati Life Sciences Pvt. Ltd., India) per reaction were equilibrated twice with ten volumes of Buffer A. he beads for each washing were resuspended in Buffer A and kept on a rotator wheel for 5 minutes at 4°C. The beads were centrifuged at 1700g for 3 minutes to remove residual Buffer A. The extract was then pre-cleared by adding the equilibrated beads and incubation on a rotator wheel at 4°C for 30 minutes. The extract was centrifuged at 1700g for 5 minutes at 4°C to remove the beads. To the pre-cleared protein extract, 2.5 µg of anti-TAP Ab (Thermo Fisher Scientific, CAB1001)/anti-Rrp6 Ab (a gift from Prof. Scott Butler, University of Rochester, USA)/anti-myc Ab (a gift from Dr. Suvendra Nath Bhattacharyya, CSIR-Indian Institute of Chemical Biology) was added and incubated for 4 hours on a rotator wheel at 4°C. To this extract, 100 µl (bed volume) of pre-equilibrated Protein A^PLUS^ Agarose Beads was added and incubated at 4°C on the rotator wheel overnight. The beads are washed thrice with ten volumes of Buffer A by rotating on the rotator wheel at 4°C for 10 minutes each. Finally, elution was performed by boiling the beads in 40 µl of SDS loading dye for 5 minutes. Samples were subsequently analyzed by SDS-PAGE gel electrophoresis followed by Western blot analysis.

### Analysis of binding profile of RNA by RNA Immunoprecipitation (RIP)

WT, *rrp6*-Δ and *rrp6*-ΔC2 strains were grown to the mid-logarithmic phase (OD_600_ = 0.6 to 0.8). Before the lysis of the cells intact yeast cells were cross-linked by UV-irradiation in a petri dish on ice. Cells were washed once with Buffer A (50 mM Tris-HCl pH 7.4, 140 mM NaCl, 1.8 mM MgCl_2_, 0.1% NP-40) and then resuspended in Buffer B (Buffer A supplemented with 0.5 mM DTT, 40 U/ml RNase Inhibitor, 1 mM PMSF, 0.2 mg/mL heparin, protease inhibitor) and lysed by vortexing using glass beads. Lysates were cleared by centrifugation for 10 min at 8,000 rpm/4°C. The lysate was quantified and pre-cleared for 30 minutes at 4°C. Protein A sepharose beads (Santa Cruz Biotechnology, TX, USA) were coated overnight at 4°C using Anti-Rrp6p antibody in Buffer B. 1 mg pre-cleared cell lysate was incubated with antibody-coated beads for 4 hours at 4°C on a rotator. Beads were washed twice in Buffer B for 5 min/4°C/rotator each, and once in Buffer C (50 mM Tris pH-7.4, 140 mM NaCl, 1.8 mM MgCl_2_, 0.01% NP-40, 10% glycerol, 1 mM PMSF, 0.5 mM DTT, 40 U/ml RNase Inhibitor, protease inhibitor) for 5 min/4°C/rotator each. After washing, beads were resuspended in 200 µl Buffer C supplemented with 1% final SDS and heated at 70°C for 45 min with constant mixing to de-crosslink and release antibody-bound protein-RNA complexes from the beads. Finally, RNA was isolated from the eluates by phenol-chloroform-Isoamyl alcohol (25:24:1, pH 4.5) extraction and cDNA was prepared using 1^st^ strand cDNA synthesis kit (Takara Bio Inc., Shiga, Japan) using oligo-dT_12-18_ primer. The immunoprecipitated RNAs (output) were quantitated by real-time PCR using primers specific to each mature and precursor RNA and normalized to a half dilution of the input sample. Amplifications were done in triplicate for each sample, and averages and standard deviations were calculated based on three independent experiments.

### Immunopurification of spliceosome

WT and *rrp6*-Δ yeast strains expressing genomically tagged Hexa-HA-Prp4p were grown overnight at 30°C in YPD liquid broth followed by three times UV irradiation for 10 minutes each to induce RNA-protein crosslink. Total cellular protein extract was then prepared as described above in ‘Protein Analysis by Western blot’ section. 1 mg of isolated protein was then incubated with anti-HA antibody (targeted against spliceosomal Hexa-HA-Prp4) (dilution: 1:1000) at 4°C in a rotating wheel for 4 hours, while the rest of the protein sample was saved as input. 100 µl of activated protein A-agarose beads was then added to the protein-antibody complex and incubated overnight at 4°C in a rotating wheel. Beads were subsequently pulled down by centrifugation at 1700g for 5 minutes. One fraction of the bead was boiled with SDS gel loading dye and was used for western blotting to check bait Prp4p-HA and the other bound spliceosomal proteins. The other fraction was subjected to isolation and quantification of bound non-polyadenylated and polyadenylated snRNA fractions following the procedure described above in ‘Analysis of Binding Profile of RNA by RNA Immunoprecipitation’ using either random or oligo-dT primers respectively by real-time PCR analysis.

### RNA-Protein crosslinking followed by protein purification using gel filtration column chromatography

Yeast strains were grown overnight at 30°C till the mid-logarithmic phase (OD_600_ = 3.0) followed by UV-irradiation to induce RNA-protein cross-link, cell harvesting, and total protein extraction using the standard protocol mentioned above. The protein extract was centrifuged twice at 40,000g for 10 minutes. The final supernatant was collected and stored at -80°C degrees ultra-freezer for future use. The gel filtration column (P-200 beads from BioRad) was soaked in water overnight at 27°C. The beads were then packed into a column (Bed Volume 60 ml) to form a packed bed followed by equilibration of the packed bed with Tris-HCl-NaCl buffer (0.2M Tris-HCl pH-8.0, 0.2M NaCl). Notably, before, the fractionation of the whole cellular extracts, the column was equilibrated with proteins of known MW markers. The protein extract was then loaded on to the column (60 ml bed volume) followed by the collection of sixty fractions (Fraction size: 500 µl). Each of these fractions was subsequently analyzed by SDS-PAGE and Western Blotting analysis. Further, fraction numbers 5-9 and 13-18 were subsequently pooled and subjected to RNA isolation and cDNA synthesis using oligo-dT_30_ anchor primer followed by RT-qPCR assay with 2 ng of cDNA using amplicons corresponding to CDS and 3′-extended precursor regions of various sncRNAs.

### Genomic data mining and analysis (RNA Sequencing)

Re-analysis of the previous dataset [73] available from Gene Expression Omnibus (GEO) under the accession number GSE135056 was done using the public server (usegalaxy.org) of The Galaxy Project [92]. The FASTQ files for the WT and *rrp6*-Δ RNA-Seq data were imported into the galaxy server using the SRA import tool Faster Download and Extract Reads in FASTQ format from NCBI SRA [93]. Quality checking and trimming as required were done with FastQC, Cutadapt [94], and MultiQC [95]. The processed reads were then aligned to the R64-1-1 (GCA_000146045.2) reference genome from ensemble.org with RNA STAR [96]. The annotation file used as a gene model was R64-1-1.101.gtf, also downloaded from ensemble.org. To confirm that most of the reads align to exons rather than intergenic regions (due to DNA contamination), Read Distribution from the RSeQC [97] tool suite was used to check the percentage of reads mapping to known genomic features. feature Counts [98] was then used to count the number of reads per annotated gene. To predict the strandedness of the library, Infer Experiment from the RSeQC [97] tool suite was used following the instructions from the galaxy tutorial, Reference-based RNA-Seq data analysis [99, 100] to determine how the sequencing library was configured. The information thus obtained from Infer Experiment [97] was used in feature Counts [98] with the option “Count fragments instead of reads” enabled to ultimately obtain the counts files for all the samples. The counts files for the technical replicates were merged by summing up the counts for each feature before feeding them into edgeR [76–78] so that the statistical analysis is performed on the biological replicates only thereby obtaining a better picture of the variance among the biological replicates. Since the number of replicates per condition was less than 12, the tools of choice [101] were either edgeR [76–78] or DESeq2 [102]. Coverage Plots were created using Integrative Genomics Viewer [103]. Volcano Plot and heatmap2 tools were used to create the volcano plots and heatmaps.

### Statistical analyses

The quantitative experiments reported in this paper (RNA steady-state levels and transcription shut-off Decay experiments) were performed using at least three independent sample sizes (biological replicate, *N* = 3 to 5). A given yeast strain was grown and treated under the same experimental conditions independently before a given experiment was conducted for each biological replicate. All the statistical parameters such as mean, standard deviations (SD), Standard Error of Mean (SEM), median, and P values were calculated using OriginPro8 and GraphPad Prism version 8.4 (GraphPad Software Inc., San Diego, CA, USA). P-values were calculated using Student's two-tailed t-test (unpaired) using the same program. The values of different statistical parameters (Biological Replicate, N, mean, median, SD, SEM, and P-value) corresponding to the individual experiments are given in a separate MS Excel File.

### Data availability statement

All the raw data leading to establishing the final results presented here are available in the supplementary material as a separate excel file ‘ChaudhuriA23 MCELL Res Art Raw Data File and Statistical Parameters’

## AUTHOR CONTRIBUTION

AC and SP performed experiments, MB accessed and analyzed the RNA-Seq data, AC and BD designed the experiments and analyzed the data, and AC and BD drafted and wrote the manuscript.

## SUPPLEMENTAL MATERIAL

Click here for supplemental data file.

All supplemental data for this article are available online at www.microbialcell.com/researcharticles/2024a-chaudhuri-microbial-cell/.

## Abbreviations:

CTEXT: – Cbc1p-Tif4631p-dependent EXosomal Targeting,
LM-PAT: – Ligation-Mediated PolyA Tail,
NNS complex: – Nrd1p-Nab3p-Sen1p complex,
rRNA: – ribosomal RNA,
sncRNA: – small non-coding RNA,
snRNA: – small nuclear RNA,
snoRNA: – small nucleolar RNA,
TRAMP: – TRf4p/5p-Air1p/2p-Mtr4p-Polyadenylation,
WT: – wild type
