# Supplementary material for: Polyadenylated versions of small non-coding RNAs in Saccharomyces cerevisiae are degraded by Rrp6p/Rrp47p independent of the core nuclear exosome
Source: Microb Cell. 2024 May 22;11:155–86. doi: 10.15698/mic2024.05.823 (PMC11115967; doi:10.15698/mic2024.05.823)
Supplement: Supplementary file 1 [file mic-11-155-s01.pdf]

Polyadenylated versions of small non-coding RNAs in *Saccharomyces cerevisiae* are degraded by Rrp6p/Rrp47p independent of the core nuclear exosome

Anusha Chaudhuri[2*¥], Soumita Paul[1¥], Mayukh Banerjea[1], & Biswadip Das[1*]

Department of Life Science and Biotechnology,
Jadavpur University, Kolkata -700032, India.

[*]**Correspondence**: Dr. Biswadip Das

Department of Life Science and Biotechnology, Jadavpur University, 188 Raja S.C. Mullick Road Kolkata – 700 032, West Bengal, India, Phone: 91-9748908607 (Cell),
E. mail: biswadipdas22@gmail.com, biswadip.das@jadavpuruniversity.in
Department of Life Science and Biotechnology, Jadavpur University, 188 Raja S.C. Mullick Road Kolkata – 700 032, West Bengal, India

[¥]These authors contributed equally to this work

[1]Department of Life Science and Biotechnology, Jadavpur University, 188 Raja S.C. Mullick Road Kolkata – 700 032, West Bengal, India

[2]Present Position: Team "DNA Recombination, Replication, and Genome Stability", UMR3348, Genome Integrity, RNA & Cancer 'GIRC' Unit, Institut Curie, Orsay 91405, France.

Submitted July 06, 2023

SUPPLEMENTARY DATA

Table S1: List and Genotypes of Yeast Strains used in this study

Strain No.	Complete genotype	Abbreviated Genotype	Reference
yBD5	*MAT*a *cyc1-512 ura3-52 trp2-1*	WT	(1)
yBD12	*MAT*a *cyc1-512 rrp6::URA3 ura3-52 trp2-1*	*rrp6*-Δ	(1)
yBD263	*MAT*a *cyc1-512 ura3-52 trp2-1 his3::hisG*	WT	*This Work
yBD264	*MAT*a *cyc1-512 ura3-52 trp2-1 his3::hisG cbc1::URA3*	*cbc1*-Δ	*This Work
yBD265	*MAT*a *cyc1-512 ura3-52 trp2-1 his3::hisG rrp6::URA3*	*rrp6*-Δ	*This Work
yBD266	*MAT*a *cyc1-512 ura3-52 trp2-1 HIS3::hisG tif4631::URA3*	*tif4631*-Δ	*This Work
yBD285	*MAT*a *cyc1-512 rrp4-1 ura3-52 trp2-1 his3::hisG*	*rrp4-1*	(2)
yBD298	*MAT*a *cyc1-512 ura3-52 trp2-1 his3::hisG HIS3-GAL::protA-RRP41*	*HIS3-GAL10:: protA-RRP41*	*This Work
yBD300	*MAT*a *cyc1-512 ura3-52 trp2-1 his3::hisG upf1::URA3*	*upf1*-Δ	*This Work
yBD306	*MAT*a *cyc1-512 ura3-52 trp2-1 his3::hisGtrf4::URA3*	*trf4*-Δ	*This Work
yBD315	*Mat a ade2-1 can1-100 his3-11,15 leu2-3,112 trp1-1 ura3-1 pep4::HIS3 RRP46-TAP:: kl TRP*	*TAP-RRP46::KlTRP1*	This work
YBD334	*MAT*a *cyc1-512 ura3-52 trp2-1 his3::hisG trf5::URA3*	*trf5*-Δ	*This Work
yBD454	*MAT*a *cyc1-512 ura3-52 trp2-1 his3::hisG YHR081w::kanMX4 (rrp47 Δ)*	*rrp47*-Δ	*This Work
yBD455	*MAT*a *cyc1-512 ura3-52 trp2-1 his3::hisG YNR024w::kanMX4 (mpp6 Δ)*	*mpp6*-Δ	*This Work
yBD458	*MAT*a *cyc1-512 ura3-52 trp2-1 his3::hisG air1::URA3*	*air1*-Δ	*This Work
yBD161	*MATa ade1 ade2 lys2 gal1 ura3-52*	WT	(3)
yBD162	*MAT*a *ade1 ade2 lys2 gal1 ura3-52 rrp6:: URA3*	*rrp6*-Δ	(3)
yBD163	*MATa ade1 ade2 lys2 gal1 ura3-52 pap1-1*	*pap1-1*	(4)
yBD179	*MATa ade1 ade2 lys2 gal1 ura3-52 pap1-1 rrp6::URA3*	*pap1-1 rrp6*-Δ	This work
yBD117	*Mat a leu2-3,-112 ura3-52 his3-Δ 200 trp1-Δ901 lys2-801*	WT	(5)
yBD129	*Mat* a *leu2-3,-112 ura3-52 his3-Δ 200 trp1-Δ901 lys2-801 rrp6::URA3*	*rrp6*-Δ	(5)
yBD413	*MAT*a *cyc1-512 ura3-52 trp2-1 his3::hisG rrp6::URA3 prrp6-3*	*rrp6-3*	(5)
yBD65	*MATα ura3-1 ade2-1 his3-11,15 leu2-3,112 trp1-1*	WT	B-15142
yBD66	*MATα ura3-1 ade2-1 his3-11,15 leu2-3,112 trp1-1 dob1-1*	*dob1-1*	B-15143
yBD421	*MATα ura3-1 ade2-1 his3-11,15 leu2-3,112 trp1-1 rrp6::URA3*	*rrp6*-Δ	*This work
yBD507	*MATa ura3Δ0; leu2Δ0; his3Δ1; met15Δ0 RRP47 TAP:: KANMX4*	*RRP47-TAP*	(6)
yBD508	*MATa; ura3Δ0; leu2Δ0; his3Δ1; met15Δ0TAP-RRP41-KAN*	*RRP41-TAP::KAN*	This work
yBD510	*MAT*a *cyc1-512 ura3-52 trp2- 1 his3::hisG YHR081w::kanMX4 (rrp47Δ): rrp6Δ::URA3 (rrp6 Δ)*	*rrp47-Δ rrp6-Δ*	*This work
yBD527	*MAT*a *leu2-3,-112 ura3-52 his3-Δ 200 trp1-Δ901 lys2-801 rrp6::URA3 pRrp6-ΔC2 RRP47 TAP:: KANMX4*	*rrp6-ΔC2*	*This work
yBD540	*MAT*a *RRP47-MYC::KAN DIS3-TAP::URA3*	*RRP47-myc Dis3-TAP*	*This work
yBD541	*MAT*a *TAP-RRP41::KAN rrp6::URA3*	*RRP41-TAP rrp6Δ*	*This work
yBD542	*Mata* *ade2-1 can1-100 his3-11,15 leu2-3,112 trp1-1 ura3-1 pep4::HIS3 RRP46-TAP:: kl TRP-rrp6::URA3*	*RRP46-TAP rrp6Δ*	*This work
yBD543	*MAT*a *cyc1-512 ura3-52 trp2-1 HIS3::hisG- DIS3-TAP::kl TRP*	*DIS3-TAP*	*This work

yBD544	*MAT*a *cyc1-512 ura3-52 trp2-1 HIS3::hisG rrp6::URA3 DIS3-TAP:: kl TRP (rrp6-Δ)*	*DIS3-TAP rrp6Δ*	*This work
yBD545	*MAT*a *cyc1-512 ura3-52 trp2-1 HIS3::hisG HIS3-GAL10::protA-DIS3*	*HIS3-GAL10::protA-DIS3*	*This work
yBD122	*MATa leu2-3,112 his3-11,15 ade2-1 ura3-1 trp1-1*	*PRP2*$^{+}$	This work
yBD70	*MAT a ade2-1 his3 ura3 leu2 trp1 prp2-1*	*prp2-1*	(7)
yBD77	*MAT a ade2 his3 ura3 leu2 trp1 prp2-1rrp6::URA3*	*prp2-1 rrp6-Δ*	(7)
yBD608	*Mat*a *W303 Prp4–6HA::NAT-NT2*	*Prp4–6HA*	(14)
yBD614	*Mat*a *W303 Prp4–6HA::NAT-NT2 rrp6::URA3*	*Prp4–6HA rrp6::URA3*	This work

Table S2: List and Descriptions of Plasmids constructed and used

Plasmid no.	Description	Use	Reference
pBD19	*pRS306-pBSKS URA3 amp*^{R}		(8)
pBD 23	*pRS 316 - pBSKS URA3 CEN6 ARS4 amp* ^{R}		(8)
pBD 24	A 1.1 Kb *Hind*III fragment containing the transcribed region of URA3 gene in pUC19.		This work[¥]
pBD 25	A 3.0 kb *Sal*I-*Bam*HI fragment containing 1.1 kb URA3 gene inserted at *Hind*III site of *CBC1* gene flanked by 5′ and 3′ flanking sequence of the same cloned in pUC 19.	*CBC1* Disruptor	(9)
pBD 26	A 3.0 kb fragment carrying 1.1 kb *URA3* gene flanked by 5′ and 3′ flanking sequence of *UPF*1 gene in pBR322.	*UPF1* Disruptor	(1)
pBD32	A 5.5 kb fragment carrying 3.8 kb blaster sequence flanked by 5′ and 3′ flanking sequence of *CBC1* gene in pUC19. *CBC1* blaster.	*CBC1*-Blaster	(1)
pBD42	A 4.1kb fragment containing the *CBC1* (from pBD32) fragment is cloned in pCR 2.1 vector containing 5′and 3′ part of *CBC1* ORF and Blaster.	*CBC1*-Blaster	(1)
pBD 45	A 1.4 kb fragment carrying 1.1 kb *URA3* gene flanked by 5′ and 3′ flanking sequence of *RRP6* gene in pUC19.	*RRP6* Disruptor	(3)
pBD 216	A 3.8Kb blaster fragment from pBD 27(digested with *Bam*HI, *Bgl*II) is cloned into pRS 313 (pBD 20) to get an *HIS3* disruptor. Digestion with *Bgl*I & *Kpn*I gives the final *HIS3* disruptor fragment.	*HIS3* Disruptor	*This work
pBD225	A 2.3 Kb PCR amplified (by OBD 407+ 408) Trf4 fragment from appropriate gene cloned in pJET1.2.	*TRF4* in pJET1.2	*This work
pBD226	A 1.1 Kb *URA3* fragment digested by *Hind*III from pBD24, inserted into *Eco*RI and *Eco*RV site of pBD225.	*TRF4* Disruptor	*This work
pBD 248	A 2.6 Kb *Trf5* fragment (PCR Product by OBD 494+ 495) cloned in pJET1.2. Host DH5α	*TRF5* in pJET1.2	*This work
pBD 254	1.4 Kb *URA3* fragment from *Ssp*I- *Nsi*I site of pRS306 is cloned into *Bst*EII - *Nsi*I site of pBD248. PCR with oBD494 - oBD495 gives the 2.9 Kb *TRF5* Disruptor fragment.	*TRF5* Disruptor	*This work
pBD 266	1.4 Kb *AIR1* fragment (by oBD549+550) in pJET1.2	*AIR1* in pJET1.2	*This work
pBD 285	A 1.6 Kb *URA3* fragment from *Nsi*I - *Xmn*I restriction site of pRS306 cloned into *Nsi*I-*Msc*I restriction site of pBD266 gives *AIR1* disruptor plasmid.	*AIR1* Disruptor	*This work
pBD 320	Rrp6-ΔC2 Plasmid created using Reverse PCR and ligation technique to delete the C-terminal region of *RRP6*	Rrp6-ΔC2	*This work

Table S3. Oligonucleotides used for End Point PCR in this study

OBD NO.	OBD NAME	SEQUENCE (5' to 3')	GENE TO AMPLIFY	REFERENCE
OBD 3	BD-CBC1-D-S1	5'-ACTGTGTAAAGAAATGATGCCC-3'	*CBC1*	(10)
OBD 4	BD-CBC1-D-AS1	5' CGATATCCAATTCAATCTTCGC-3'		
OBD 5	BD-RRP6-D-S1	5' AGAATTTAGACAGGGG-3'	*RRP6*	(3)
OBD 6	BD-RRP6-D-AS1	5' CAT CGTCTCTTCTTGC-3'		
OBD 19	CBC1-BLASTER 5' JUNCTION-S1	5' CGAATGTAGTCCATCCTCCGAATC-3'	*CBC1* BLASTER	(2)
OBD 20	CBC1-BLASTER 5' JUNCTION-AS1	5' TGTGCTCCTTCCTTCGTTCTTCCT-3'		
OBD 25	CBC1-BLASTER 3' JUNCTION-S2	5' TGATGATGACATTCCGGGTCTGGT-3'	*CBC1* BLASTER	(2)
OBD 24	CBC1-BLASTER 3' JUNCTION-AS1	5' CATACCCAACTTTGACTACCTTGC-3'		
OBD 43	AM- RRP4-K-S1	5'-TGACGTATTCCTCTGTTGCTG-3'	*RRP4*	(2)
OBD 44	AM-RRP4-K-AS1	5'-AAAGAGGCACTACCATCTTGAAA-3'		
OBD 53	SD TIF4631 ORF S2	5'-ATGATGGGCGCTATCCTCATCGC-3'	*TIF4631*	(11)
OBD 54	SD TIF4631 ORF AS2	5'-TATCGTAAACGAAGACTGGCGAAGC-3'		
OBD 79	AM-Gal10-Rrp41-Ins-S2	5' TTCCCTGCGTCGCTTGCTGA 3'	*RRP41*	(2)
OBD 80	AM-Gal10-Rrp41-Ins AS2	5'ACCGCCATCTTGCTCAAGGAC 3'		
OBD 146	AM ACT1-Forward	5' TGACGACGCTCCTCGTGCTG 3'	*ACT1*	(2)
OBD177	AM ACT1-Reverse	5'- GGCAACTCTCAATTCGT -3'		
OBD 254	SD-UPF1-Forward	5'-ATTTTAGTATCATCAGTTTCC-3'	*UPF1*	(11)
OBD 255	SD-UPF1-Reverse	5'-CAACAAGTATAACTTGTTTCG-3'		
OBD 362	AM_trf4_S1 .	5'ACCTGTATTCATCCTCGGCTTCTTGTAT ATGATTCTAAAATAATGTGTGAAAAAAA AAATTTGATTCGGTAATCTCCGAGCAG-3'	*TRF4*	(2)
OBD 363	AM_trf4_AS1 .	5'ACACATTCTATCCAGGTACACAGTGAT GTACAGTTCAGTGCATCATTTAAACAAA AAAGGCTAATTTGTGAGTTTAGTATACA TGCATTTACTT-3'		
OBD 405	FORWARD:AC_air1_S1	5'GGCCAAGGACGGTATTTTGG-3'	*AIR1*	*This work
OBD 406	REVERSE:AC_air1_AS1	5'CACATGCAAGACGTAGACCCA-3'		
OBD 407	FORWARD:AC_trf4_S1	5'AAGTAAAGGATCCCAAGCGTGA-3'	*TRF4*	(2)
OBD 408	REVERSE:AC_trf4_AS1	5'TTCGGCAAACGTGCCAATTT-3'		

OBD NO.	OBD NAME	OBD SEQUENCE (5' to 3')	GENE TO AMPLIFY	REFERENCE
OBD 419	AC_HIS_Del S1	5’-TTGGCCTCCTCTAGTACACTCT-3’	*HIS3*	*This work
OBD 420	AC_HIS_Del AS1	5’-CAACCGCAAGAGCCTTGAAC-3’		
OBD 425	AC_trf4 dsrptor_S2	5'-GTGCCGACATTTGAGGAGGA-3'	*TRF4*	(2)
OBD 426	AC_trf4 dsrptor_AS2	5'-CGCTAAGGGCAGCGATTCTA-3'		
OBD 429	AC_trf4 dsrptor_S1	5’-AGTCTGCACAAGACCCTTCG-3’	*TRF5*	(2)
OBD 430	AC_trf4 dsrptor_AS1	5’-ACGTTACGATAGAGCCAGCG-3’		
OBD 494	AM-TRF5-S1	5’-GCTAACACCACCACCCATGA-3’	*TRF5*	*This work
OBD 495	AM-TRF5-AS1	5’-GGGAAAATACCCACGCGTTT-3’		
OBD 677	AC_RRP6_DEL_F1_	TTATGTAAAACAAGCGTATTTTTTTATT<br>TAT	*RRP6*	*This work
OBD 678	AC_RRP6_DEL_R1	TGGAATGGGAGTAGCTTCCT		
OBD 679	AC_RRP6_DEL_R2	TGGAATGGGAGTAGCTTC		
OBD 772	AC_Rrp6_DelC2_Check_F	CATGTCCGCCAGAATGCAAA	Rrp6-ΔC2	*This work
OBD 773	AC_Rrp6_Del C2_Check_R	GCCATAACTCCATGACACAGAT		
OBD 789	AC-DIS3-HIS3-FORWARD	AATATGAATATATCCTTTTGAACTGGA<br>GTGAATGGTGGGAAATCTCAAATATTT<br>TCTGAAATAGGACCCACACAAGATATT<br>GAAAGTAAG<br>CTCTTGGCCTCCTCTAG	*DIS3*	*This Work
OBD 790	AC-DIS3-GAL10-REVERSE 1	ATGCTTACCAATGGGTGCGCTTAATTCT<br>AAGGGAGAATCTGACAAGATGAACTTC<br>GGCAACTCATTTTGAGCATCGGGGACG<br>ACAATTT<br>GAATTTTCAAAAATTCTTACTTTT		
OBD 791	AC-DIS3-GAL10-REVERSE 2	TGTCGTCCCCGATGCTCAAAATGAGTT<br>GCCGAAGTTCATCTTGTCAGATTCTCCC<br>TTAGAATTAAGCGCACCCATTGGTAAG<br>CATTA<br>GAATTTTCAAAAATTCTTACTTTT		

Table S4: Oligonucleotides used for qRT PCR in this study

OBD NO.	OBD NAME	OBD SEQUENCE (5' to 3')	GENE TO AMPLIFY	REFERENCE
OBD 168	AM -SCR1-S2	5' TTGTGGCAACCGTCTTTCCT 3'	*SCR1*	*This work
OBD 169	AM-SCR1-AS2	5' CCGAAGCGATCAACTTGCAC 3'		
OBD 268	US-ACT1-RT-S1	5'-GCCGAAAGAATGCAAAAGGA-3'	ACT1	*This work
OBD 269	US-ACT1-RT-AS1	5'-TCTGGAGGAGCAATGATCTTGA-3'		
OBD 343	AM-RT-5s_S1	5'-GGTTGCGGCCATATCTACCA-3'	MATURE 5S	*This work
OBD 344	AM-RT-5s_AS1	5'-ACTACTCGGTCAGGCTCTTACCA-3'		
OBD 188	AM-5.8S-RT-S1-	5'-AAC AAC GGA TCT CTT GGT TCT-3'	MATURE 5.8S	*This work
OBD 189	AM-5.8S-RT-AS1	5'-AAA TGA CGC TCA AAC AGG CA-3'		
OBD 341	AM-RT-18s_S2	5'-GATCGGGTGGTGTTTTTTTAATG-3'	MATURE 18S	*This work
OBD 342	AM-RT-18s_AS2	5'-CTCCCCCCAGAACCCAAA-3'		
OBD 553	AC_25s_RT_S1	5'-GGACTGAGGACTGCGACGTAA -3'	MATURE 25S	*This work
OBD 554	AC_25s_RT_AS1	5'- TCAAGACGGGCGGCATAT -3'		
OBD 578	AC_snRNA U1_ S1	5'-ACGGCAGATTCGAATGAACTTAA-3'	snRNA U1	*This work
OBD 579	AC_snRNA U1_ AS1	5'-TACAATCCCGACCAAATAATCTCA-3'		
OBD 580	AC_snRNA U4_ S1	5'-AGGATTCGTCCGAGATTGTGTT-3'	snRNA U4	*This work
OBD 581	AC_snRNA U4_ AS1	5'-CATGAGGAGACGGTCTGGTTTAT-3'		
OBD 582	AC_SNR 10_S1	5'-CGATCTTGGGTGCAACAGTCT-3'	SNR 10	*This work
OBD 583	AC_SNR 10_AS1	5'-TCATCCGGGCACACGAA-3'		
OBD 584	AC_SNR 13_S1	5'-TGAGTGCATTTGGCTCGAGTT-3'	SNR 13	*This work
OBD 585	AC_SNR 13_AS1	5'-GCTTGAGTTTTTCCACACCGTTA-3'		
OBD 600	AC_snRNA U6_S1	5'-TCGTGGACATTTGGTCAATTTGA-3'	snRNA U6	*This work
OBD 601	AC_snRNA U6_AS1	5'-TTTGTAAAACGGTTCATCCTTATGCA-3'		
OBD 638	AC_pre 5s_S1	5'-GGAAACGGTGCTTTCTGGTAGA-3'	PRE 5S	*This work
OBD 639	AC_pre 5s_AS1	5'-ATCACCTGCGTTTCCGTTAAA-3'		
OBD 640	AC_pre 18s_S1	5'-AGTCGTAACAAGGTTTCCGTAGGT-3'	PRE 18S	*This work
OBD 641	AC_pre 18s_AS1	5'-CTTGCCAAAACAAAAAAATCCAT-3'		
OBD 642	AC_pre 5.8s_S1	5'-CATCGAATCTTTGAACGCACAT-3'	PRE5.8S	*This work
OBD 643	AC_pre 5.8s_AS1	5'-GGCCAGCAATTTCAAGTTAACTC-3'		
OBD 644	AC_pre 5.8s Margin_S1	5'-CATCGAATCTTTGAACGCACAT-3'	PRE 5.8S II	*This work
OBD 645	AC_pre 5.8s Margin_AS1	5'-AGAAGGAAATGACGCTCAAACAG-3'		
OBD 646	AC_pre 25s_S1	5'-CCTTGTTGTTACGATCTGCTGAGA-3'	PRE 25S	*This work
OBD 647	AC_pre 25s_AS1	5'-TGCCAGTACCCACTTAGAAAGAAATAA-3'		

OBD 658	AC-ITS1-5.8s-S1	5'-CTGTGGAGTTTTCATATCTTTGCAA-3'	*ITS1*	*This work
OBD 659	AC-ITS1-5.8s-AS1	5'-TACCTCTGGGCCCCGATT-3'		
OBD 677	AC_RRP6_DEL_F1_	5'-TTATGTAAAACAAGCGTATTTTTTTATTTAT-3'	*RRP6*	*This work
OBD 678	AC_RRP6_DEL_R1	5'-TGGAATGGGAGTAGCTTCCT-3'		
OBD 679	AC_RRP6_DEL_R2	5'-TGGAATGGGAGTAGCTTC-3'		
OBD 680	AC pre U1 Forward	AACGGGTGGATCTTATAATTTTTGA	snRNA U1	*This work
OBD 681	AC pre U1 Reverse I	AGAAGCATGAAACTTTAAAAGTTTCAGTAC		
OBD 682	AC pre U1 Reverse II	AAACACATCACCAGATATAGGACTGAA		
OBD 683	AC_ pre U1 Reverse III	TCTCAATGTAGCGTATAACAGGTTTCA		
OBD 684	AC_pre U4 F	TCGGTGTTCGCTTTTGAATACTT	snRNA U4	*This work
OBD 685	AC_pre U4 R I	CCATTTTAGTTGCCATTTCTGTATTACTT		
OBD 686	AC_pre U4 R II	GTGAACTCACATTAGAGAATTCGG		
OBD 687	AC_pre U4 R III	CAATTCCAGCTTCTATAACGTGAAC		
OBD 688	AC_ pre U6 Forward	CAGTTCCCCTGCATAAGGATGA	snRNA U6	*This work
OBD 689	AC_ pre U6 Reverse	GTCACGATACTTCACTCGATGATAAAAA		
OBD 690	AC_ pre SNR13 Forward	GAGTTGCTGTTTGGCTTTTGC	PRE snR 13	*This work
OBD 691	AC_ pre SNR13 Reverse	ATTTTCTACGGGGAAGTTAAAAGGTC		
OBD 692	AC_ pre SNR13 Reverse II	AGCGCTACGATACAATGTAAGAAGG		
OBD 698	AC_RRP47 TAP Forward:	TAAGGAAAGCAAGTAGTAAGAAAAGTAAAAG ATTGGATAAAGTTGGAAAAAAGAAAGGAGGG AAGAAGTCATCCATGGAAAAGAGAAG	*RRP47*	*This work
OBD 699	AC_RRP47 TAP Reverse:	ATTTTTGCATTTGTGCTCTCACATCACCTTTAA TCATTTTTTCACTCATGTACCAGTATACGTCGA CCTTACGACTCACTATAGG		
OBD 700	AC_RRP6 TAP_Forward:	TAGTAATGGACCAAGGGCAGCTAAAAAGAGG AGGCCTGCCGCCAAAGGTAAGAATCTGTCATT TAAAAGGTCATCCATGGAAAAGAGAAG	*RRP6*	*This work
OBD 701	AC_RRP6 TAP_Reverse:	GATGAATTTAGAGGTCTTAAATGAAAATTACC ATAATTTATAAATAAAAAAATACGCTTGTTTTA CATAATACGACTCACTATAGGG		
OBD 702	AC_5s R1:	GGCTCTTACCAGCTTAACTACAGTTGA	MATURE 5S	*This work
OBD 703	AC_5s F2:	GGTAAGAGCCTGACCGAGTAGTG		
OBD 704	AC_5s R2:	AAAGATTGCAGCACCTGAGTTTC		
OBD 705	AC_pre 5s F2:	GGCTTCCTATGCTAAATCCCATAAC	PRECURSOR 5S	*This work
OBD 706	AC_pre 5s R2:	GCAGCTGGATAGTGCGAATTTT		
OBD 707	AC_5.8s R2:	GGCGCAATGTGCGTTCA	MATURE 5.8S	*This work
OBD 708	AC_5.8s F3:	CATCGAATCTTTGAACGCACAT		
OBD 709	AC_5.8s F4 MARGIN:	CGTCATTTCCTTCTCAAACATTCTG		
OBD 710	AC_Pre 5.8s R4:	GGCCAGCAATTTCAAGTTAACTC	PRECURSOR 5.8S	*This work

OBD 711	AC_Pre 5.8s F5:	GGCCTTTTCATTGGATGTTTTT		
OBD 712	AC_Pre 5.8s R5:	AAACGACCGTACTTGCATTATACCT		
OBD 713	AC_Pre 5.8s F6:	CTGCGGCTAATCTTTTTTTATACTGA		
OBD 714	AC_Pre 5.8s R6:	GTTCGCCTAGACGCTCTCTTCTTA		
OBD 715	AC_U1 F2:	TCCTTGGTCACACACACATACG	snRNA U1	*This work
OBD 716	AC_U1 R2:	GGGAATGGAAACGTCAGCAA		
OBD 717	AC_Pre U1 F III:	TGCTTCTATTTTCTTCATTTCAGTCCTA	PRE snRNA U1	*This work
OBD 718	AC_Pre U1 F4:	TACGCTACATTGAGACAAGACATTGT		
OBD 719	AC_Pre U1 R4:	TGCGGCTCTCTTAAGCACATT		
OBD 720	AC_Pre U4 F2:	TTAGGGATACCGGACTGAAACATT	PRE snRNA U4	*This work
OBD 721	AC_Pre U4 F3:	CGAATTCTCTAATGTGAGTTCACGTT		
OBD 722	AC_Pre U4 F4:	TGTTCTCCTTCTTGTGTGTCTTTCTT		
OBD 723	AC_Pre U6 F2:	ACCCAGCGTACAGCAGTGTATCT	PRE snRNA U6	*This work
OBD 724	AC_Pre U6 R2:	TCGAACGCGAGACAATTTTCTA		
OBD 725	AC_snR10 F2:	GCCATTCGTAACACGTACAGTATCTC	MATURE snR 10	*This work
OBD 726	AC_snR10 R2:	CCACATTCTTCATGGGTCAAGA		
OBD 727	AC_Pre snR10 F1:	TGTATATATGAGTGGCTTGGAATGC	PRE snR 10	*This work
OBD 728	AC_Pre snR10 R1:	AAATTGCGTCAACCGAAGGA		
OBD 729	AC_Pre snR13 F2:	TTCCCCGTAGAAAATCTTAGTAATCC	PRE snR 13	*This work
OBD 730	AC_Pre snR13 R3:	GCCAAACCCAACGTACTAACATC		
OBD 731	AC_Pre snR13 F3:	GCGCTGCATATATAATGCGTAAAAT		
OBD 732	AC_U2 F1:	TGGCACCCAAAATAATAAAATGG	snRNA U2	*This work
OBD 733	AC_U2 R1:	TGAGACCTGACATTAGCGGAAA		
OBD 734	AC_U2 F2:	CCCCAAGTATCGGCCAAAGT	snRNA U2	*This work
OBD 735	AC_U2 R2:	ACCCTACACCCCCTCAAACC		
OBD 736	AC_U2 F3:	CCGGCGGCATCAAGAA	snRNA U2	*This work
OBD 737	AC_U2 R3:	AGGGTCGCGACGTCTCTAACT		
OBD 738	AC_Pre U2 F4:	TTTTTCCTTTGACTTCGCATGA	pre snRNA U2	*This work
OBD 739	AC_Pre U2 R4	TTTGGTTGCGTGGTATATAGAATCTC		
OBD 740	AC_Pre U2 F5	TGGCGGCATACTCTTTCTTGA	pre snRNA U2	*This work
OBD 741	AC_Pre U2 R5	AGGCAATGGGAAGCAGCTAA		
OBD 742	AC_U5 F1	GGGAGGTCAACATCAAGAACTGT	snRNA U5	*This work
OBD 743	AC_U5 R1	GATGGTTCTGGTAAAAGGCAAGA		
OBD 744	AC_U5 F2	TCCGGGTGTTGTCTCCATAGA	snRNA U5	*This work
OBD 745	AC_U5 R2	AGGGCAGAAAAGTTCCAAAAAAT		
OBD 746	AC_Pre U5 F3	GGAGGGCGTTTATCTTTTCTATTTTATT	pre snRNA U5	*This work
OBD 747	AC_Pre U5 R3	CATGGACTCATGAATCAAATTTGTAGA		

OBD 748<br>OBD 749	AC_Pre U5 F4<br>AC_Pre U5 R4	GAGTCCATGGAACAAATATATAGAACTCA<br>GCAGTCAGGATAAAAGCAAATGC	pre-snRNA U5	*This work
OBD 758<br>OBD 759	AC_RRP4_RT_S1<br>AC_RRP4_RT_AS1	5' TCGAACAACATACGACAAGCAAT 3'<br>5' CAGAACGCTAACGCTTTGATCA 3'	*RRP4*	*This work
OBD 760<br>OBD 761	AC_RRP6_RT_S1<br>AC_RRP6_RT_AS1	5' TCAAGAACACCAATGAGGAAGCT 3'<br>5' TCCAACAATATTCCGTCTGCTTT 3'	*RRP6*	*This work
OBD 762<br>OBD 763	AC_MTR4_RT_S1<br>AC_MTR4_RT_AS1	5' TTGCATTCCAAGAACGCTGTA 3'<br>5' TCCAACAATATTCCGTCTGCTTT 3'	*MTR4*	*This work
OBD 772<br>OBD 773	AC_Rrp6_DelC2_Check_F<br>AC_Rrp6_DelC2_Check_R	CATGTCCGCCAGAATGCAAA<br>GCCATAACTCCATGACACAGAT	*RRP6*-ΔC2	*This work
OBD 800<br>OBD 801	AC_DIS3_RT_FORWARD<br>AC_DIS3_RT_REVERSE	5' TGGCGATGACGATGACAATAA 3'<br>5' GCGCTGCTTGTCGGAAAT 3'	*DIS3*	*This work
OBD 802<br>OBD 803	AC_RRP41_RT_FORWARD<br>AC_RRP41_RT_REVERSE	5' TACTCGCCAGAAGGGCTACGT 3'<br>5' GCGGATGTGTGTTGATGGAA 3'	*RRP41*	*This Study
OBD 804<br>OBD 805	AC_RRP46_RT_FORWARD<br>AC_RRP46_RT_REVERSE	5' CGGCATTTTTGCCTTTAGTTG 3'<br>5' TAGGGATGCTTGCGCACATA 3'	*RRP46*	*This Study
OBD 810<br>OBD 811	5.8S L 5' F_<br>5.8S+30 3' R_	AATATTAAAAACTTTCAACAACGGATCTCT<br>ACTCACTACCAAACAGAATGTTTGA	5.8S<br>pre 5.8S	*This Study (used for both End-point and RT-qPCR)

Table S5: Sequences of the oligonucleotide probes for specific ncRNAs used in the Northern Blot Analysis

Sl. No.	RNA	Sequence	References
1.	**5S rRNA**	5′-DIG/5DigN/ACTACTCGGTCAGGCTCTTA-3′	This work
2.	**5.8S rRNA**	5'-DIG/5DigN/TGCGTTCTTCATCGATGCGAGAACC-3′	(3)
3.	**U1 snRNA**	5'-DIG/5DigN/GTATGTGTGTGTGACCAAGGA-3′	This work
4.	**snR10 snoRNA**	5'-DIG/5DigN/GTTCTCCAGTCCAAGCCAATAA-3′	This work
5.	***SCR1***	5'-DIG/5DigN/AAGGACCCAGAACTACCTTG-3′	(12)
6.	***ACT1***	5'-DIG /5DigN/CAGTGGTGGAGAAAGAGTAACC-3′	(12)

Table S6: Sequences of the oligonucleotide primers used for LM-PAT Analysis in this study

OBD NO.	NAME	SEQUENCE (5' to 3')	GENE TO AMPLIFY	REFERENCE
OBD 392	Anchor primer	5'-GCGAGCTCCGCGGCCGCGTTTTTTTTTTTT-3'	Anchor Specific Primer	(13)
OBD 343	5S RNA Sense 5′-End	5'-GGTTGCGGCCATATCTACCA-3	5S	This work
OBD 856	5S RNA AS 3′-End	5’ AGATTGCAGCACCTGAGTTTC 3’		
OBD 188	5.8S RNA Sense 5′-End	5’-AACAACGGATCTCTTGGTTCT-3’	5.8S	This work
OBD 189	5.8S RNA AS 3′-End	5’-AAATGACGCTCAAACAGGCA-3’		
OBD 857	U1 snRNA Sense 5′-End	5’-ACTTACCTTAAGATATCAGAGGAGA-3’	U1 snRNA	This work
OBD 858	U1 snRNA AS 3′-End	5’-AAATCAAAAATTATAAGATCCACCC-3’		
OBD 859	snR10 Sense 5′-End	5’-AACGCAAATTTAACAGCCATTC-3’	snR10	This work
OBD 860	snR10 AS 3′-End	5’- GTTGTATTATCAATCCTTGCAACG-3’		

Table S7: Different Primary antibody used for the work and their respective secondary Antibody

Sl. No.	Primary Antibody	Source	1º Ab Dilution	Secondary Antibody	2º Ab Dilution
1	Anti-Rrp6	Dr. Scott Butler, University of Rochester	1:1000		
2	Anti-TAP	Commercially Procured, Thermo Scientific	1:1000	Anti-Rabbit	1:3000
3	Anti-Rrp4	Dr. Scott Butler, University of Rochester	1:1500		
4	Anti-Mtr4	Dr. Scott Butler, University of Rochester	1:1000		
5	Anti-myc	Dr. Suvendra Nath Bhattacharyya, CSIR-IICB, India	1:500	Anti-Mouse	1:3000
6.	Anti-β Tubulin	Commercially Procured, Hind Biotech India	1:2000		
	Anti-β HA	Commercially Procured, Biobharati Life Science, India	1:1000	Anti-Mouse	1:3000
7.	Anti-Prp8p	Commercially Procured, Biobharati Life Science, India	1:1000	Anti-Mouse	1:3000
	Anti-Prp40p	Commercially Procured, Biobharati Life Science, India	1:1000	Anti-Mouse	1:3000
	Anti-Spp381p	Commercially Procured, Biobharati Life Science, India	1:1000	Anti-Mouse	1:3000
	Anti-Nrd1p	Dr. Steven Buratowski, Harvard University	1:1000	Anti-Mouse	1:3000

Table S8: Sequence Data of Various LM-PAT Clones, Specific Sites and Length of Poly(A) Tails

**Sequences of 5S LM-PAT Clones**	**Polyadenylation sites**	**No. of Clones Analyzed**	**Length of Poly(A) Tract**
GCAAATATTCGTCGATCACTGTAGTTAAGCTGGTAAGAGCCTGACCGAGTAGTGTAGTGGGT<br>GACCATACGCGAAACTCAGGTGCTGCAATCTTTATTTCTTTTAAAAAAAAAAAA	+11	6	12A
GTCAAATCGTCGATCACTGTAGTTAAGCTGGTAAGAGCCTGACCGAGTAGTGTAGTGGGTGA<br>CCATACGCGAAACTCAGGTGCTGCAATAAAAAAAAAAAAAA	-2	6	14A
GTCGATTCCGTCGATCACTGTAGTTAAGCTGGTAAGAGCCTGACCGAGTAGTGTAGTGGGTG<br>ACCATACGCGAAACTCAGGTGCTGCAATAAAAAAAAAAAAAAAAAAAAAAAAAAAAAAAAAA<br>AAAAAAAAAAAAAAAAAAAAAAAAAAAAAAAAAAAAAAAAAAAAAAAAAAAAA	-2	1	87A
GACGAAATATCGTCGACAACTGTAGTTAAGCTGGTAAGAGCCTGACCGAGTAGTGTAGTGGG<br>TGACCATACGCGAAACTCAGGTGCTGCAAAAAAAAAAAAAACAAAAAAAAAAA	-3	5	26A
GACGAAATATCGTCGACAACTGTAGTTAAGCTGGTAAGAGCCTGACCGAGTAGTGTAGTGGG<br>TGACCATACGCGAAACTCAGGTGCTGCAAAAAAAAAAAAAACAAAAAAAAAAAAAAAAAAAA<br>AAAAAAAAAAAAAAAAAAAAAAAA	-3	2	52A

TOTAL NUMBER OF CLONES ANALYZED: 20

**Sequences of 5.8S LM-PAT Clones**	**Polyadenylation sites**	**No. of Clones Analyzed**	**Length of Poly(A) Tract**
AAACTTTCAACAACGGATCTCTTGGTTCTCGCATCGATGAAGAACGCAGCGAAATGCGATAC<br>GTAATGTGAATTGCAGAATTCCGTGAATCATCGAATCTTTGAACGCACATTGCGCCCCTTGG<br>TATTCCAGGGGGCATGCCTGTTTGAGCGTCATTTCAAAAAAAAAAAA	+1	4	12A
AAACTTTCAACAACGGATCTCTTGGTTCTCGCATCGATGAAGAACGCAGCGAAATGCGATAC<br>GTAATGTGAATTGCAGAATTCCGTGAATCATCGAATCTTTGAACGCACATTGCGCCCCTTGG<br>TATTCCAGGGGGCATGCCTGTTTGAGCGTCATTTCAAAAAAAAAA	+1	4	10A
AAACTTTCAACAACGGATCTCTTGGTTCTCGCATCGATGAAGAACGCAGCGAAATGCGATAC<br>GTAATGTGAATTGCAGAATTCCGTGAATCATCGAATCTTTGAACGCACATTGCGCCCCTTGG<br>TATTCCAGGGGGCATGCCTGTTTGAGCGTCATTTCCTTCTCAAACATTCTGTTTGGTAGAAA<br>AAAAAAAAAAAAA	+25	6	16A
AAACTTTCAACAACGGATCTCTTGGTTCTCGCATCGATGAAGAACGCAGCGAAATGCGATAC<br>GTAATGTGAATTGCAGAATTCCGTGAATCATCGAATCTTTGAACGCACATTGCGCCCCTTGG<br>TATTCCAGGGGGCATGCCTGTTTGAGCGTCATTTCCTTCTCAAACATTCTGTTTGGTAAAAA<br>AAAAAAAAAAAAAAAA	+24	1	19A
AAACTTTCAACAACGGATCTCTTGGTTCTCGCATCGATGAAGAACGCAGCGAAATGCGATAC<br>GTAATGTGAATTGCAGAATTCCGTGAATCATCGAATCTTTGAACGCACATTGCGCCCCTTGG<br>TATTCCAGGGGGCATGCCTGTTTGAGCGTCATTTCCTTCTCAAACATTCTGTTTGGTAAAAA<br>AAAAAAAA	+24	2	12A
AAACTTTCAACAACGGATCTCTTGGTTCTCGCATCGATGAAGAACGCAGCGAAATGCGATAC<br>GTAATGTGAATTGCAGAATTCCGTGAATCATCGAATCTTTGAACGCACATTGCGCCCCTTGG<br>TATTCCAGGGGGCATGCCTGTTTGAGCGTCATTTCCTTCTCAAACATTCTGTTTGGTAGTGA<br>AAAAAAAAAAAA	+28	3	12A

TOTAL NUMBER OF CLONES ANALYZED: 20

Supplementary Figures

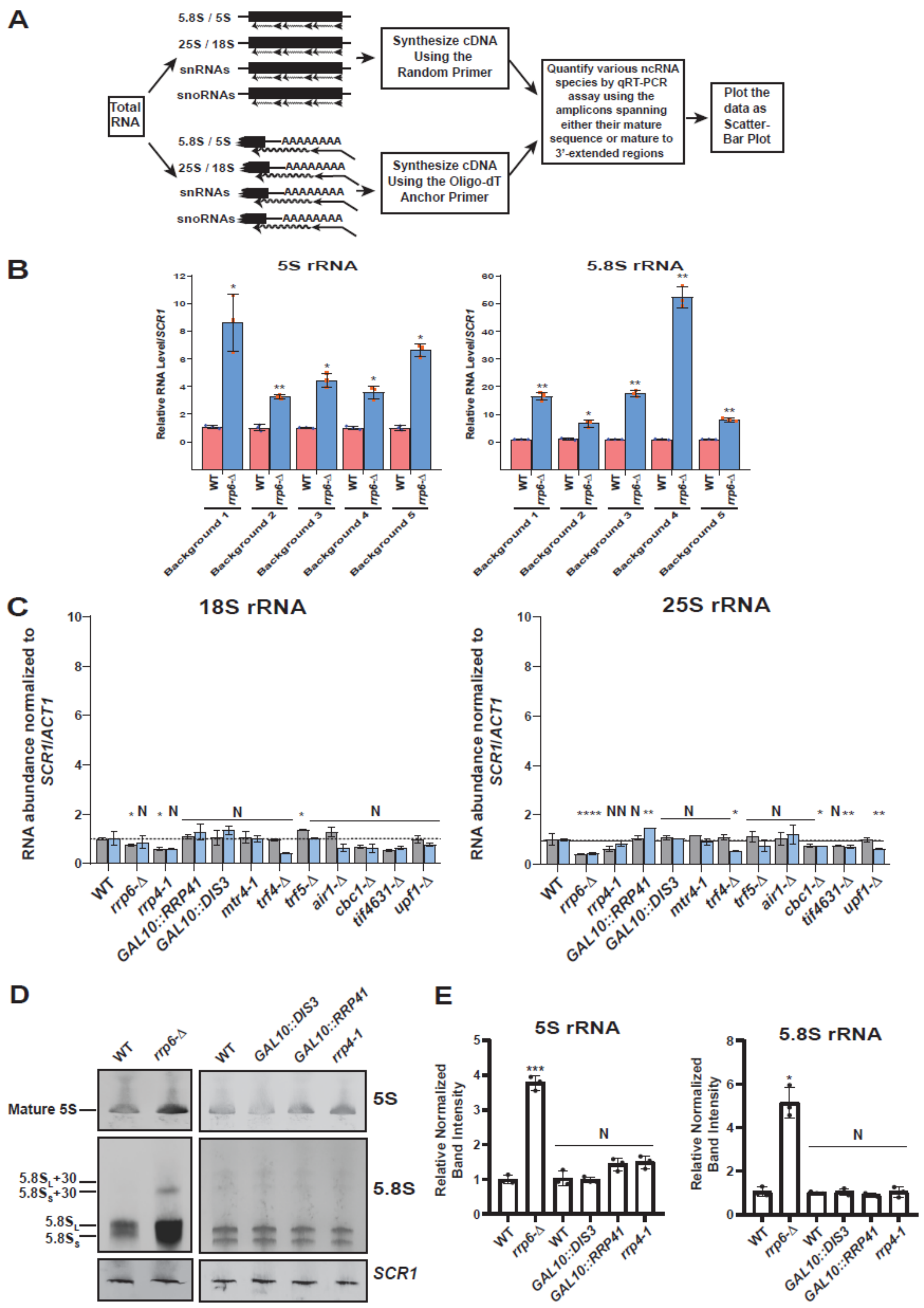

Supplementary Figure S1: (A) Schematic diagram showing the workflow of the experimental approach undertaken in the present work to detect the abundance of the various non-coding RNA species in WT and different mutant yeast strains by RT-qPCR assay using either random hexanucleotide primers or oligo-dT_{30} primer. (B) Bar plots revealing the normalized (to *SCR1* RNA) steady-state levels of the 5S and 5.8S rRNAs estimated from 2 ng cDNA samples from

indicated isogenic WT (BG1: yBD-5, BG2: yBD-263, BG3: yBD-117, BG4: yBD-161, BG5: yBD-65) and *rrp6*-Δ (BG1: yBD-12, BG2: yBD-265, BG3: yBD-129, BG4: yBD-162, BG5: yBD-421) strains with diverse genetic backgrounds using random primers. The genotypes of these strains are listed in Supplementary Table S1. Normalized values of each of the ncRNAs in the wild-type yeast strain were set to one. (C) Bar plots revealing the steady-state levels of 18S and 23S rRNAs estimated from 2 ng cDNA samples prepared using either random hexanucleotide primers (grey bars) or oligo-dT_{30} anchor primer (blue bars) by RT-qPCR assay with the amplicons corresponding to mature regions of these RNAs from the WT (yBD-263) strain and strains carrying mutations in the components of the nuclear exosome, *rrp4-1* (yBD-285), *GAL10::RRP41* (yBD-298), *GAL10::DIS3* (yBD-545); TRAMP, *mtr4-1* (yBD-66), *trf4*-Δ (yBD-306), *trf5*-Δ (yBD-334), and *air1*-Δ (yBD-458); and CTEXT, *cbc1*-Δ (yBD-264), and *tif4631*-Δ(yBD-266) complexes. The *upf1*-Δ yeast strain (yBD-300) was used as a negative control. *SCR1* (in the case of Random Primer) and *ACT1* mRNA (in the case of oligo dT_{30} Primer) were used as the internal control for normalization. Normalized values of each of the ncRNAs in the wild-type yeast strain were set to one. Three to four independent cDNA preparations (biological replicates, n = 3 and in few cases 4) were used to determine the levels of various ncRNAs. The statistical significance of difference reflected in the ranges of P values estimated from Student's two-tailed t-tests for a given pair of test strains for each message are presented with the following symbols, *<0.05, **<0.005, and ***<0.001; N, not significant. (D) Northern blots revealing the steady-state levels of 5S and 5.8S rRNAs in various yeast strains. Total RNA samples isolated from isogenic WT (yBD-263), *GAL10::DIS3* (yBD-545), *GAL10::RRP41* (yBD-298), *rrp4-1* (yBD-285), and *rrp6*-Δ (yBD-265) strains were separated on a 15% denaturing acrylamide gel and analyzed further by northern blotting using DIG-labelled oligonucleotide probes corresponding to the mature regions of these rRNAs as described in materials and methods (See Supplementary Table S5 for their sequence). *SCR1* RNA was used as a loading control. (D) Quantification of northern hybridization data for 5S and 5.8S rRNA from panel D. Individual rRNA levels were normalized to corresponding to *SCR1* RNA signal. Normalized values of each rRNA in the wild-type yeast strain were set to one.

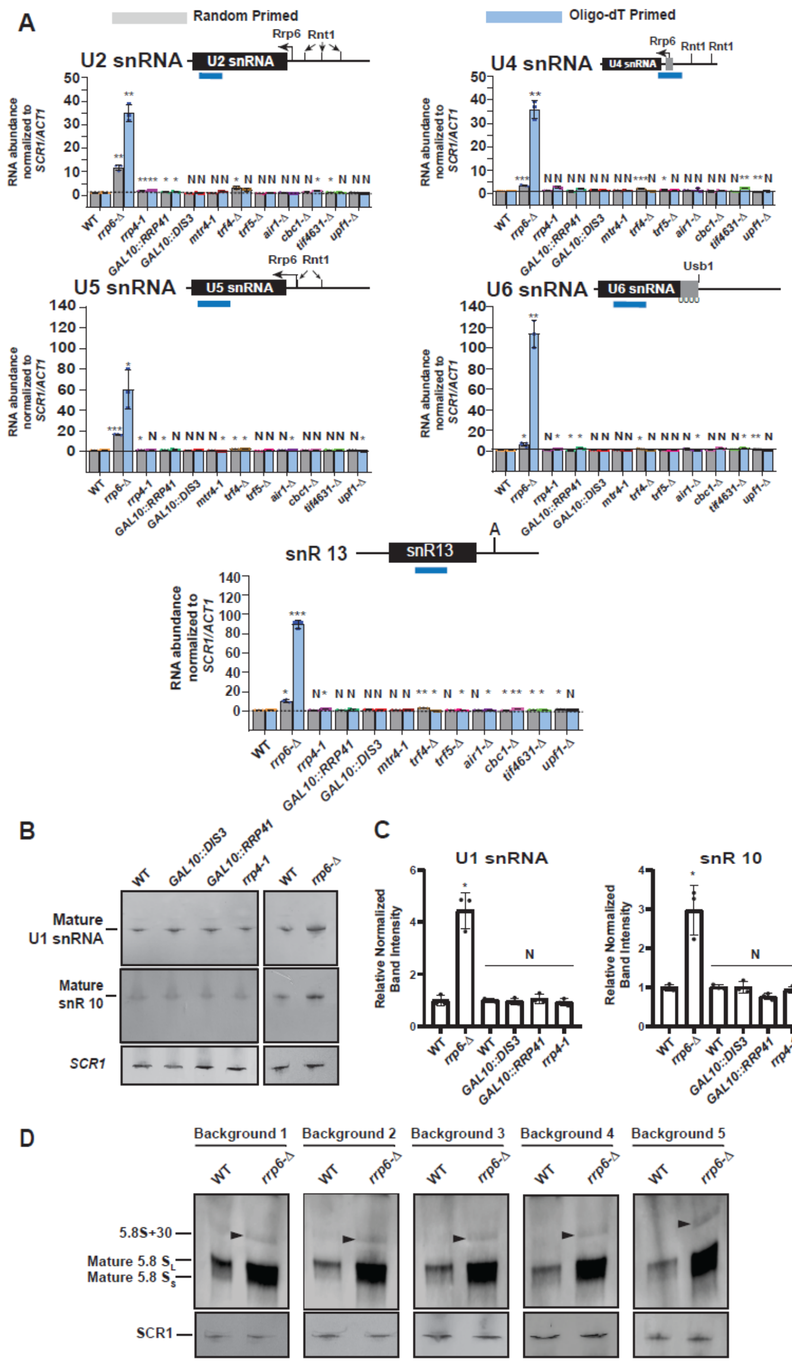

Supplementary Figure S2: Polyadenylated versions of various snRNAs and snoRNAs accumulate in an *rrp6*-Δ yeast strain. (A) Bar plots revealing the steady-state levels of various snRNAs and snoRNAs estimated from the 2 ng cDNA samples from the indicated isogenic yeast strains using either random hexanucleotide primers (grey bars) or oligo-dT$_{30}$ anchor primer (blue bars) by RT-qPCR assay with the amplicons corresponding to their mature sequence (shown on top of each graph). The *upf1*-Δ yeast strain (yBD-300) was used as a negative control. The sites for Rrp6p processing and Rnt1p cleavage downstream of each ncRNA gene are indicated. *SCR1* (in the case of Random Primer) and *ACT1* mRNA (in the case of oligo dT Primer) were used as the internal control. Normalized values of each of the ncRNAs in the WT yeast strain were set to one. Three to four independent cDNA preparations

(biological replicates, n = 3 and in some cases 4) were used to determine the levels of various ncRNAs. The statistical significance of difference reflected in the ranges of P values are presented with the following symbols, *<0.05, **<0.005, and ***<0.001; N, not significant. (B) Northern blots revealing the steady-state levels of U1 snRNA and SNR10 RNAs in various yeast strains. Total RNA samples isolated from indicated yeast strains were separated on a 15% denaturing acrylamide gel and analyzed further by northern blotting using DIG-labelled oligonucleotide probes corresponding to the mature regions of these RNAs as described in materials and methods (See Supplementary Table S5 for their sequence). *SCR1* RNA was used as a loading control. (C) Quantification of northern hybridization data for U1 and snR10 RNA from panel C. Individual RNA levels were normalized to corresponding to SCR1 RNA signal. Normalized values of each rRNA in the wild-type yeast strain were set to one. (D) Northern blots revealing the steady-state levels of 5.8S rRNAs in the prepared from isogenic WT (BG1: yBD-5, BG2: yBD-263, BG3: yBD-117, BG4: yBD-161, BG5: yBD-65) and *rrp6*-Δ (BG1: yBD-12, BG2: yBD-265, BG3: yBD-129, BG4: yBD-162, BG5: yBD-421) strains with various genetic backgrounds. *SCR1* RNA was used as a loading control. Position of mature and precursor species are indicated on the left of the panel and arrowheads indicate the 5.8S+30 precursor band.

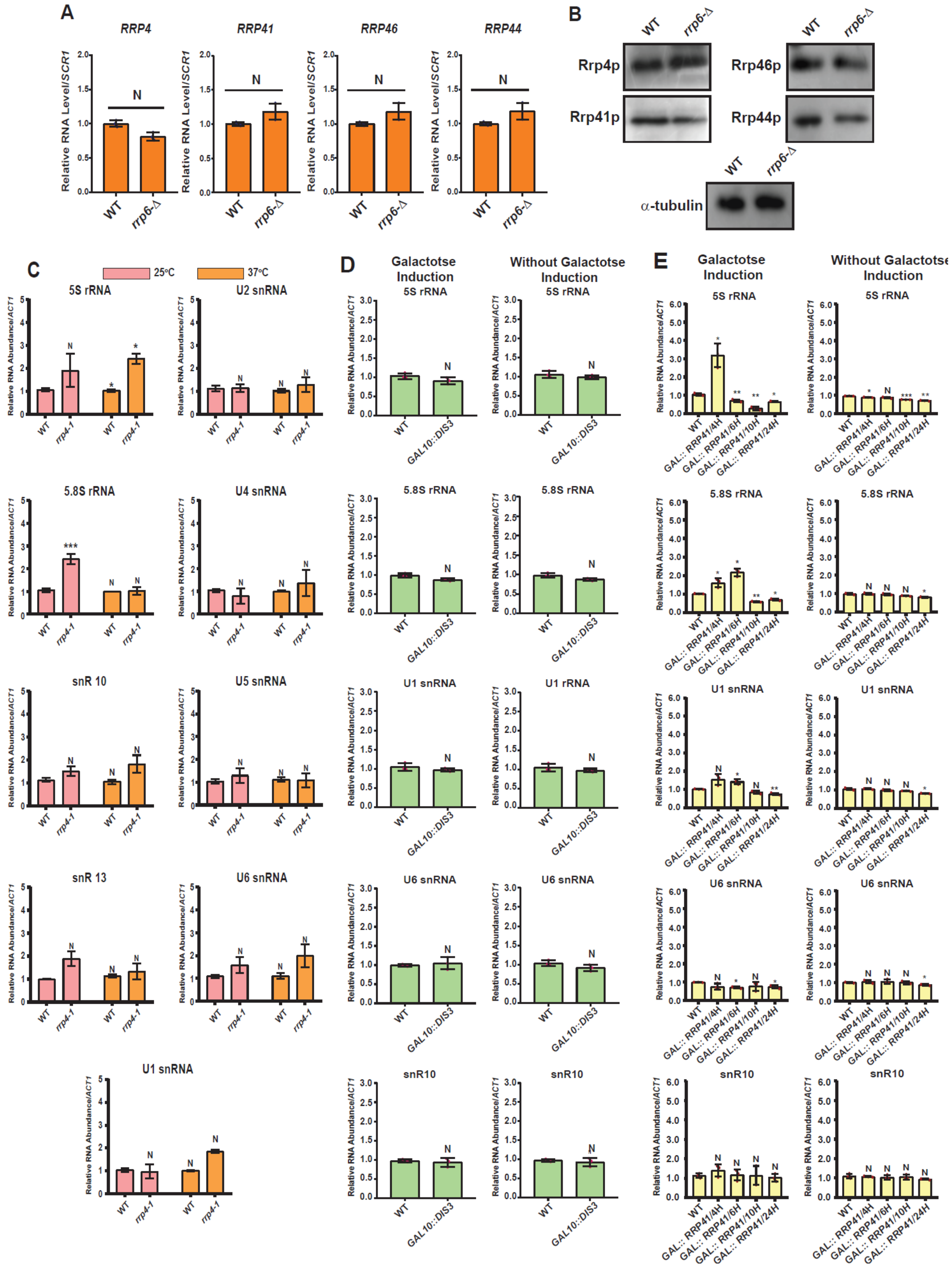

Supplementary Figure S3: (A-B) Absence of Rrp6p does not affect the levels of RNA and proteins of the components of core exosome. (A) Bar plots revealing the steady-state levels of the diverse mRNAs encoding different components of core exosome estimated from the 2 ng cDNA samples prepared from WT (yBD-263) and *rrp6*-Δ) (yBD-265) yeast strains by RT-qPCR assay. Normalized values of each of the ncRNAs in the wild-type yeast strain were set to one. (B) Relative levels of Rrp4p, Rrp41p, Rrp46p, Rrp44p and α-tubulin proteins in any of the following pair of strains: yBD-263 (WT)/yBD-265 (*rrp6*-Δ) (For Rrp4 and tubulin western Blot), yBD-508 (WT)/ yBD-541 (*rrp6*-Δ) (For

Rrp41-TAP western Blot), yBD-315 (WT)/ yBD-542 (*rrp6*-Δ) (For Rrp44-TAP western Blot) and yBD-543 (WT)/ yBD-544 (*rrp6*-Δ) (For Dis3p-TAP western Blot) as determined by western blot analysis. **(C) Steady-state levels of various small non-coding RNAs in yeast strains carrying mutant alleles of *RRP4* and *RRP41* genes at permissive and non-permissive temperatures.** Bar plot revealing the steady-state levels of various low molecular weight ncRNAs at 25°C and 37°C that were estimated from the 2 ng cDNA samples prepared using oligo-dT_{30} anchor primer by RT-qPCR from WT (yBD-263) and *rrp4-1* (yBD-285) yeast strains. The strains were pre-grown at 25ºC followed by splitting the culture into two halves. Half of the culture was continued to grow at 25°C for 6 h and a 6 h shift to 37ºC were performed to the other half of the culture before harvesting them. Total RNA and cDNA samples were prepared from each sample followed by the RT-qPCR assay using target specific-amplicons. **(D) Bar plot revealing the steady-state levels of various low molecular weight ncRNAs in WT (yBD-263) and *GAL10::DIS3* (yBD-545) yeast strains at 30°C.** The RNA signals were determined either by growing these strains in presence of raffinose/sucrose followed by induction with 2% galactose for 2 hours followed by growth in glucose medium for 24 hours (left histograms) or by growing in raffinose/sucrose without galactose induction till the OD_{600} of the culture reaches 0.9 to 1.0 followed by a growth in the glucose medium for 24 hours (right histograms). **(E) Bar plot revealing the steady-state levels of various low molecular weight ncRNAs in WT (yBD-263) and *GAL10::RRP41* (yBD-298) yeast strains at 30°C.** RNA signals were determined either by growing these strains in presence of raffinose/sucrose followed by induction with 2% galactose for 2 hours followed by growth in glucose medium for indicated times (left histograms) or by growing in raffinose/sucrose without galactose induction till the OD_{600} of the culture reaches 0.9 to 1.0 followed by a growth in the glucose medium for indicated times (right histograms). For panels D and E, signals were estimated from the 2 ng cDNA samples prepared using oligo-dT_{30} anchor primer by RT-qPCR from. *ACT1* mRNA were used as the internal loading control for normalization for both sets of experiments. Normalized values of each of the ncRNAs in the wild type yeast strain were set to one. For all the RT-qPCR experiments, three independent cDNA preparations (biological replicates, n = 3) were used to determine the levels of various ncRNAs. The statistical significance of difference reflected in the ranges of P values estimated from Student's two-tailed t tests for a given pair of test strains for every message are presented with the following symbols, *<0.05, **<0.005, and ***<0.001; NS, not significant.

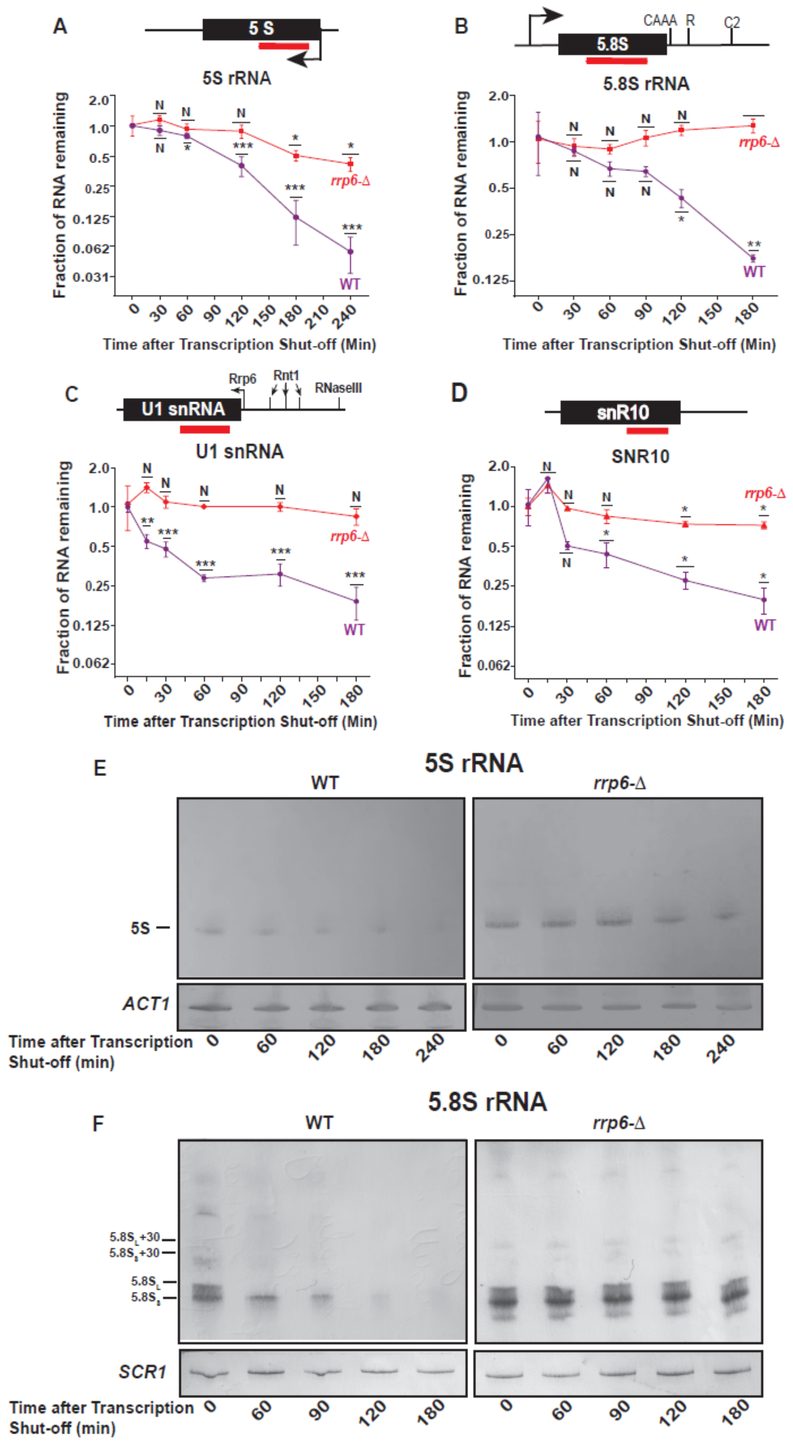

Figure S4: Transcription Shut-off experiments showing 5S, 5.8S, U1, and snR10 snRNAs undergo an active degradation by Rrp6p. (A-D) Decay Rates of mature forms of 5S (A), 5.8S rRNAs (B), U1 snRNA (C), and snR10 (D) in WT (yBD-263) and *rrp6*-Δ (yBD-265) strains following the transcription shut-off as determined by RT-qPCR (determined from four independent biological replicates, n=4). The RT-qPCR analysis was carried out using specific amplicons (shown in thick red lines below each gene sequence in top cartoons), and the signals were normalized to either *SCR1* RNA (in case of inhibition of RNA Pol-II) or *ACT1* mRNA (in case of inhibition of RNA Pol-I and III). Normalized signals (mean values ± SD) were presented as the fraction of remaining RNA (relative to normalized signals

at 0 min) as a function of time of incubation in the presence of either transcription inhibitors BMH-21 (for RNA Pol I Transcripts); and ML-60218 (for RNA Pol III Transcripts). The statistical significance of difference reflected in the signals from each time point in the ranges of P values are presented with the following symbols, *<0.05, **<0.005, and ***<0.001; N, not significant. (E-F) Decay of 5S (E) and 5.8S (F) rRNAs determined by northern blot analyses (one representative blot is shown out of three independent biological replicates, n=3). The northern blot was carried out with the DIG-labelled oligonucleotide probes corresponding to their mature sequence and the total RNA samples from WT and *rrp6*-Δ cells harvested at indicated time points following transcription shut-off as described in materials and methods.

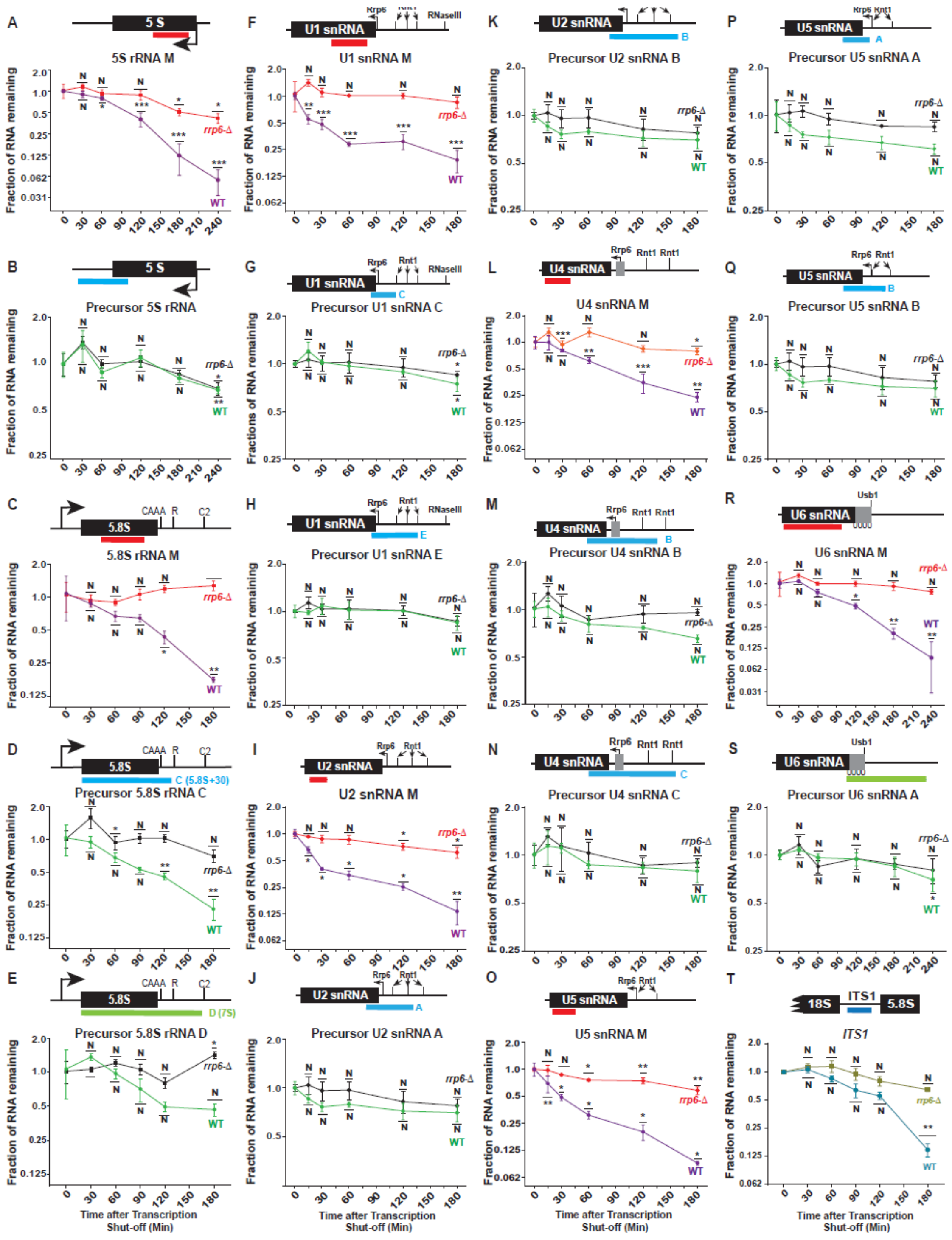

Figure S5: Transcription Shut-off experiments showing mature and various precursor populations of the 5S, 5.8S rRNAs and various snRNAs undergo an active degradation by Rrp6p. Decay Rates of mature and various precursors forms of 5S/5.8S rRNAs (panels A-E), various snRNAs (panels F-S) and ITS1 (panel T) in WT (yBD-263) and *rrp6*-Δ (yBD-265) strains following the transcription shut-off by RT-qPCR (determined from four independent biological replicates, n=4) using random primed cDNA prepared from cells harvested (WT and *rrp6*-Δ strains) at various times after transcription shut-off. The RT-qPCR analysis was carried out using specific amplicons (shown in thick red lines below each gene sequence in top cartoons), and the signals were normalized to either *SCR1* RNA (in case of

inhibition of RNA Pol-II) or *ACT1* mRNA (in case of inhibition of RNA Pol-I and III). Normalized signals (mean values ± SD) were presented as the fraction of remaining RNA (relative to normalized signals at 0 min) as a function of time of incubation in the presence of either transcription inhibitors BMH-21 (for RNA Pol I Transcripts); and ML-60218 (for RNA Pol III Transcripts). The statistical significance of difference reflected in the signals from each time point in the ranges of P values are presented with the following symbols, *<0.05, **<0.005, and ***<0.001; N, not significant.

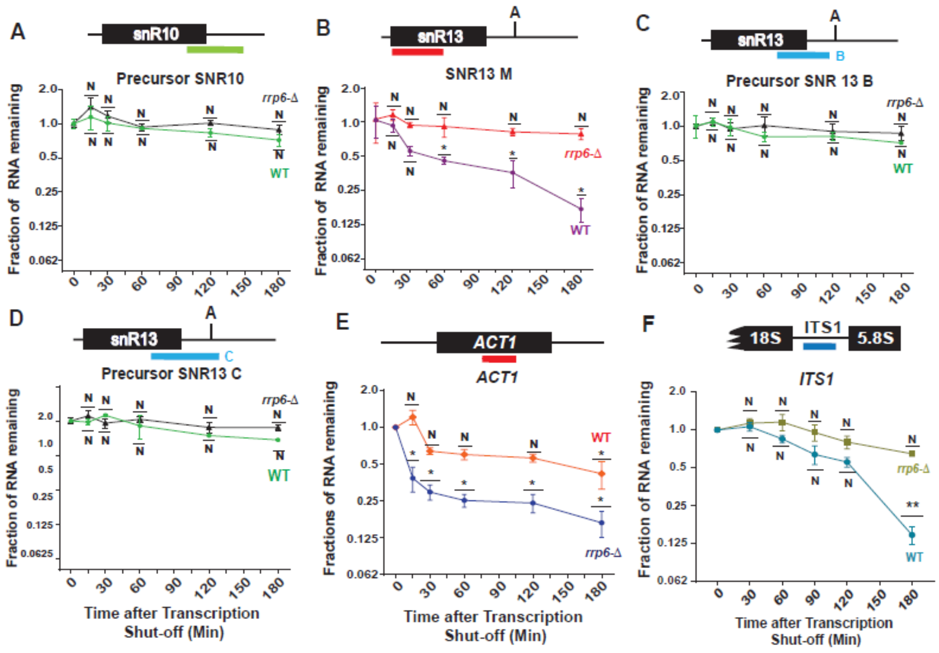

Figure S6: Transcription Shut-off experiments showing mature and various precursor populations of snR10, and snR13 RNAs undergo an active degradation by Rrp6p. (panels A-D) Transcription Shut-off experiments showing mature and precursor populations of the snR10 (panel A) and snR13 (panels B-D), *ACT1* (panel E) and ITS1 (panel F) undergo an active degradation by Rrp6p. Time kinetics of the steady-state levels of various precursor forms of mature and precursor forms of snR10 and snR13 (panels C-G) following the transcription shut-off revealing their decay rates in WT (yBD-263) and *rrp6*-Δ (yBD-265) strains. The decay rates were determined from four independent experiments (biological replicates, n=4) by RT-qPCR analysis using specific amplicons (shown in thick red/blue/green lines below each gene sequence in top cartoons), and the signals were normalized to *SCR1* RNA (in case of inhibition of RNA Pol-II). Normalized signals (mean values ± SD) were presented as the fraction of remaining RNA (relative to normalized signals at 0 min) as a function of time of incubation in the presence of transcription inhibitor 1, 10-phenanthroline (for RNA Pol II Transcripts). Time kinetics of the steady-state levels of *ACT1* mRNA is taken as control for RNA Pol II decay (panel F) to show that the drug worked normally in these experiments. The statistical significance of difference reflected in the signals from each time point in the ranges of P values are presented with the following symbols, *<0.05, **<0.005, and ***<0.001; N, not significant.

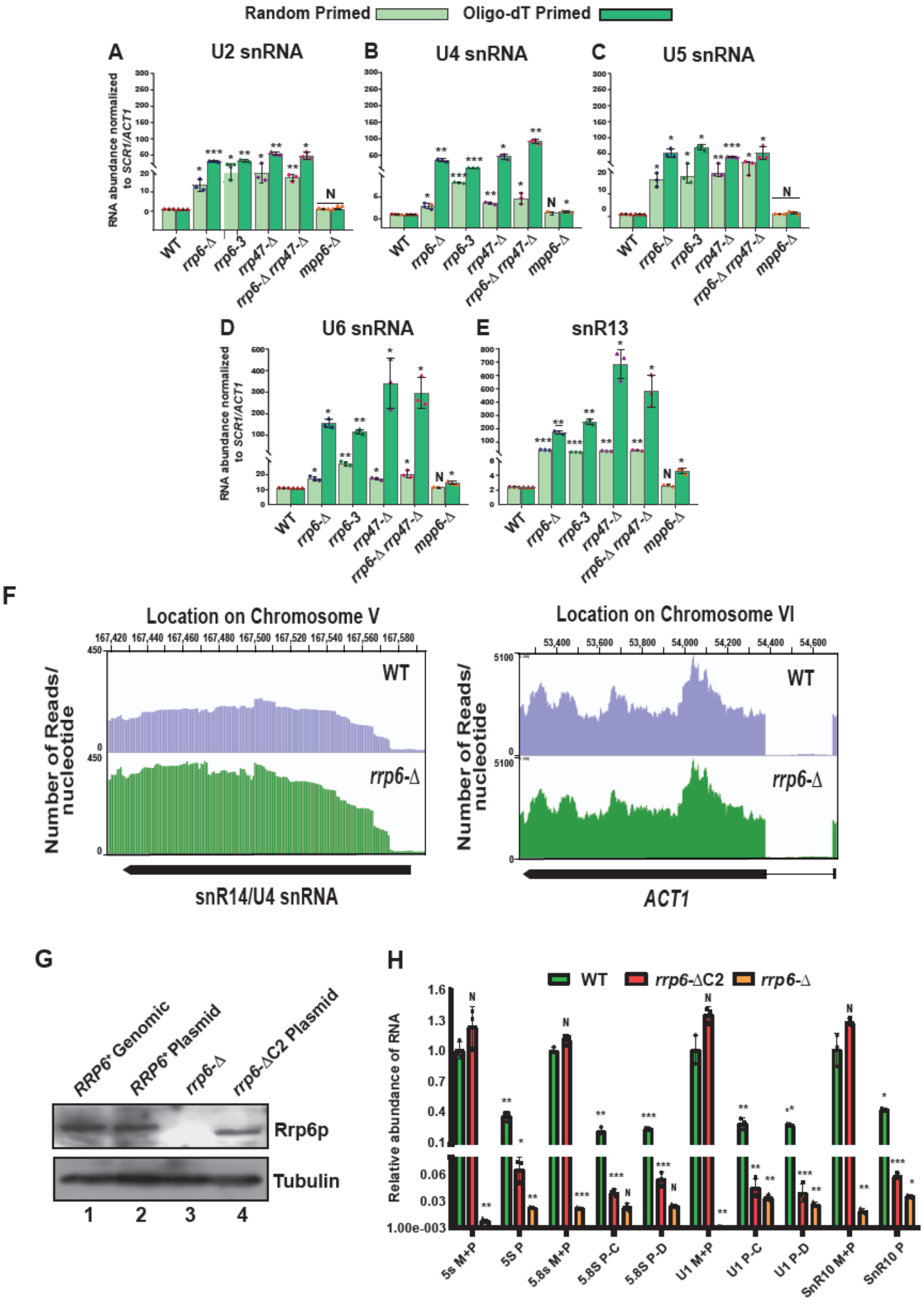

Figure S7: (A-E) Bar plots revealing the steady-state levels of total populations of U2 (A), U4 (B), U5 (C), U6(D), and snR13 (E) RNAs estimated by RT-qPCR from the 2 ng cDNA samples prepared using either random hexanucleotide primers (pale green bars) or oligo-dT30 anchor primer (deep green bars) from the isogenic WT (yBD-263) strain and strains carrying *rrp6*-Δ (yBD-265), *rrp6-3* (yBD-413), *rrp47*-Δ (yBD-454), and *mpp6*-Δ (yBD-455) alleles and *rrp6*-Δ *rrp47*-Δ double mutant alleles (yBD-510) together. *SCR1* (in the case of Random Primer) and *ACT1* mRNA (in the case of Oligo dT30 Primer) were used as the internal

loading control. Normalized values of each of the ncRNAs in the wild-type yeast strain were set to one. Three to Four independent cDNA preparations (biological replicates, n = 3, in some cases 4) were used to determine the levels of various ncRNAs. The statistical significance of difference reflected in the ranges of P values estimated from Student's two-tailed t-tests for a given pair of test strains for every message are presented with the following symbols, *<0.05, **<0.005, and ***<0.001; N, not significant. (F) Analysis of previous RNA-seq data (Accession Number GSE135056) revealed a dramatic accumulation of sense strand reads corresponding to the mature region of several small nucleolar RNAs. Graphical representation showing the relative amount of sense strand reads mapped to the genomic locus corresponding to *ACT1* and snR14/snRNA U4. The location of transcripts and the direction of transcription are shown below the graph (drawn in scale) by the solid black arrow-headed rectangles. (G) Immunoblots showing the relative expression levels of Rrp6p protein in WT (*RRP6*$^{+}$) strain expressing Rrp6p from a genomic copy (lane 1), WT strain expressing Rrp6p from *CEN* plasmid (lane 2), *rrp6*-Δ strain (lane 3), *rrp6*-ΔC2 strain expressing C-terminally truncated version of Rrp6p (lane 4) from *CEN* plasmid. Western blot was performed using 30 μg of total cellular protein extract prepared from a *RRP6*$^{+}$ and *rrp6*-ΔC2 yeast strains following the standard methods as described in the Materials and Methods. Tubulin was used as a loading control. (H) RNA immunoprecipitation experiment carried out with anti-Rrp6p antibody showing the interaction of various non-coding RNAs with Rrp6p/and the nuclear exosome. WT (yBD-117), *rrp6*-Δ (yBD-507), and *rrp6*ΔC2 (yBD-527) strains were grown at 30°C until the mid-log phase and subjected to UV-irradiation for crosslinking before harvesting. The cell extracts from each culture were prepared and immunoprecipitated with anti-Rrp6p antibody and the total RNA was isolated from IP, cDNA was prepared and each of the indicated non-coding RNA was detected and estimated using specific primer-sets/amplicons. Normalized (to input of each sample) Signal of mature 5S, 5.8S, U1 and snR10 RNA from WT strain was set to one and the normalized signals of mature species from other strains and from their precursor forms in all strains were expressed with respect to the mature signal in WT strain. Data were derived from three independent biological replicates (N = 3). For panels B and D, the statistical significance of difference reflected in the ranges of P values estimated from Student's two-tailed t-tests for a given pair of test strains for each message are presented with the following symbols, *<0.05, **<0.005, and ***<0.001; N, not significant.

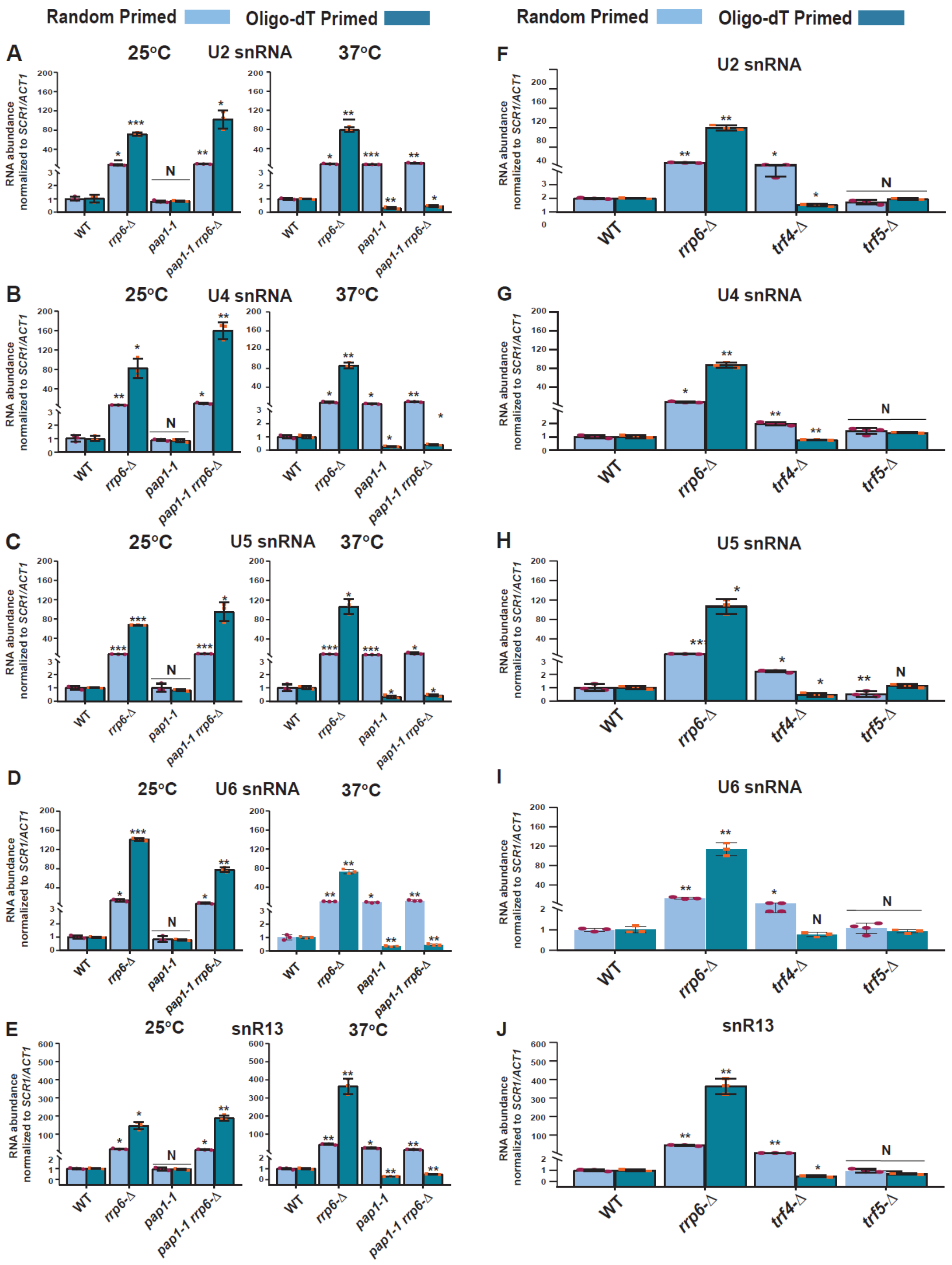

Supplementary Figure S8: Both the canonical Poly(A) polymerase Pap1p (Panels A-E) and non-canonical Poly(A) polymerase Trf4p (Panels F-J) play vital role in the polyadenylation of the small ncRNAs. (A-E) Bar plot revealing the steady-state levels of U2 (A), U4 (B) U5 (C), U6 (D) and snR13 (E) at 25°C and 37°C in the WT (*RRP6*, yBD-161) strains carrying mutations in *RRP6* (*rrp6*-Δ, yBD-162), *PAP1* (*pap1-1*, yBD-163), and *RRP6 PAP1* (*pap1-1 rrp6*-Δ, yBD-179) genes. (F-J) Relative steady-state levels of U2 (F), U4 (G) U5 (H), U6 (I) and snR13 (J) at 30°C in the WT (*RRP6*, yBD-263), and yeast strains carrying mutations in *RRP6* (*rrp6*-Δ, yBD-265), *TRF4* (*trf4*-Δ, yBD-306), and *TRF5* (*trf4*-Δ, yBD-334) genes. Steady-state levels were estimated from the 2 ng cDNA samples prepared from

above strains using random hexanucleotide primers (sky blue bars) or oligo-dT_{30} anchor primer (indigo blue bars) by RT-qPCR from WT and yeast strains carrying mutations. For panel A-C, the strains were pre-grown at 25ºC followed by splitting the culture into two halves. One half was continued to grow at 25°C for 2 hours and a 2h shift to 37ºC were performed to the other half of the culture before harvesting them. For panel D, the strains were grown at 30ºC followed by harvesting, isolation of total RNA, cDNA preparation followed by RT-qPCR reaction. *SCR1* (in case of Random Primer) and *ACT1* mRNA (in case of Oligo dT Primer) were used as the internal loading control. Normalized values of each of the ncRNAs in the wild type yeast strain were set to one. Three independent cDNA preparations (biological replicates, n = 3) were used to determine the levels of various ncRNAs. The statistical significance of difference reflected in the ranges of P values estimated from Student's two-tailed t tests for a given pair of test strains for every message are presented with the following symbols, *<0.05, **<0.005, and ***<0.001; NS, not significant.

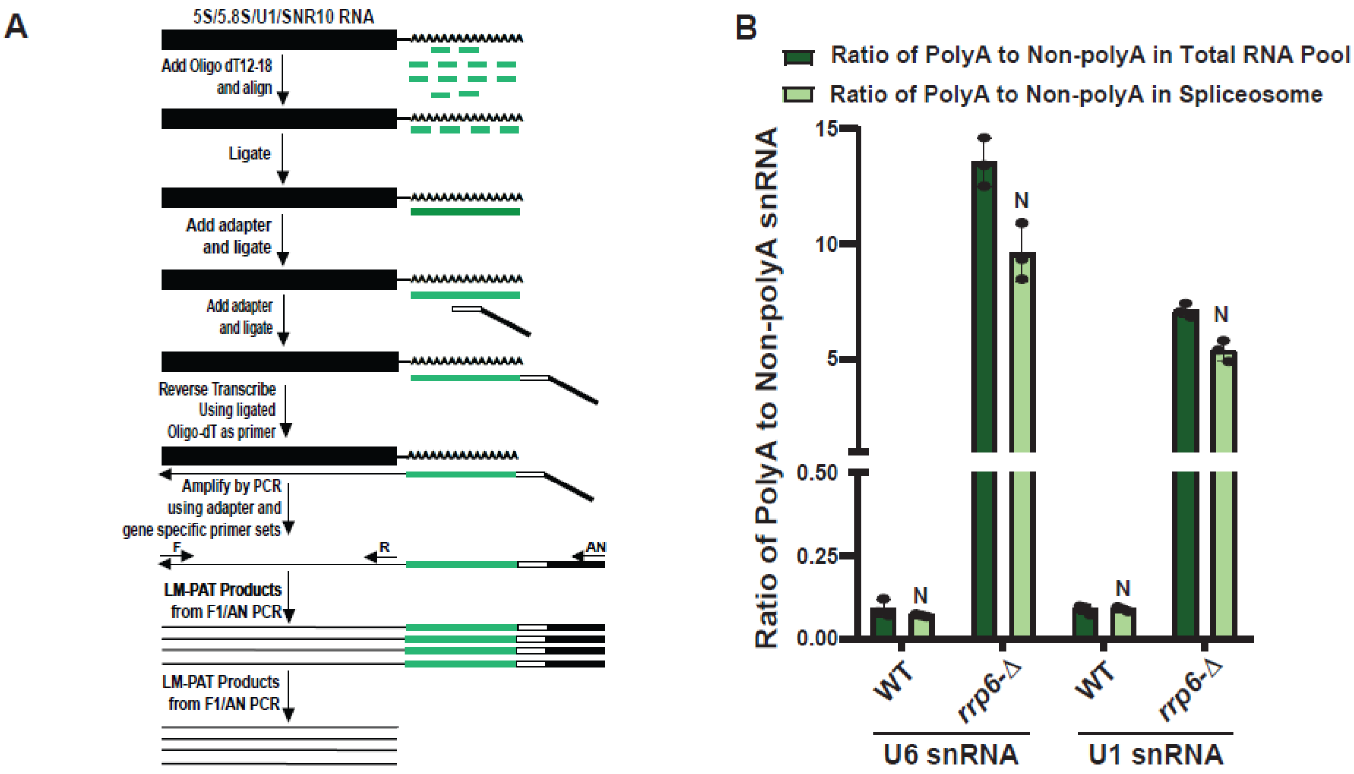

Figure S9. (A) Schematic diagram showing the steps involved in the ligation-mediated poly-A tail (LM-PAT) assay. A mixture of oligo-dT_{12-18} (indicated by small green lines) was mixed and allowed to align to the poly(A) tails of the total RNAs, which was followed by the ligation of the oligonucleotides at 42°C. An adapter (consisting of a small segment of oligo-dT and a unique sequence in tandem) was then ligated to the 5'-end of the ligated oligo-dT tail. The ligated adapter-oligo-dT was used as a primer for the first strand cDNA synthesis followed by the PCR amplification of the entire length of the poly (A) tail and 3'-UTR of a specific message. The primer sets used for the PCR consist of a sense primer that is complementary to a mature 5′-end of a specific RNA (F) and an antisense primer, which is either complementary to a mature 3′-end of a given RNA (R, F-R pair used when the mature sequence was amplified) or the complimentary to the unique region of the adapter (AN, F-AN pair used when the polyadenylated version was amplified). **(**B) Histogram showing the ratio of polyadenylated to non-polyadenylated U6 and U1 snRNAs estimated in the total cellular RNA pool (Deep green) and immunopurified spliceosomal preparation (Pale green) from WT and *rrp6*-Δ yeast strains. Abundance of polyadenylated and non-polyadenylated fractions of U6 and U1 RNAs in total RNA and immuno-purified spliceosome was performed as described before. *ACT1* mRNA were used as the internal loading control. Note that the P value of poly(A)/non-poly(A) ratio in spliceosome were estimated with respected to the same ratio in total cellular pool for a given snRNA in a specific strain.

REFERENCES

1. Das,B., Guo,Z., Russo,P., Chartrand,P. and Sherman,F. (2000) The Role of Nuclear Cap Binding Protein Cbc1p of Yeast in mRNA Termination and Degradation. *Mol. Cell. Biol.*, 10.1128/mcb.20.8.2827-2838.2000.

2. Maity,A., Chaudhuri,A. and Das,B. (2016) DRN and TRAMP degrade specific and overlapping aberrant mRNAs formed at various stages of mRNP biogenesis in Saccharomyces cerevisiae. *FEMS Yeast Res.*, **16**.

3. Briggs,M.W., Burkard,K.T. and Butler,J.S. (1998) Rrp6p, the yeast homologue of the human PM-Scl 100-kDa autoantigen, is essential for efficient 5.8 S rRNA 3' end formation. *J Biol Chem*, **273**, 13255–13263.

4. Patel,D. and Butler,J.S. (1992) Conditional defect in mRNA 3' end processing caused by a mutation in the gene for poly(A) polymerase. *Mol. Cell. Biol.*, **12**, 3297–3304.

5. Sarkar,D., Paira,S. and Das,B. (2018) Nuclear mRNA degradation tunes the gain of the unfolded protein response in Saccharo myces cerevisiae. *Nucleic Acids Res.*, **46**.

6. Stuparevic,I., Mosrin-Huaman,C., Hervouet-Coste,N., Remenaric,M. and Rahmouni,A.R. (2013) Cotranscriptional Recruitment of RNA Exosome Cofactors Rrp47p and Mpp6p and Two Distinct Trf-Air-Mtr4 Polyadenylation (TRAMP) Complexes Assists the Exonuclease Rrp6p in the Targeting and Degradation of an Aberrant Messenger Ribonucleoprotein Particle (mRNP) in Yeast. *J. Biol. Chem.*, **288**, 31816.

7. Bousquet-Antonelli,C., Presutti,C. and Tollervey,D. (2000) Identification of a regulated pathway for nuclear pre-mRNA turnover. *Cell*, **102**, 765–775.

8. Sikorski,R.S. and Hieter,P. (1989) A System of Shuttle Vectors and Yeast Host Strains Designed for Efficient Manipulation of DNA in Saccharomyces Cerevisiae. *Genetics*, **122**, 19.

9. Das,B., Guo,Z., Russo,P., Chartrand,P. and Sherman,F. (2000) The role of nuclear cap binding protein Cbc1p of yeast in mRNA termination and degradation. *Mol. Cell. Biol.*, **20**.

10. Das,B., Butler,J.S. and Sherman,F. (2003) Degradation of normal mRNA in the nucleus of Saccharomyces cerevisiae. *Mol. Cell. Biol.*, **23**.

11. Das,S., Saha,U. and Das,B. (2014) Cbc2p, Upf3p and eIF4G are components of the DRN (Degradation of mRNA in the Nucleus) in Saccharomyces cerevisiae. *FEMS Yeast Res.*, **14**.

12. Mitchell,P., Petfalski,E., Houalla,R., Podtelejnikov,A., Mann,M. and Tollervey,D. (2003) Rrp47p is an exosome-associated protein required for the 3' processing of stable RNAs. *Mol Cell Biol*, **23**, 6982–6992.

13. Das,S., Biswas,S., Chaudhuri,S., Bhattacharyya,A. and Das,B. (2019) A Nuclear Zip Code in SKS1 mRNA Promotes Its Slow Export, Nuclear Retention, and Degradation by the Nuclear Exosome/DRN in Saccharomyces cerevisiae. *J. Mol. Biol.*, **431**, 3626–3646.

- 14. Eckert D, Andrée N, Razanau A, Zock-Emmenthal S, Lützelberger M, Plath S, et al. (2016) Prp4 Kinase Grants the License to Splice: Control of Weak Splice Sites during Spliceosome Activation. *PLoS Genet 12*(1): e1005768. https://doi.org/10.1371/journal.pgen.1005768.
